# Global domain adaptation attention with data-dependent regulator for scene segmentation

**DOI:** 10.1371/journal.pone.0295263

**Published:** 2024-02-14

**Authors:** Qiuyuan Lei, Fei Lu

**Affiliations:** 1 School of Economics and Management, University of Science and Technology Beijing, Beijing, China; 2 Institute of Information Engineering, Nanyang Vocational College of Agriculture, Nanyang, China; Zhejiang University of Technology, CHINA

## Abstract

Most semantic segmentation works have obtained accurate segmentation results through exploring the contextual dependencies. However, there are several major limitations that need further investigation. For example, most approaches rarely distinguish different types of contextual dependencies, which may pollute the scene understanding. Moreover, local convolutions are commonly used in deep learning models to learn attention and capture local patterns in the data. These convolutions operate on a small neighborhood of the input, focusing on nearby information and disregarding global structural patterns. To address these concerns, we propose a **G**lobal **D**omain **A**daptation **A**ttention with Data-Dependent **R**egulator (**GDAAR**) method to explore the contextual dependencies. Specifically, to effectively capture both the global distribution information and local appearance details, we suggest using a stacked relation approach. This involves incorporating the feature node itself and its pairwise affinities with all other feature nodes within the network, arranged in raster scan order. By doing so, we can learn a global domain adaptation attention mechanism. Meanwhile, to improve the features similarity belonging to the same segment region while keeping the discriminative power of features belonging to different segments, we design a data-dependent regulator to adjust the global domain adaptation attention on the feature map during inference. Extensive ablation studies demonstrate that our GDAAR better captures the global distribution information for the contextual dependencies and achieves the state-of-the-art performance on several popular benchmarks.

## Introduction

**Fig 1:**
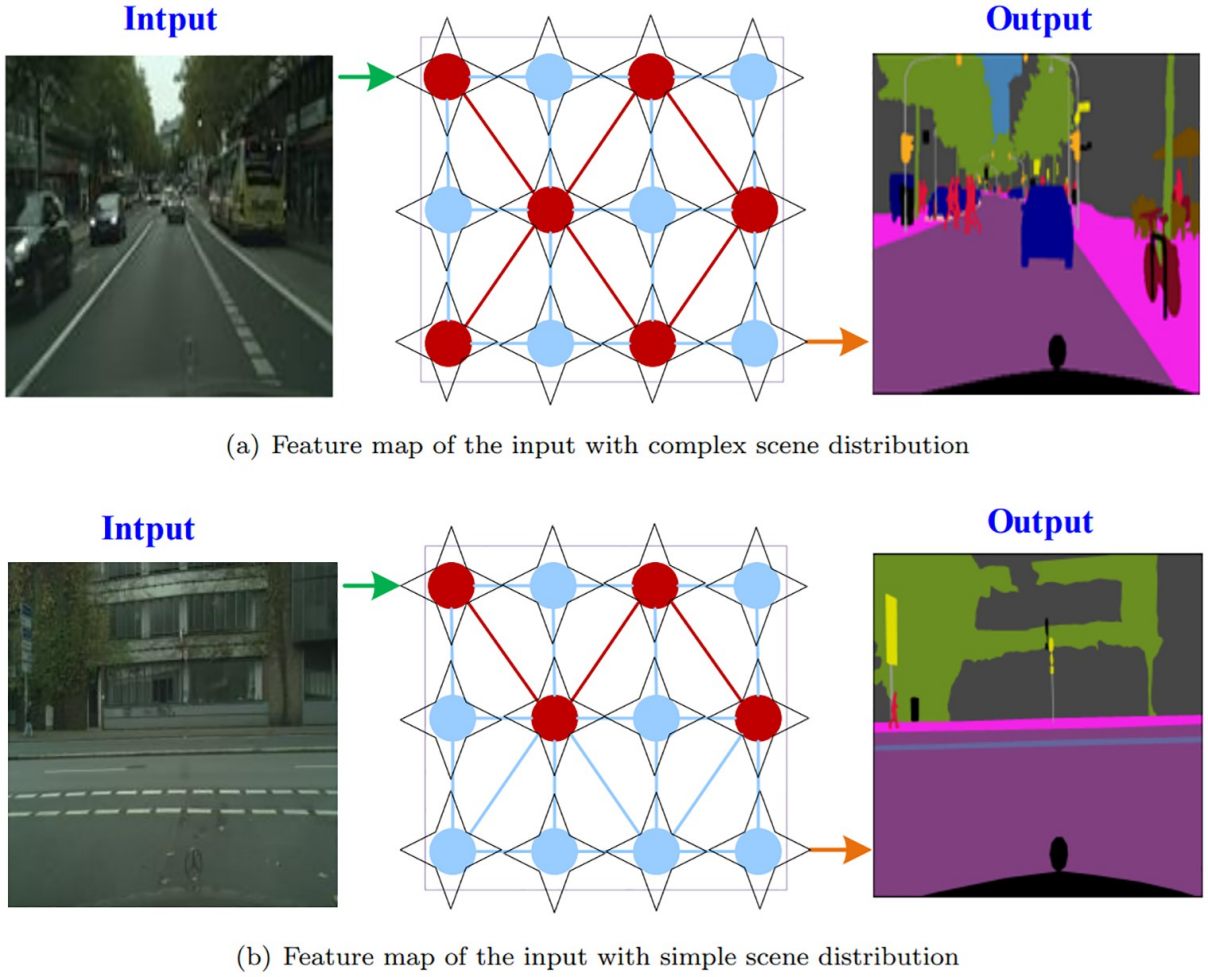
For different distributions of scenes, the proposed GDAAR adaptively employs different weights on the feature map. For instance, the feature map of input with complex scene distribution (a) may need more attention to obtain low-level details, and simple scene distribution (b) may same attention to get different-level features. Red circle and blue circle in diagrams denote the higher weight and the lower weight respectively, and black quadrangles denote the attention regulator of the corresponding feature node in feature maps.

Semantic segmentation is a computer vision technique that involves partitioning an image into multiple regions and assigning each region a label based on its semantics or meaning. This technique is used to classify and locate objects in an image and to provide more detailed information about the image than traditional pixel-level segmentation methods. For example, when applied to an image of a street, semantic segmentation can identify and label the different objects in the image such as cars, pedestrians, buildings, and trees. This technique is used in various applications such as autonomous vehicles [1, 2], medical imaging [3], and image recognition [4, 5]. As shown in Fig 1, the input images with diverse scene distributions will choose different attention for feature transformation, moreover, the regions of ‘field’ and ‘tree’ are often indistinguishable. Therefore, it is necessary to enhance the discriminative ability of feature representations for pixel-level recognition.

Several approaches have achieved promising performance by utilizing the effective feature representation of Fully Convolutional Network (FCN) [6]. However, due to the limitations of its convolutional structure, FCN often fails to provide sufficient contextual information, leaving room for improvement. As a result, various methods [4, 7–12] have been developed to explore contextual dependencies and improve the accuracy of segmentation results. In scene segmentation, there are two main methods to explore contextual information. One method is to utilize multi-scale context fusion. For example, several methods [5, 7, 8] employ pyramid-based modules or global pooling to aggregate regional or global contextual details. We propose an adaptation attention to extract the spatial relation and channel relation then aggregate them. In each relation (spatial or channel relation), we propose to stack the relations, i.e., the feature node itself and its pairwise affinities with all the feature nodes together to learn the adaptation attention. In such a way, we explore contextual dependencies of different categories from spatial and channel direction. However, some methods adopted pyramid-based modules or global pooling to explore contextual dependencies, which focus only on spatial relations and are limited on local appearance. In scenarios with chaotic categories, these methods may lead to unreliable contexts. In addition to methods that build long-range dependencies, some works [9, 11, 13] have also focused on capturing richer global context information by enlarging the kernel size with a decomposed structure or introducing an effective encoding layer into the network. Furthermore, encoder-decoder structures were proposed in works such as [3, 14, 15], aiming to fuse intermediate and advanced semantic features. By combining these approaches, researchers have been able to enhance the ability of semantic segmentation models to capture detailed and comprehensive contextual information from images, enabling more accurate and robust segmentation results in various settings. Although context fusion helps to capture objects of different scales, it rarely utilizes the relationships between objects or things in the global view, which is also crucial for scene segmentation.

Another approach involves using global context modeling [4, 5, 8, 16, 17] to investigate long-distance associations, which enhances the accuracy of scene segmentation. As an illustration, some previous attempts such as non-regional network [16] and dual attention network [18] aimed to incorporate novel interaction units that can perceive the entire space in terms of time and location. In dynamic graph convolutional recurrent imputation network [19], the usage of a graph generator and dynamic graph convolutional gated recurrent unit for fine-grained modeling of dynamic spatiotemporal dependencies in road networks is an effective approach. These efforts expanded the coverage area and facilitated the identification of long-distance correlations throughout complex neural networks. Recurrent neural networks (RNNs) [20, 21] may also be utilized for long-term decision-making. However, all of the aforementioned approaches focus on depicting each instance using adjacent features, and there is still a need for more effective methods to model global context and perform explicit scene semantics inference.

**Fig 2:**
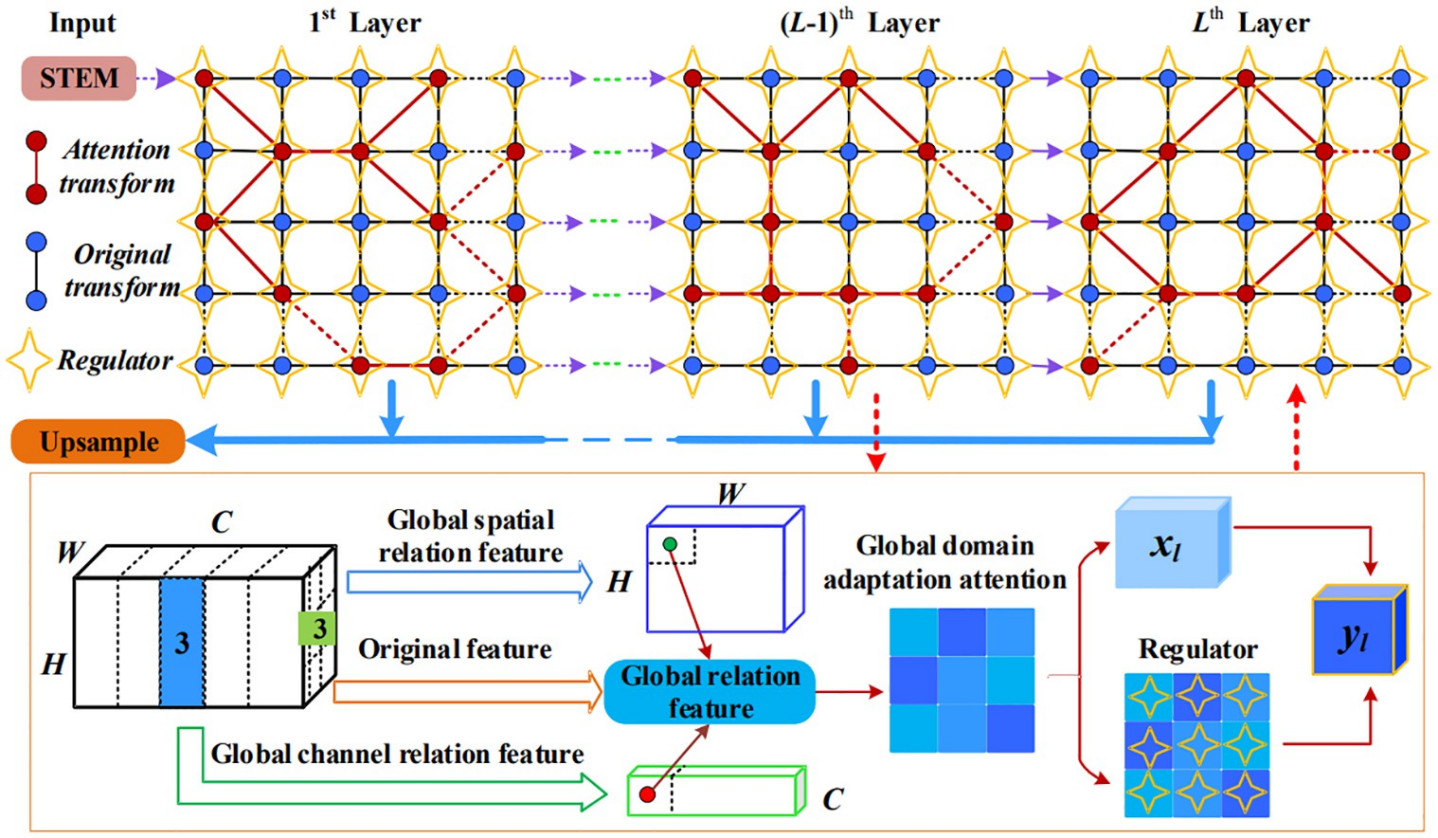
The proposed global domain adaptation attention with data-dependent regulator framework for scene segmentation. To ensure stability in the system, it is common practice to fix certain components in the architecture, such as the beginning STEM (preprocessing) and the final upsample block. Top: The global domain adaptation attention space with feature maps of *L* layers. The dotted lines among red circles or blue circles mean same feature transformations from *N* feature nodes. Red circles and blue circles denote feature transformations, and yellow quadrangles denote the regulator of the corresponding feature node on feature maps. Bottom: Given the input from the former layer, we first generate global domain adaptation attention through obtaining global scope relations. To further improve the performance of our approach, we design a data-dependent regulator that adjusts the attention weight on the feature map during inference. By incorporating this additional mechanism, our model is able to better adapt to diverse input domains and enhance segmentation accuracy for challenging images.

To combat the aforementioned challenges, we present a method called Global Domain Adaptation Attention with Data-Dependent Regulator (**GDAAR**) for scene segmentation. It is depicted in Fig 2. To elaborate further, spatial nodes on the feature map exhibit affinities that can be leveraged to obtain clustering-like information, which in turn aid in semantic inference. For each feature node, we seek to capture global distribution information and local appearance information in a concise manner by examining its pairwise relationships with all other nodes. We then stack these relations as a vector to represent the global distribution information. We then fuse the global scope relations and the feature node itself to infer the global domain adaptation attention. By imposing a constraint on the global domain adaptation attention, we can further adjust the corresponding attention weight on the feature node via the data-dependent regulator for feature transformation. This approach enables us to consider both the appearance information of each feature node and its global scope relations in determining the feature importance from a global perspective. Such a mechanism is also in line with human perception when identifying distinctive features, which involves making a comparison across a global view to determine their importance.

We demonstrate that our proposed GDAAR method achieves state-of-the-art performance on three benchmark datasets, namely Cityscapes [22], PASCAL Context dataset [23] and COCO Stuff dataset [24]. Through extensive ablation experiments, we also establish the efficacy of the proposed method in modeling rich contextual dependencies. In summary, the main contributions of this paper are as follows:

- We propose a Global Domain Adaptation Attention with Data-Dependent Regulator (GDAAR) method for scene segmentation, which significantly enhances the segmentation performance by modeling rich contextual dependencies across global scope relations.
- To achieve the aforementioned goals, we have designed a global domain adaptation attention module that aggregates both global spatial relation (GSR) and global channel relation (GCR) to represent the global scope relations in a compact manner. This enables the better differentiation of different types of contextual dependencies from a global perspective. The global domain adaptation attention is derived based on these relations through two convolutional layers. Overall, this module contributes significantly to our proposed GDAAR method for scene segmentation.
- In addition to the global domain adaptation attention, we also designed a data-dependent regulator that adjusts the attention weight on the feature map for customized feature transformation. This results in improved feature similarity for regions belonging to the same segment while retaining the discriminative power of features belonging to different segments. The use of this regulator is a major factor in achieving significant improvements in segmentation performance with our proposed GDAAR method.

## Related work

In this section, we provide a brief review of two related topics: scene segmentation and self-attention modules.

### Scene segmentation

Scene segmentation is a fundamental problem in computer vision and has attracted significant attention. Fully convolutional network (FCN) based methods have achieved great success in this field; however, the architecture of FCNs can lead to issues such as rough edges and inconsistent predictions. To address these problems, recent research has focused on employing contextual information aggregation techniques that leverage long-range dependencies within images. These methods aim to improve the accuracy and consistency of segmentation results by considering the surrounding context of each pixel or region.

There are several examples of methods that have incorporated contextual information aggregation to improve scene segmentation performance. ParseNet [25] utilized global image-level information by concatenating global features to each pixel, while DeepLabv2 [26] introduced the atrous spatial pyramid pooling (ASPP) module which consisted of parallel dilated convolutions with varying dilation rates to capture multi-scale contextual information. Similarly, PSPNet [4] utilized spatial pyramid pooling to collect contextual information at different scales. DeepLabv3 [27] extended the ASPP module by applying it to image-level features in order to better aggregate global context. Additionally, GCN [9] adopted a large kernel with a decomposed structure to obtain a larger receptive field for capturing more global context. Lastly, EncNet [11] designed a context encoding module that captures semantic context of the scene and selectively highlights class-dependent feature maps. These methods demonstrate the effectiveness of aggregating contextual information for improving scene segmentation accuracy, but they often suffer from limitations in terms of efficiency and multi-scale context aggregation. To address these limitations, we propose a global domain adaptation attention mechanism that provides a more efficient and effective way to aggregate contextual information across multiple scales. By incorporating this mechanism into our GDAAR method, we are able to improve the accuracy and reliability of segmentation results significantly.

There have been recent efforts to incorporate long-range dependencies into scene segmentation methods in order to better model global context. These approaches include advanced convolution operations [16, 18, 28], conditional random fields (CRFs) [29, 30], and variants of recurrent neural networks (RNNs) [20, 21, 31]. For instance, DAG-RNN [20] uses a generalization of RNNs to pass local context based on directed acyclic graphs, thereby capturing rich contextual dependencies over regions within the image. While these methods show promise in improving global context modeling, they still require further development to efficiently infer scene semantics. Our proposed GDAAR method takes a different approach by modeling rich contextual dependencies using global domain adaptation attention, which allows for more effective and efficient differentiation between various types of contextual dependencies from a global perspective. By leveraging this mechanism, our method yields significant improvements in segmentation performance while also being highly scalable and computationally efficient.

### Self-attention modules

Attention modules have the ability to model long-range dependencies and have been successfully applied in a wide range of tasks [32–35]. This makes them particularly well-suited for addressing the challenges involved in scene segmentation, including the need to capture rich contextual information across multiple scales. The use of attention modules in our proposed GDAAR method allows us to effectively model relationships between different regions within an image, leading to significant improvements in segmentation accuracy. Overall, attention-based approaches represent a powerful and attractive technique for overcoming some of the key challenges associated with scene segmentation. For instance, the SAGAN method [36] introduced self-attention mechanism into the generation process to learn better image generators. The Non-Local Network [16] used a self-attention module to explore the effectiveness of non-local operations in spacetime dimensions. There are numerous examples of attention-based approaches being used in related computer vision tasks. For object detection, the Relation Network [37] leverages an adapted attention module to model the relative geometry between objects. Meanwhile, for scene segmentation, methods such as DANet [38] use two types of attention modules to represent intra-class compactness features. A^2^Net [18] employs weighted global pooling and broadcasting to gather and distribute contextual information, while CCNet [39] stacks two crisscross attention modules in a recurrent fashion. EMANet [40] uses the EM algorithm to obtain a compact basis set and iteratively distribute the set for improved context modeling. Finally, the Knowledge-aware network [41] employs a parameter-free spatial attention mechanism that effectively captures significant and detailed information. These approaches highlight the versatility and effectiveness of attention-based techniques across a wide range of computer vision tasks. However, these approachs may not be fully sufficient for capturing the complex contextual information necessary to accurately infer scene semantics. In contrast, our proposed GDAAR method aims to explore the respective global scope relations for each feature node, allowing us to learn a more dynamic and adaptive form of attention that better captures the nuances of the underlying scene. By leveraging this global domain adaptation attention, we can improve our ability to model global context and ultimately achieve more accurate and robust scene segmentation results.

## Method

This section details the description of our framework, named Global Domain Adaptation Attention with data-dependent Regulator (**GDAAR**), which is illustrated in Fig 2. The framework focuses on two perspectives to infer semantics in scene segmentation: 1) Compared with attention mechanism over local features, we design a global domain adaptation attention module, where the spatial relation and channel relation are compactly aggregated to represent the global scope relation, thus obtaining the global domain adaptation attention via the global scope relation. Therefore, the global domain adaptation attention better distinguishes different types of contextual dependencies through global structure patterns. 2) With global domain adaptation attention constraint, we design a data-dependent regulator to adjust the attention weight on the feature map during inference. In particular, we employ criss-cross features to generate a data-dependent regulator, thus adjusting the corresponding attention weight on feature nodes for customized feature transformation. Therefore, the global domain adaptation attention has superiority than adopting local convolutions to typically learn attention for different scene segmentation (*i*.*e*. complex scenes or simple scenes). Moreover, one key advantage of the global domain adaptation attention mechanism in our proposed GDAAR method is that it enables us to improve the similarity between features belonging to the same segment region, while preserving the discriminative power of features belonging to different segments from a global perspective. This helps to ensure that we are able to accurately distinguish between different object classes within an image, even in cases where they may share certain visual characteristics or contextual relationships. Overall, this approach provides a more effective and efficient way to model complex global context and achieve highly accurate scene segmentation results. In this section, we first introduce the **GDAAR** architecture. And then, the global domain adaptation attention and the data-dependent regulator are elaborated.

### The GDAAR architecture

As illustrated in Fig 2, we propose a global domain adaptation attention with data-dependent regulator to model rich contextual dependencies from a global view, namely **GDAAR**. The backbone of our GDAAR framework is built upon the widely-used ResNet-50 and ResNet-101 architectures [42], which have demonstrated excellent performance across a wide range of computer vision tasks. By leveraging these established architectures, we are able to build a robust and scalable framework that can effectively handle complex scene segmentation problems. We then integrate our proposed global domain adaptation attention mechanism into this backbone architecture, allowing us to better model context and achieve more accurate and robust segmentation results than would be possible with the ResNet models alone. Specifically, following the common practices in the network design, the beginning of our framework is a fixed 3-layer ‘STEM’ block, which reduces the resolution to 1/4 scale. After that, a space with *L* layers is designed for customized feature transformation. In feature transformation process, our method intends to explore the respective global scope relations for each feature node, where the spatial relation and channel relation are compactly aggregated to represent the global scope relations. And deriving the global domain adaptation attention mechanism in our GDAAR framework, we use two convolutional layers that are built on top of the ResNet-50 or ResNet-101 backbone. These layers allow us to distinguish different types of contextual dependencies from a global perspective, effectively capturing the nuances of complex scene semantics. By learning these global domain adaptation attentions, we can better model the complex relationships and interactions between objects within an image, leading to more accurate and contextually relevant segmentation results. Meanwhile, we propose a data-dependent regulator, which employs the contextual information in horizontal and vertical directions to adjust the corresponding attention weight on feature nodes, thus increasing the feature similarity of the same object while keeping the feature discrimination of different objects. Moreover, a naive decoder is designed for final predictions, which is represented as blue arrows at the middle in Fig 2. Specifically, a conv 1 × 1 combined with bilinear up-sampling is used to fuse features from different layers in the decoder. And the weights in convolutions are initialized with normal distribution [43].

### Global domain adaptation attention

In some previous works (such as [4, 9]), traditional fully convolutional networks (FCNs) have been used to obtain local features for semantic segmentation. However, these approaches may be prone to misclassification of objects and background regions due to the limited context that is captured by the local features. This can result in inaccurate or incomplete segmentations, particularly in cases where objects or regions have highly variable visual characteristics or contextual relationships. In contrast, our proposed GDAAR framework leverages a global domain adaptation attention mechanism built on top of an established ResNet backbone, allowing us to more effectively model complex contextual relationships and capture the nuances of scene semantics. By adopting this approach, we can achieve significantly more accurate and robust segmentation results than would be possible with traditional FCN-based methods.

Given an intermediate feature tensor *X* ∈ *R*^*C*×*H*×*W*^, where *C* represents the number of channels, *H* represents the height dimension, and *W* represents the width dimension, we design a Global Domain Adaptation Attention based on the global scope relations, namely **GDAA**, where global scope relations include the global spatial position (*W*×*H*) and channel *C* relations. To infer the global domain adaptation attention of the *i*th feature node, besides the pairwise relation items *R*_*i*_, we also include the feature node itself *x*_*i*_ to exploit both the global scope distribution information relative to this feature node and the local original information. Therefore, a dot-product affinity is used to calculate the global scope relation of the *i*th feature node, which is determined in the embedding spaces as follows:

$$\begin{array}{c} R_{ij}=H_{1}{\left(x_{i}\right)}^{T}H_{2}\left(x_{j}\right)+H_{2}{\left(x_{j}\right)}^{T}H_{1}\left(x_{i}\right) \end{array}$$ (1)

where *i*, *j* = 1, ⋯, *N*, *H*_1_ and *H*_2_ are two embedding functions implemented by a 1×1 convolutional layer followed by batch normalization (BN) and ReLU activation, *i*.*e*., *H*_1_(*x*_*i*_) = ReLU(*W*_1_*x*_*i*_), *H*_2_(*x*_*i*_) = ReLU(*W*_2_*x*_*i*_), *R*_*ij*_ represents the pairwise relation (*i*.*e*. affinity) from node *i* to node *j*. Similarly, we can get the affinity from node *j* to node *i* as *R*_*ji*_. We use the pair (*R*_*ij*_, *R*_*ji*_) to describe the bi-directional relations between *x*_*i*_ and *x*_*j*_. Then, we represent the pairwise relations among all the nodes by an affinity matrix *R*_matrix_ ∈ *R*^*N*×*N*^.

Taking the global spatial relation features as an example. Since the feature node itself *x*_*i*_ and pairwise relation items *R*_*i*_ are not in the same feature domain, we need to embed them separately to allow for comparison and analysis. To achieve this, we use a set of embedding layers that convert *x*_*i*_ and *R*_*i*_ into distinct feature spaces. We then concatenate these embeddings to obtain global spatial relation features that can be used to better model the contextual relationships between objects within an image. The global spatial relation features is represented as follows:

$$\begin{array}{c} F_{gs}^{i}=\left[\text{Pool}\left(\text{ReLU}\left(W_{x}x_{i}\right)\right),\text{ReLU}\left(W_{R}R_{i}\right)\right] \end{array}$$ (2)

where *W*_*x*_ and *W*_*R*_ are implemented by a 1×1 convolution followed by BN, Pool(·) denotes global average pooling operation. The global channel relation features $F_{gc}^{i}$ are similar to the derivation of the global spatial relation features, where no fully connected operation across channels.

The global scope relations contain affluent distribution information (e.g., clustering-like state in feature space with semantics), we use a modeling function to mine valuable knowledge from them, thus inferring global domain adaptation attention value *A*_*i*_ of the *i*th feature node as:

$$\begin{array}{c} F_{g}^{i}=\left[\text{Pool}\left(\text{ReLU}\left(W_{gs}F_{gs}^{i}\right)\right),\text{ReLU}\left(W_{gc}F_{gc}^{i}\right)\right] \end{array}$$ (3)

$$\begin{array}{c} A_{i}=\text{Sigmoid}\left(W_{2}\text{ReLU}\left(W_{1}F_{g}^{i}\right)\right) \end{array}$$ (4)

where $F_{gs}^{i}$ and $F_{gc}^{i}$ represent the global spatial and channel relation features respectively, considering these two kinds of information are not in the same feature domain, similar to Eq (2), we embed them respectively and concatenate them to get global scope relation features $F_{g}^{i}$. *W*_1_ and *W*_2_ are implemented by a 1×1 convolution followed by BN. We represent the global domain adaptation attention value among all the nodes by an attention matrix *A*_matrix_ ∈ *R*^*C*×*H*×*W*^. Note that all the transformation functions are shared by feature nodes.

### The data-dependent regulator

To increase the feature similarity of the same object while keeping the feature discrimination of different objects, based on the global domain adaptation attention, we introduce a data-dependent regulator to adjust the attention value on the feature map during inference. The data-dependent regulator collects contextual information in horizontal and vertical directions to enhance pixel-wise representation capability. Given an intermediate feature tensor *X* ∈ *R*^*C*×*H*×*W*^, the regulator firstly applies two convolutional layers with 1×1 filters on *X* to generate two feature maps *Q* and *K* respectively, where *Q*, *K* ∈ *R*^*C*′×*H*×*W*^. *C*′ is the number of channels, which is less than *C* for dimension reduction.

After obtaining feature maps *Q* and *K*, we further generate regulator maps *A*′ ∈ *R*^(*H*+*W*−1)×*H*×*W*^ via the affinity operation. At each position *i* in the spatial dimension of feature maps *Q*, we can obtain a vector $Q_{i}\in R^{C^{\prime }}$. Meanwhile, we can also obtain the set $\Phi_{i}\in R^{\left(H+W-1\right)\times C^{\prime }}$ by extracting feature vectors from *K* which are in the same row or column with position *i*. $\Phi_{j,i}\in R^{C^{\prime }}$ is the *j*th element of Φ_*i*_. The affinity operation is then defined as follows:

$$\begin{array}{c} d_{j,i}=Q_{i}\Phi_{j,i}^{T} \end{array}$$ (5)

where *d*_*j*,*i*_ ∈ *D* is the degree of correlation between feature *Q*_*i*_ and Φ_*j*,*i*_, *j* = 1, ⋯, |Φ_*i*_|, *D* ∈ *R*^(*H*+*W*−1)×*H*×*W*^. Then, we apply a softmax layer on *D* over the channel dimension to calculate the regulator map *A*′. Once obtaining the regulator map *A*′, we further adjust the global domain adaptation attention value *A*_*j*,*i*_ at channel *j* and position *i* via the regulator $A_{j,i}^{\prime }$ as follows:

$$\begin{array}{c} GA_{j,i}=\frac{A_{j,i}+A_{j,i}^{\prime }}{\sum_{j\in |\Phi_{i}|,i\in D}\left(A_{j,i}+A_{j,i}^{\prime }\right)} \end{array}$$ (6)

After obtaining the new value *GA*_*j*,*i*_ of global domain adaptation attention, at each position *i* in the spatial dimension of feature maps *X*, we can obtain a vector *X*_*i*_ ∈ *R*^*C*^ and a set *O*_*i*_ ∈ *R*^(*H*+*W*−1)×*C*^. The set *O*_*i*_ represents a collection of feature vectors in *X* that are located in the same row or column position as the target feature vector $X_{i}^{\prime }$. By collecting these related features, we can better model the contextual relationships between objects within an image and capture subtle nuances of scene semantics. These related features provide valuable information about the local spatial arrangement and orientation of objects within the image, which is critical for accurate semantic segmentation. The contextual information is represented as follows:

$$\begin{array}{c} X_{i}^{\prime }=\sum_{j\in |O_{i}|}GA_{j,i}O_{j,i} \end{array}$$ (7)

where $X_{i}^{\prime }$ is a feature vector in output feature maps *X*′ ∈ *R*^*C*×*H*×*W*^ at position *i*. *GA*_*j*,*i*_ is a scalar value at channel *j* and position *i* in *GA*. Therefore, it has a wide contextual view and selectively aggregates contexts according to the global domain adaptation attention map. These feature representations achieve mutual gains and are more robust for scene segmentation.

## Experiments

In this section, we conduct extensive experiments to validate the effectiveness of our proposed method. First, we introduce the datasets used for training and evaluation and describe the implementation details. Next, we perform ablation studies to analyze the performance of different components. Finally, our method is compared with state-of-the-art methods on several benchmarks.

### Datasets and implementation details

#### Dataset settings

To evaluate the proposed method, we carry out comprehensive experiments on three datasets as follows.

The Cityscapes dataset [22] is a commonly used dataset for scene segmentation in computer vision research. This dataset consists of 5000 high-resolution images that have been finely annotated with semantic labels for 19 different categories such as road, sidewalk, building, and vegetation. These images have dimensions of 2048×1024 pixels and are split into three sets: 2975 images for training, 500 images for validation, and 1525 images for testing. In our experiments, we do not make use of the coarse data that is also provided in the dataset. By using this large and diverse dataset for evaluation, researchers can more accurately assess the performance of their models and compare them against state-of-the-art approaches in the field of semantic segmentation.

The PASCAL Context dataset [23] is another commonly used benchmark for semantic segmentation. This dataset provides finely detailed semantic labels for entire scenes, with annotations for over 400 different object categories. The dataset consists of 10,103 high-resolution images, with 4998 images provided for training and 5105 images for testing. In our experiments, we follow the approach used in previous works [11, 15] and evaluate our method on the 60 most frequent object categories, including one background category. These 60 classes provide a comprehensive and representative set of object categories that are commonly encountered in real-world scenes. By evaluating our method on this challenging and diverse dataset, we can more accurately assess its ability to model complex contextual relationships and capture the nuances of scene semantics.

The COCO Stuff dataset [24] is a large-scale benchmark for scene understanding and semantic segmentation. This dataset consists of 10,000 high-quality images, with 9000 images provided for training and 1000 images for testing. The dataset includes annotations for 171 different object categories, including 80 different objects and 91 different materials or textures, all of which are labeled at the pixel level. In our experiments, we follow the approach used in previous works [14] and evaluate our method on all 171 categories. By reporting results on a large and diverse set of object categories and material labels, we can more accurately assess the generalization ability of our model and compare it against state-of-the-art approaches in the field of semantic segmentation.

#### Implementation details

In this section, we provide details on our optimization strategy for the convenience of implementation. Our approach is similar to that used in previous works [11, 27], where we employ a polynomial learning rate policy. Specifically, we start with an initial learning rate and then multiply it by ${\left(1-\frac{iter}{iter_{max}}\right)}^{0.9}$ in each iteration. This polynomial decay schedule has been shown to be effective in many computer vision tasks, as it allows the learning rate to decrease gradually over time, which can result in improved model performance and stability. By using this approach, we can more effectively optimize our model and achieve state-of-the-art results on benchmark datasets such as Cityscapes, PASCAL Context, and COCO Stuff.

In training stage, the base learning rate is set to 0.01 for Cityscapes dataset, momentum and weight decay coefficients are set to 0.9 and 0.0001 respectively. Moreover, the batch size is set to 8 for Cityscapes and 16 for other datasets. We set training time to 180 epochs for COCO Stuff and 240 epochs for other datasets. Following the approach used in previous works [38], we employ random cropping and left-right flipping during training to improve model performance. For the Cityscapes dataset, we randomly crop images to a size of 768 pixels and apply random left-right flipping. For other datasets, we use a random cropping size of 512×512 pixels. These data augmentation techniques have been shown to be effective in improving model robustness and generalization by increasing the diversity of training data. By including these techniques in our training pipeline, we can better account for variability in input image sizes and orientations, resulting in a more versatile and reliable model.

The performance of full supervised segmentation is evaluated using mean intersection over union (mIoU). Assuming that the real segmentation target is *Target* and the predicted segmentation target is *Prediction*, the definition of intersection to union ratio is as follows:

$$\begin{array}{c} IoU=\frac{Target\cap Prediction}{Target\cup Prediction} \end{array}$$ (8)

For each segmented category, obtain the IoU value through formula (8), and then add and average the IoU values of 21 categories to obtain the average intersection to union ratio, i.e., mIoU.

### Component-wise ablation study

In this section, to reveal the effect of each component in the proposed method, we will discuss our approach from following components in detail: the global domain adaptation attention, the STEM block and the data-dependent regulator.

#### Ablation study for global domain adaptation attention

To capture different types of contextual dependencies for better scene segmentation, we explore two global relations features to derive the global domain adaptation attention. To validate the effect of two global relations on the performance of global domain adaptation attention, we conduct experiments with Cityscapes validation dataset as shown in Table 1, where the **GSR** and **GCR** represent the global spatial relation and global channel relation, respectively. Compared with the baseline FCN (ResNet-50), employing global spatial relations to derive the global domain adaptation attention, thus yielding a result of 76.23% in Mean IoU and bringing 6.2% improvement. Meanwhile, employing global channel relations individually outperforms the baseline by 6.65%. When we integrate two global relations features to generate the attention, the performance further improves to 77.55%. Furthermore, when we adopt a deeper pretrained backbone (ResNet-101), the method with the global domain adaptation attention significantly improves the segmentation performance over the baseline model by 6.81%. Results show that global domain adaptation attention significantly improves the scene segmentation by modeling rich contextual dependencies over global scope relations.

**Table 1:** Effect on global domain adaptation attention.

Method	Backbone	GSR	GCR	mIoU (**%**)
Dilated FCN	ResNet-50	✗	✗	70.03
GDAAR	ResNet-50	✓	✗	76.23
GDAAR	ResNet-50	✗	✓	76.68
GDAAR	ResNet-50	✓	✓	77.55
Dilated FCN	ResNet-101	✗	✗	72.54
GDAAR	ResNet-101	✓	✗	77.81
GDAAR	ResNet-101	✗	✓	78.41
GDAAR	ResNet-101	✓	✓	79.35

**Fig 3:**
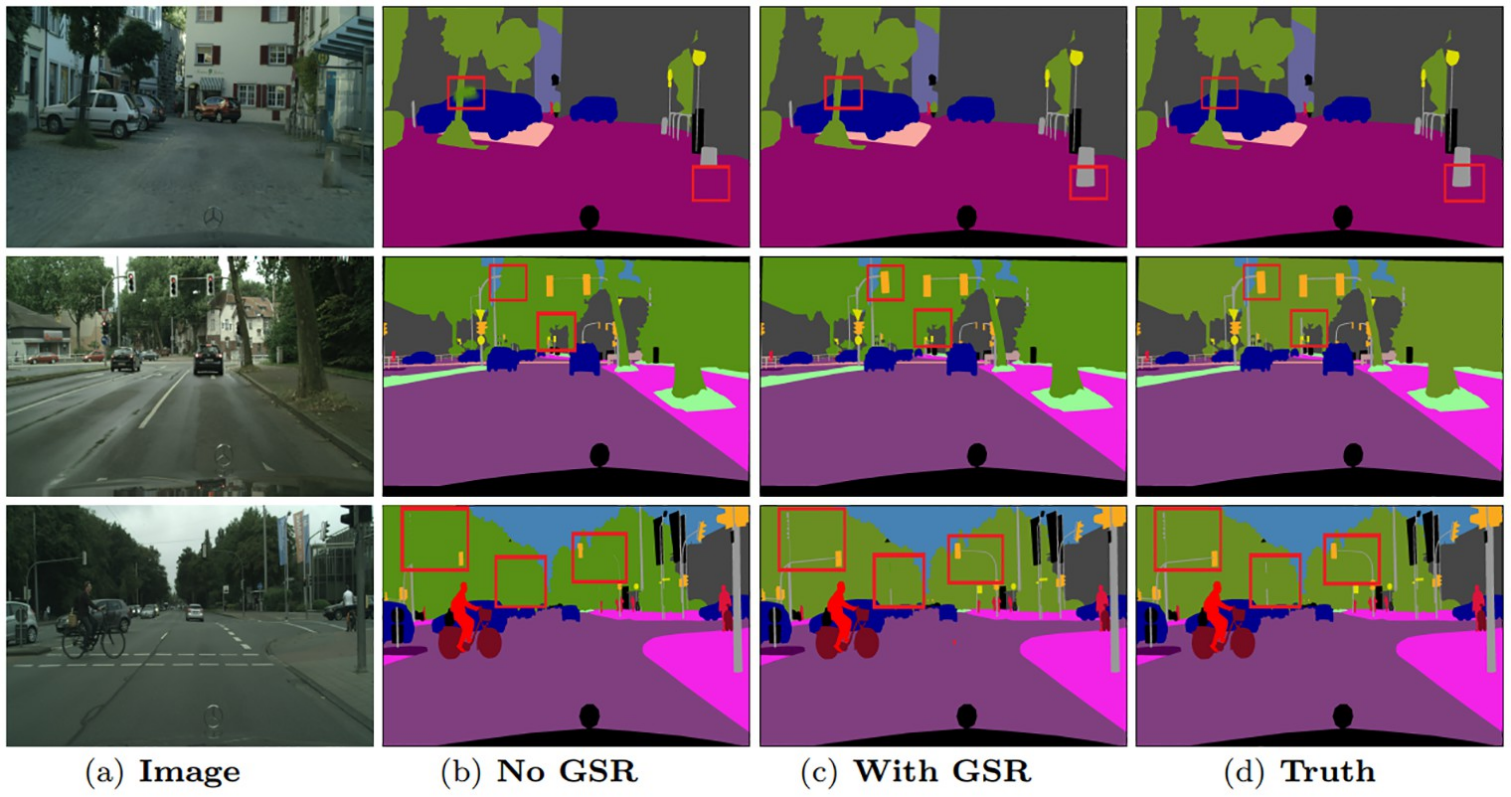
Visualization results of global spatial relation on Cityscapes validation set.

**Fig 4:**
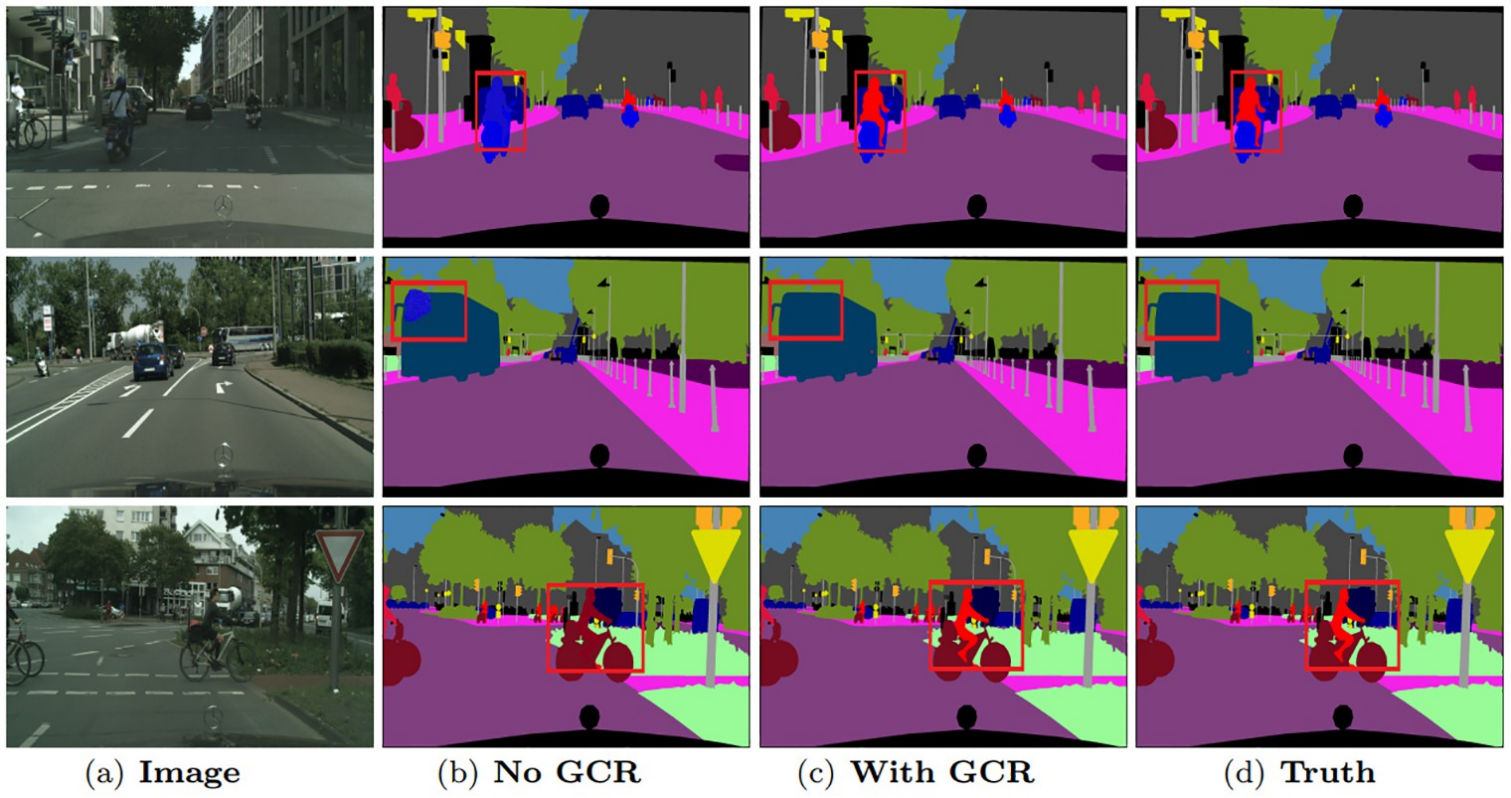
Visualization results of global channel relation on Cityscapes validation set.

The effects of global spatial relations in global domain adaptation attention can be visualized in Fig 3. Some details and object boundaries are clearer with global spatial relations, such as the ‘trunk’ in the first row and the ‘lamppost’ in the third row. The selective integration among global spatial relations enhances the discrimination of details. Meanwhile, Fig 4 demonstrates that, with our global channel relations, some misclassified categories are now correctly classified, such as the ‘person’ in the first and third row. The selective integration among global channel relations helps to capture contextual dependencies. The semantic consistency has been improved obviously.

#### Ablation study for both STEM block and data-dependent regulator

We employ a fixed 3-layer ‘STEM’ block to replace the beginning of the backbone ResNet-101, thus reducing the resolution to 1/4 scale. Moreover, we design a data-dependent regulator to adjust the attention weight on the feature map. To validate the performance on both ‘STEM’ block and data-dependent regulator, we conduct experiments with Cityscapes validation set as shown in Table 2. Compared with employing the pure backbone ResNet-101 in our GDAAR method, employing a fixed 3-layer ‘STEM’ block obtains a Mean IoU of 80.19% and brings 0.85% improvement. Furthermore, when we adopt a data-dependent regulator to adjust the global domain adaptation attention, which significantly improves the segmentation performance by 1.7%. Our experimental results demonstrate that the use of global domain adaptation attention, combined with a data-dependent regulator, enables our model to achieve a wide contextual view and selectively aggregate contexts. Through this process, feature representations are able to achieve mutual gains and are more robust for scene segmentation. By leveraging global contextual information, our model is able to better capture the complex relationships between different objects and materials within a scene, leading to improved segmentation accuracy and overall performance. Moreover, the use of a data-dependent regulator allows our model to adapt to different input domains and improve generalization, making it more suitable for real-world applications. These findings highlight the effectiveness and versatility of our approach for semantic segmentation tasks in various settings.

**Table 2:** Performance on both STEM block and regulator.

Method	GDAA	STEM	Regulator	mIoU (**%**)
GDAAR-101	✓	✗	✗	79.34
GDAAR-101	✓	✓	✗	80.19
GDAAR-101	✓	✗	✓	81.04
GDAAR-101	✓	✓	✓	83.34

**Fig 5:**
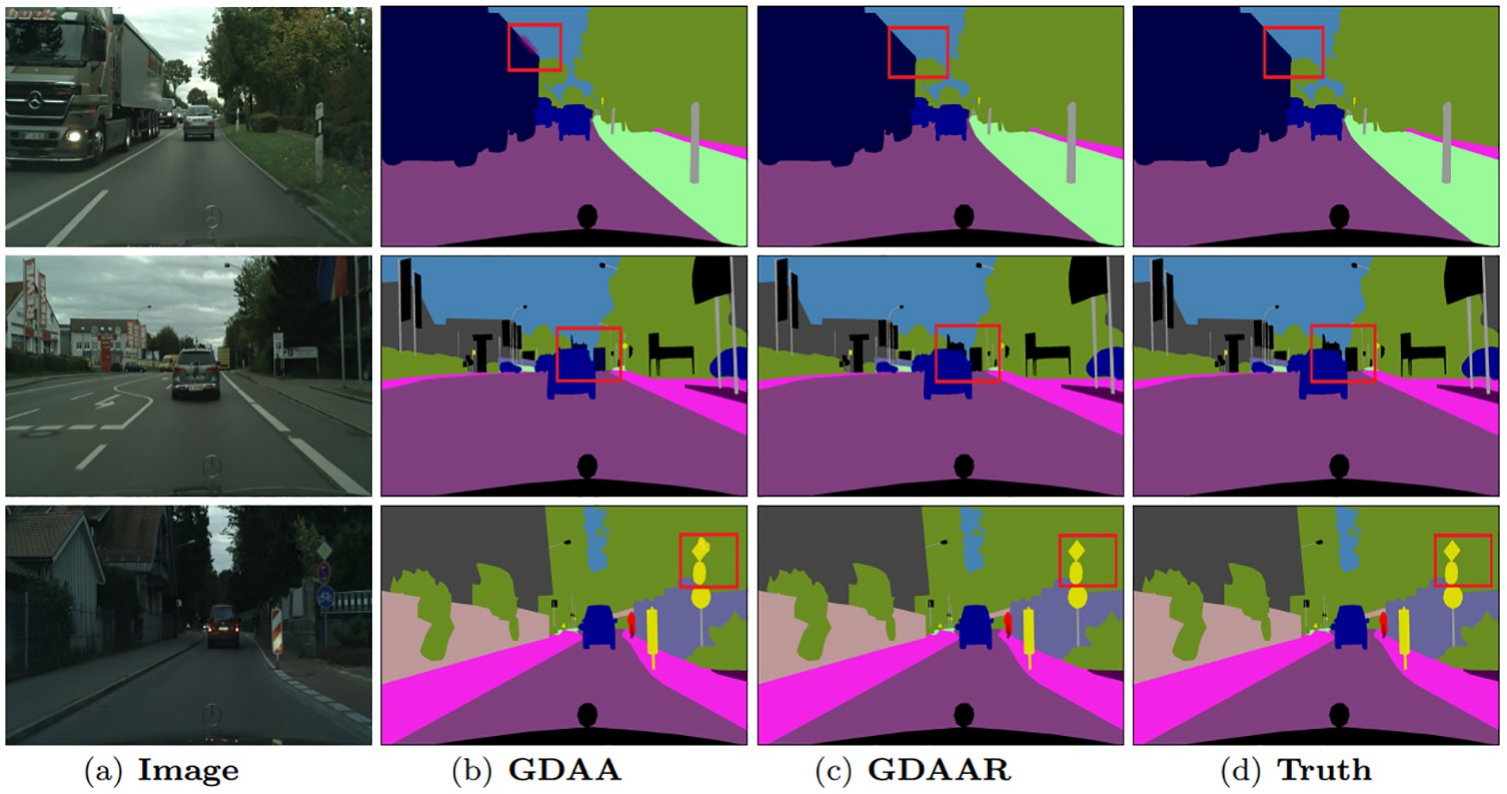
Visualization results of our GDAAR method on Cityscapes validation set.

The effects of the data-dependent regulator in global domain adaptation attention can be visualized in Fig 5. It significantly improves the features similarity belonging to the same segment region while keeping the discriminative power of features belonging to different segments, such as the ‘bus’ in the first row and the ‘guideboard’ in the third row. The data-dependent regulator collecting contextual information in horizontal and vertical directions enhances pixel-wise representation capability, which further remarkably improves the semantic consistency.

### Comparisons on Cityscapes dataset

To validate the effectiveness of our approach, we compare it with existing methods on the Cityscapes testing set. As shown in Table 3, our proposed method, GDAAR, outperforms state-of-the-art approaches. Specifically, our model achieves higher accuracy than DANet [38], which also uses ResNet-101 as the backbone architecture. This improvement is primarily due to our use of global domain adaptation attention, which allows for a wider contextual view and selective context aggregation, while DANet uses dual attention on local features to explore contextual dependencies. Furthermore, our approach outperforms DenseASPP [2] by a significant margin, despite the fact that DenseASPP employs more powerful pre-trained models than ours. These results demonstrate the superior performance of our approach in capturing complex scene contexts and improving segmentation accuracy over existing state-of-the-art methods.

**Table 3:** Segmentation comparisons on cityscapes dataset.

Method	Backbone	mIoU (**%**)
BiSeNet [44]	ResNet-101	78.9
AAF [45]	ResNet-101	79.1
DFN [10]	ResNet-101	79.3
PSANet [5]	ResNet-101	80.1
DenseASPP [2]	DenseNet-161	80.6
ANL [46]	ResNet-101	81.3
CCNet [39]	ResNet-101	81.4
DANet [38]	ResNet-101	81.5
CPNet [47]	ResNet-101	81.3
FPN [48]	ResNet-50	76.4
Ince-DResAsppNet [49]	DenseNet	78.1
SFSM [50]	ResNet-101	81.5
**Ours (GDAAR)**	**ResNet-101**	**82.1**

### Comparisons on PASCAL context dataset

To further evaluate the effectiveness of our approach, we conduct experiments on the PASCAL Context dataset. As shown in Table 4, with a deep pre-trained network ResNet-101, our method achieves a Mean IoU of 55.4%, outperforming previous state-of-the-art methods by a large margin. Among these methods, RefineNet [15] utilizes both multi-scale feature fusion and a deeper model (ResNet-152) to improve its segmentation results. In contrast, our approach introduces global domain adaptation attention with data-dependent regulator to capture rich contextual dependencies over global scope relations, leading to significantly improve segmentation results. Our approach is able to effectively leverage global context information and selectively aggregate features, resulting in more robust and accurate segmentation performance. These results demonstrate the superiority of our approach compared to existing state-of-the-art methods on the challenging PASCAL Context dataset.

**Table 4:** Segmentation comparisons on PASCAL context.

Method	Backbone	mIoU (%)
RefineNet [15]	ResNet-152	47.3
PSPNet [4]	ResNet-101	47.8
CCL [14]	ResNet-101	51.6
EncNet [11]	ResNet-101	51.7
DANet [38]	ResNet-101	52.6
ANL [46]	ResNet-101	52.8
CPNet [47]	ResNet-101	53.9
**Ours (GDAAR)**	**ResNet-101**	**55.4**

### Comparisons on COCO stuff dataset

To evaluate the generalization of our proposed approach, we also conduct experiments on the COCO Stuff dataset. As shown in Table 5, our method achieves a Mean IoU score of 45.4%, outperforming existing state-of-the-art methods by a large margin. Among the compared methods, DAG-RNN [21] utilizes chain-RNNs to model rich spatial dependencies, while CCL [14] adopts a gating mechanism in the decoder stage to improve segmentation accuracy for inconspicuous objects and background stuff. In contrast, our method designs a global domain adaptation attention with data-dependent regulator, which significantly improves feature similarity within the same segment region while preserving discriminative power for features belonging to different segments. By effectively leveraging contextual information and selectively aggregating features, our approach demonstrates superior performance in segmenting diverse objects and stuff classes on the COCO Stuff dataset. These results highlight the generalizability and effectiveness of our approach across various datasets and settings.

**Table 5:** Segmentation comparisons on COCO stuff dataset.

Method	Backbone	mIoU (%)
DeepLab-v2 [26]	ResNet-101	26.9
DAG-RNN [21]	RNN	31.2
RefineNet [15]	ResNet-101	33.6
CCL [14]	ResNet-101	35.7
DANet [38]	ResNet-101	39.7
**Ours (GDAAR)**	**ResNet-101**	**45.4**

## Conclusion

For scene segmentation, in order to explore rich contextual dependencies, we present a **G**lobal **D**omain **A**daptation **A**ttention with Data-Dependent **R**egulator (GDAAR) method that models the global scope distribution information and based on this to infer attention. Particularly, we represent the global scope relations by aggregating the spatial (GSR) and channel relations (GCR) and based on them to derive the global domain adaptation attention, thus better distinguishing different types of contextual dependencies from a global view. Moreover, a data-dependent regulator is designed to adjust the attention weight on the feature map, which significantly improves the features similarity belonging to the same segment region while keeping the discriminative power of features belonging to different segments. We conduct extensive ablation studies to demonstrate the superiority of proposed GDAAR in modeling rich contextual dependencies, and validate the state-of-the-art performance of our method on the benchmark datasets.

Our method is based on fully supervised learning and requires pixel-wise annotations, which can be time-consuming and expensive to obtain. To alleviate the burden of pixel-wise annotations, in future work, we will focus on weakly supervised learning for semantic segmentation, attempting to achieve equivalent segmentation performance of fully supervised approaches.

## Supporting information

S1 Data (ZIP)
