# Supplementary figures and images for: Global domain adaptation attention with data-dependent regulator for scene segmentation
Source: PLoS One. 2024 Feb 14;19(2):e0295263. doi: 10.1371/journal.pone.0295263 (PMC10866527; doi:10.1371/journal.pone.0295263)

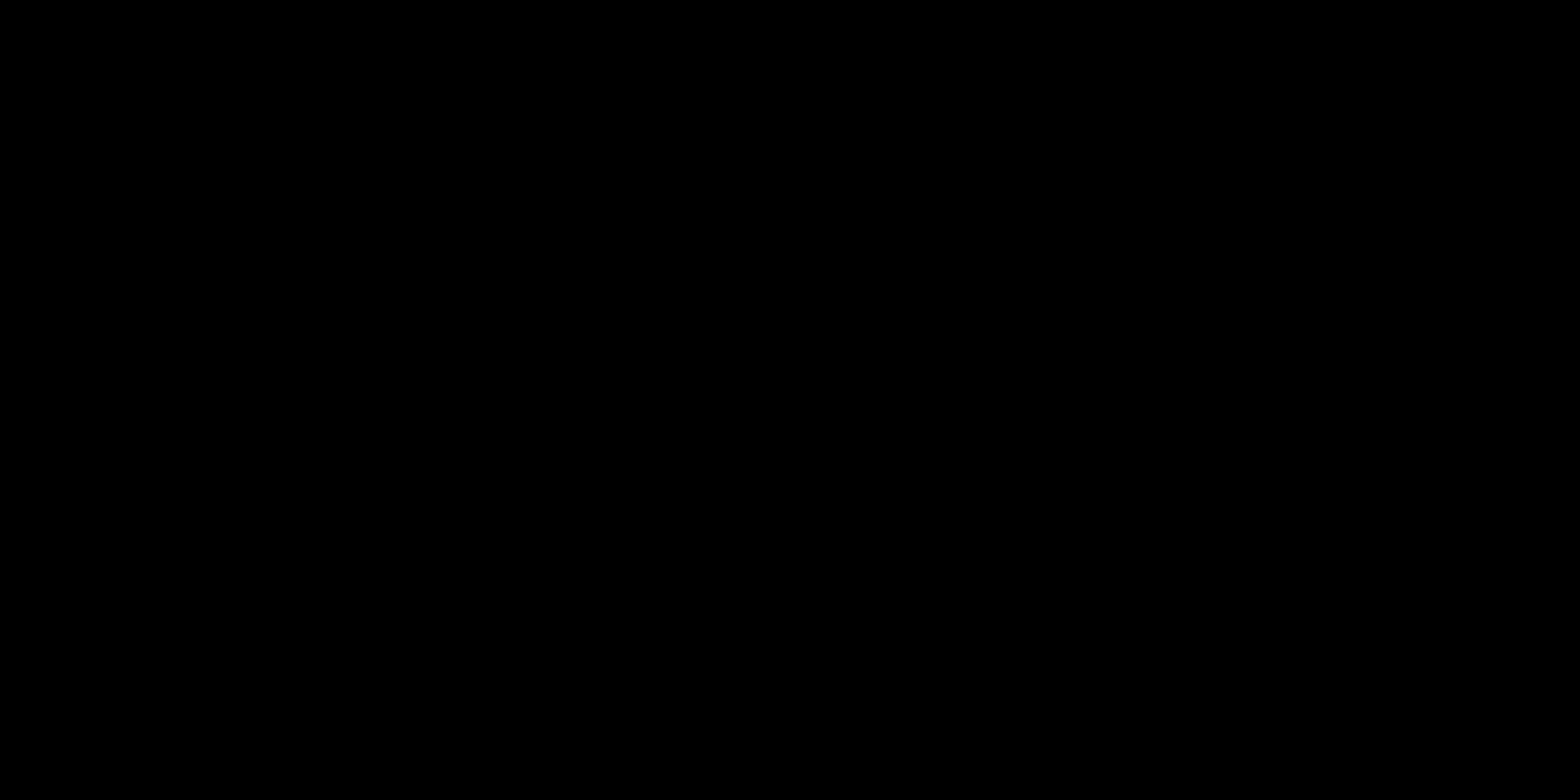

Supplement: S1 Data — (ZIP) [file pone.0295263.s001.zip › ╨┬╜¿╬─╝■╝╨ (2)/groundtruth/berlin_000000_000019_gtFine_color.png]

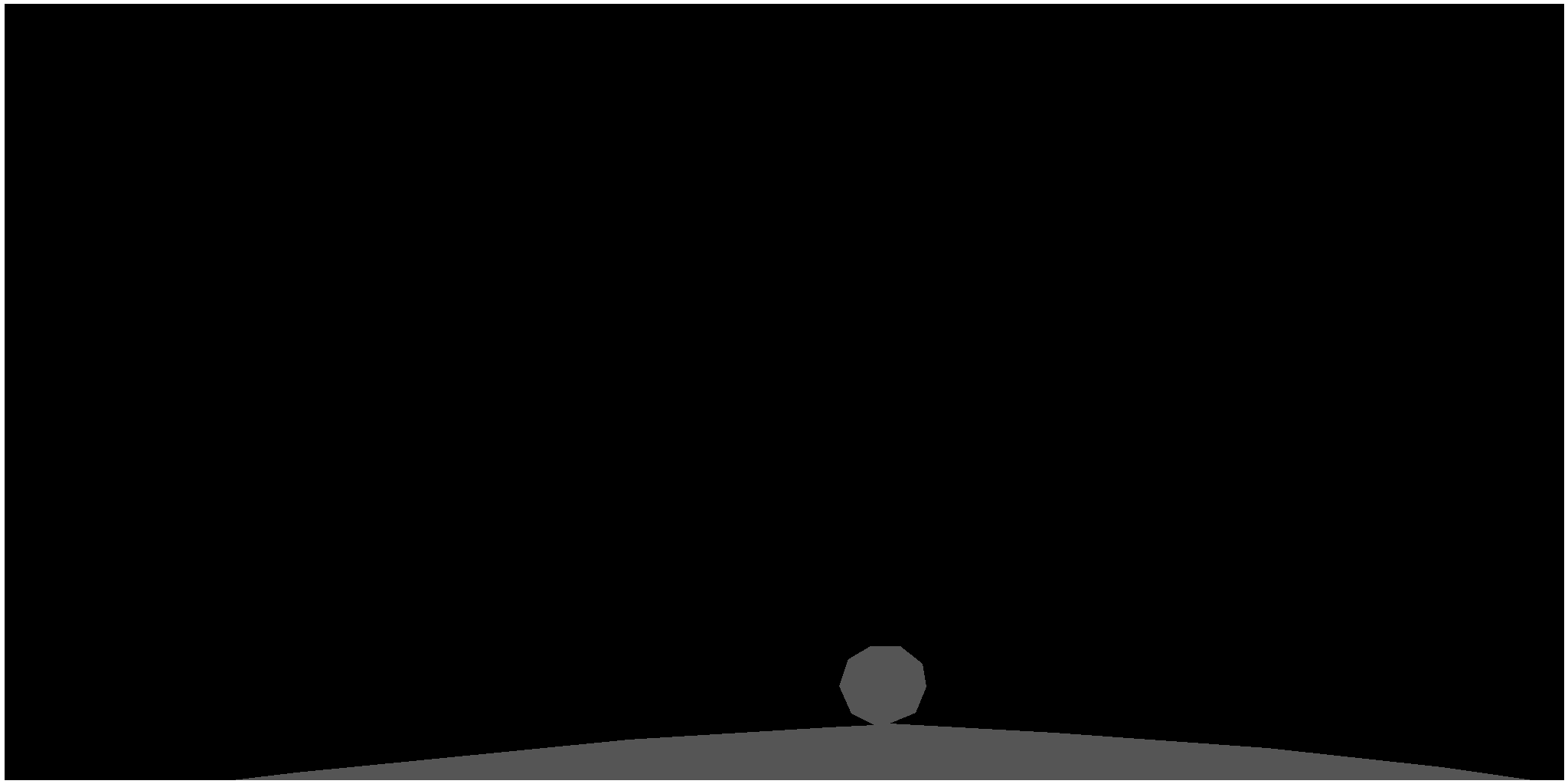

Supplement: S1 Data — (ZIP) [file pone.0295263.s001.zip › ╨┬╜¿╬─╝■╝╨ (2)/groundtruth/berlin_000000_000019_gtFine_instanceIds.png]

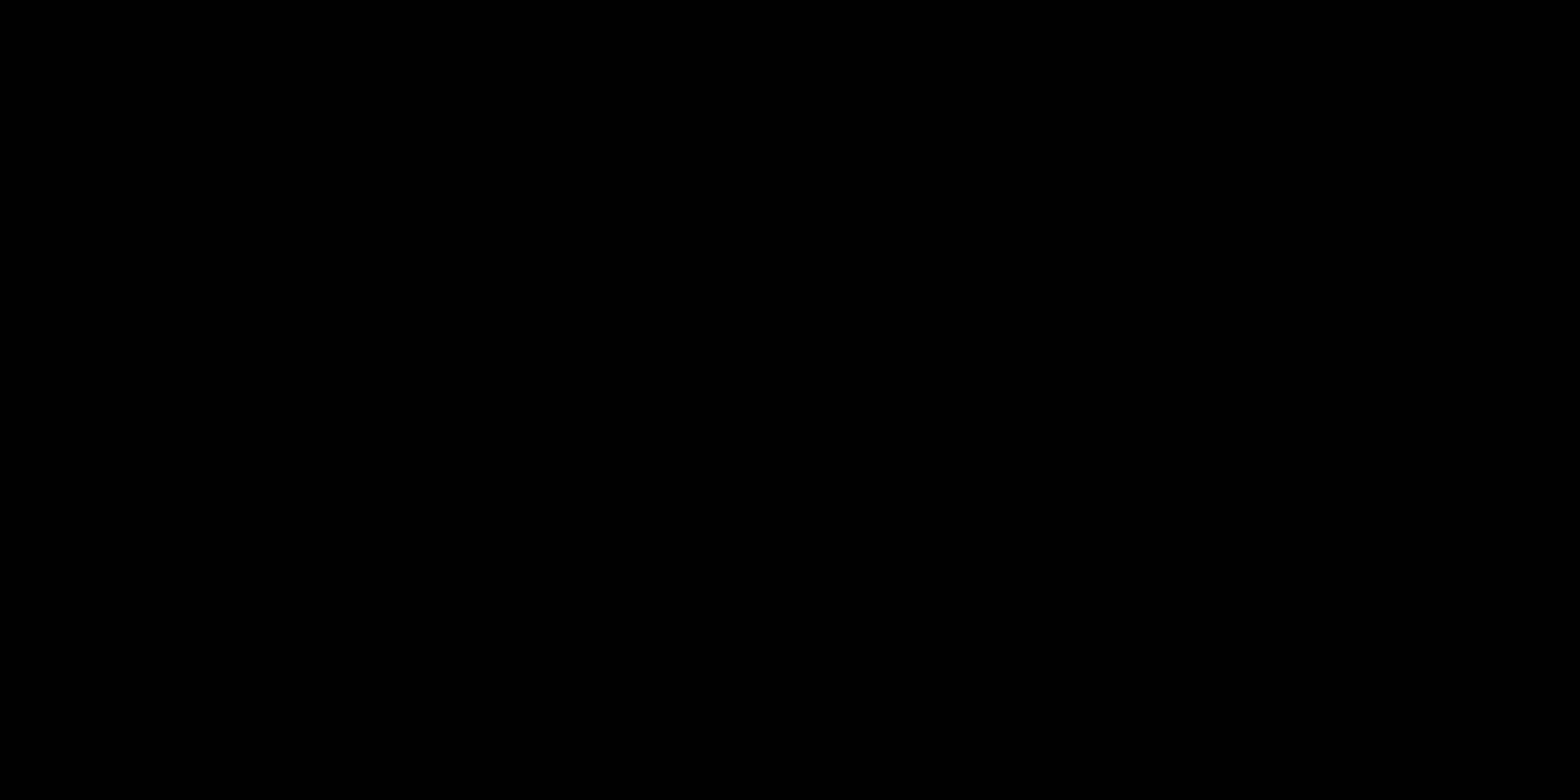

Supplement: S1 Data — (ZIP) [file pone.0295263.s001.zip › ╨┬╜¿╬─╝■╝╨ (2)/groundtruth/berlin_000000_000019_gtFine_instanceTrainIds.png]

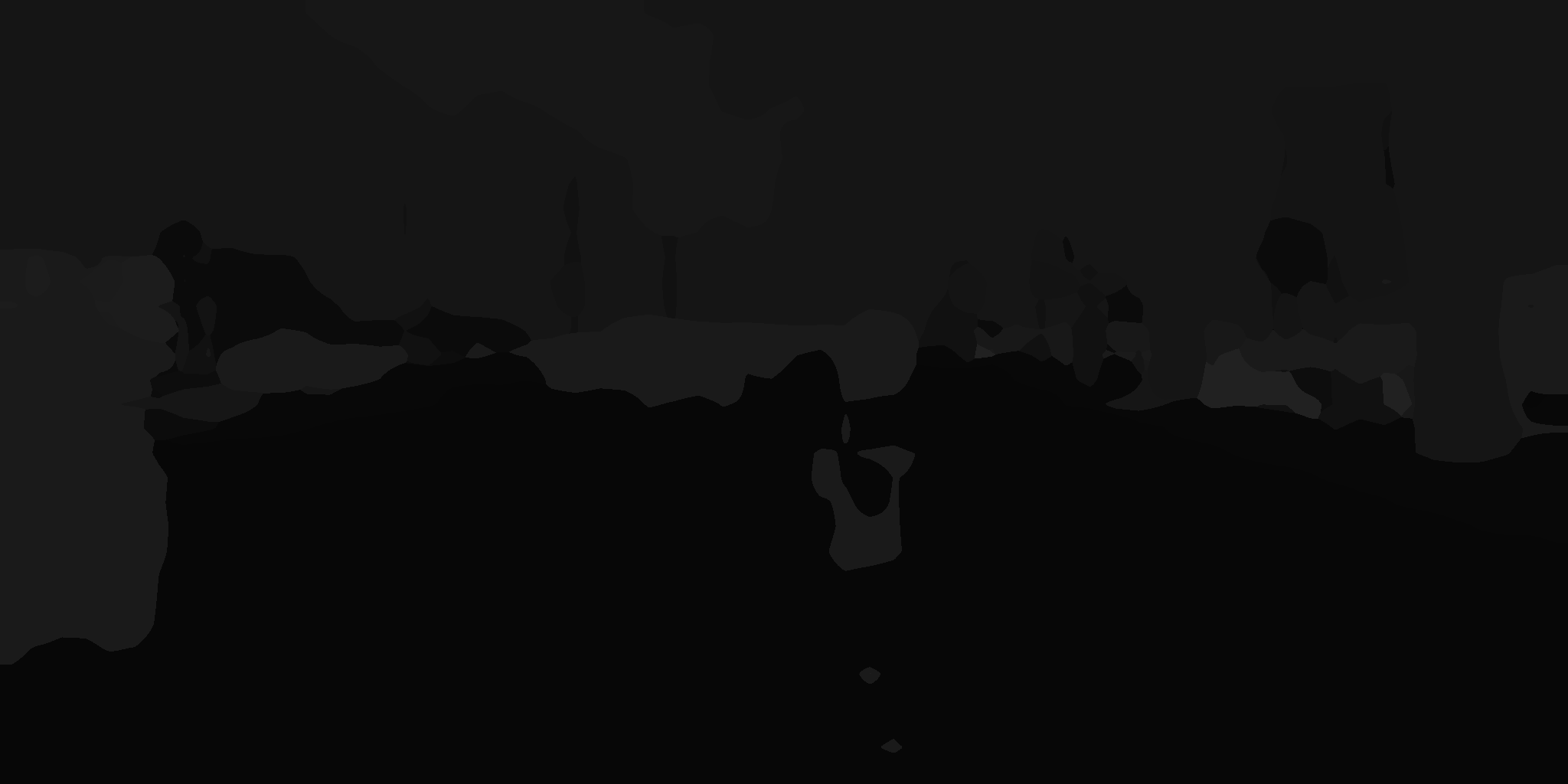

Supplement: S1 Data — (ZIP) [file pone.0295263.s001.zip › ╨┬╜¿╬─╝■╝╨ (2)/groundtruth/berlin_000000_000019_gtFine_labelIds.png]

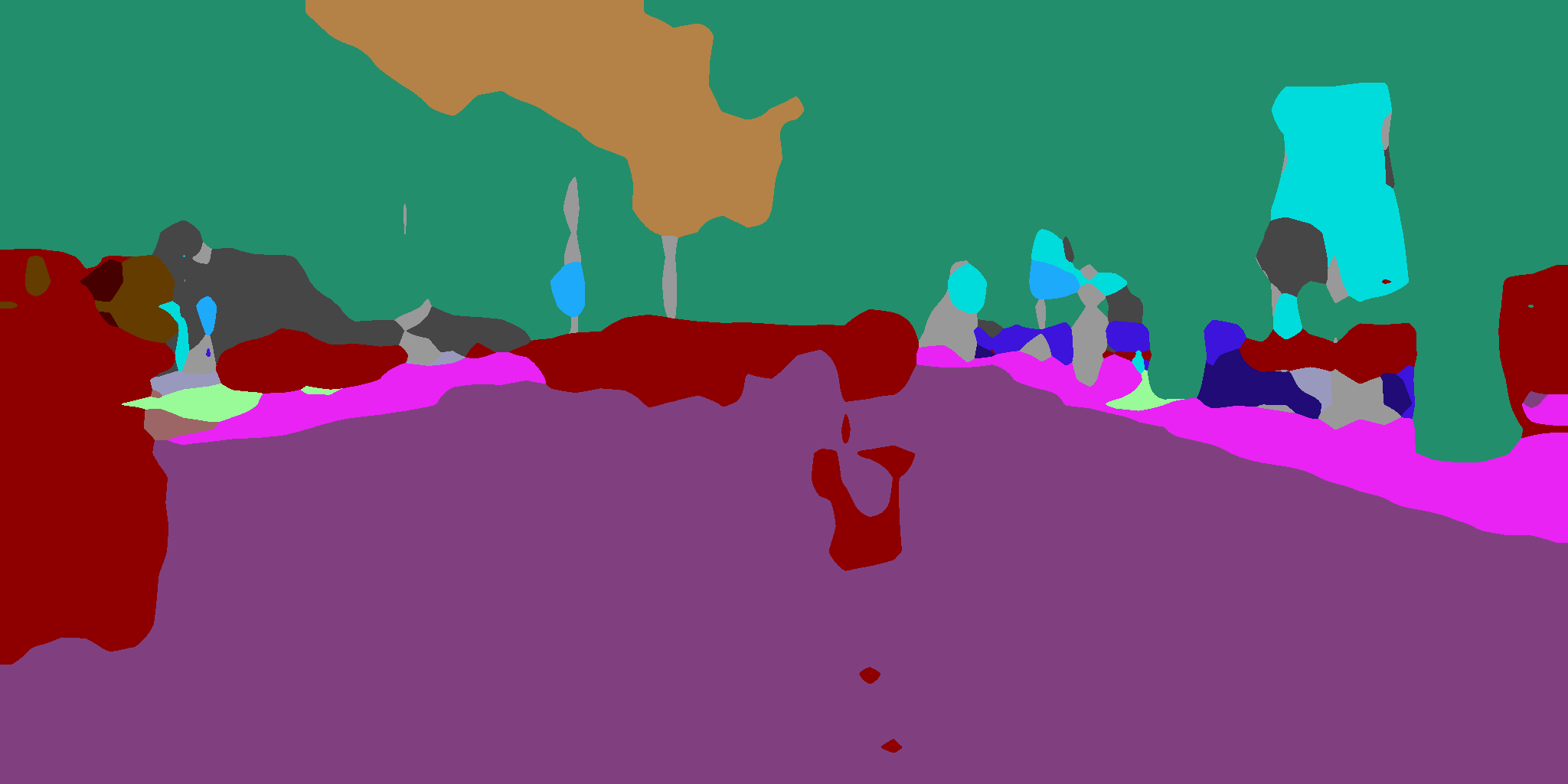

Supplement: S1 Data — (ZIP) [file pone.0295263.s001.zip › ╨┬╜¿╬─╝■╝╨ (2)/groundtruth/berlin_000000_000019_gtFine_labelTrainIds.png]

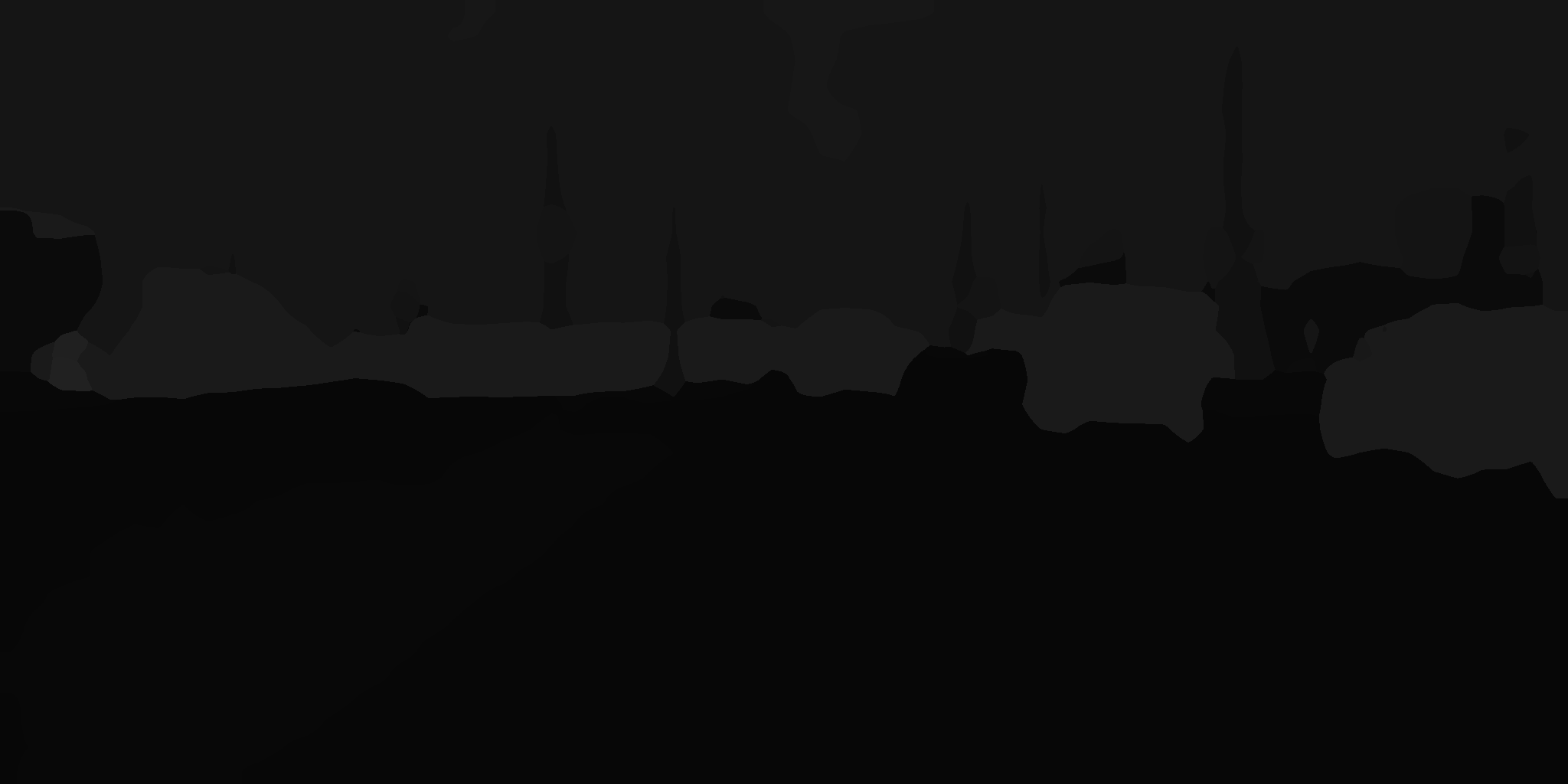

Supplement: S1 Data — (ZIP) [file pone.0295263.s001.zip › ╨┬╜¿╬─╝■╝╨ (2)/groundtruth/berlin_000001_000019_gtFine_labelIds.png]

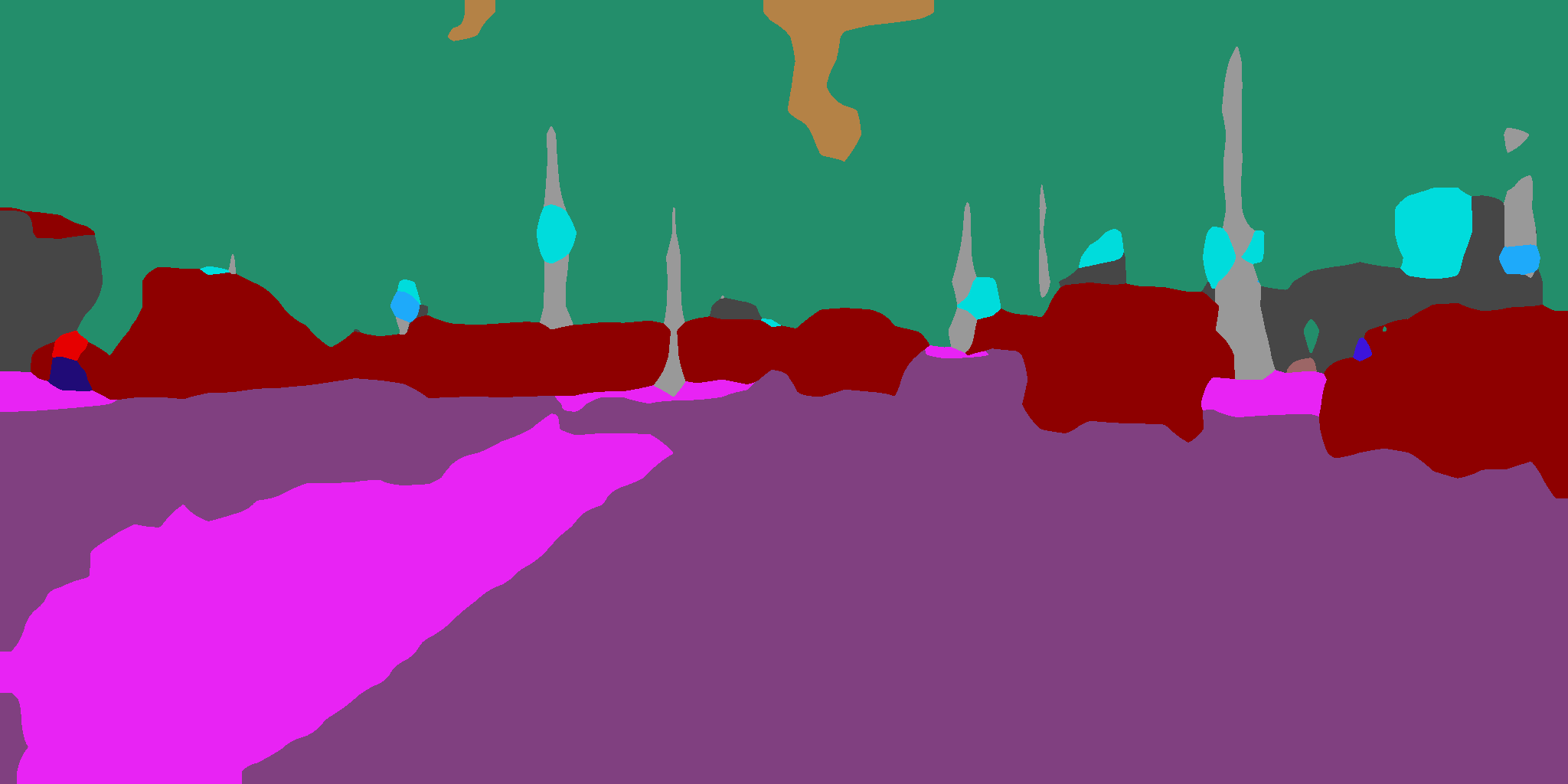

Supplement: S1 Data — (ZIP) [file pone.0295263.s001.zip › ╨┬╜¿╬─╝■╝╨ (2)/groundtruth/berlin_000001_000019_gtFine_labelTrainIds.png]

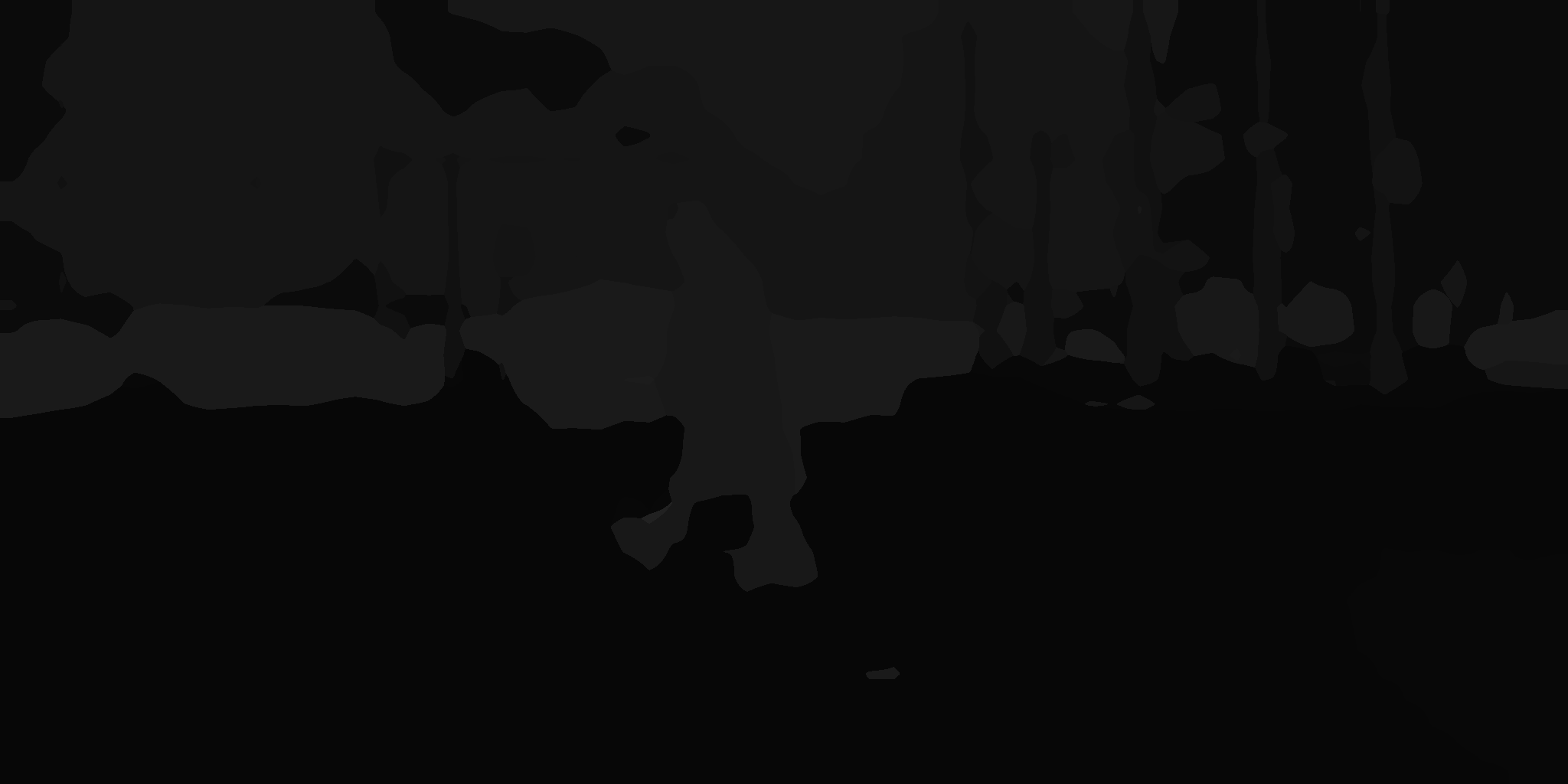

Supplement: S1 Data — (ZIP) [file pone.0295263.s001.zip › ╨┬╜¿╬─╝■╝╨ (2)/groundtruth/berlin_000002_000019_gtFine_labelIds.png]

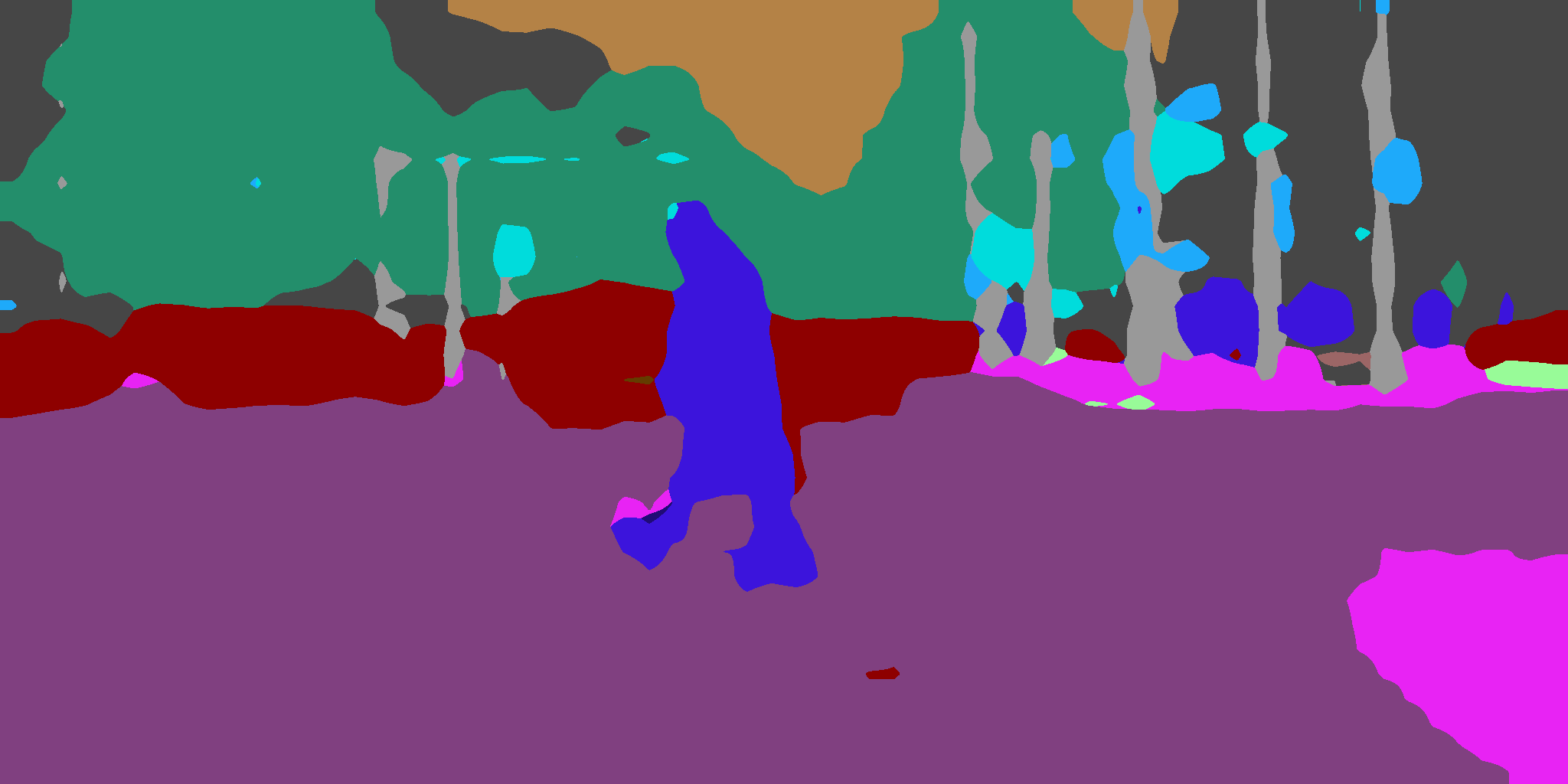

Supplement: S1 Data — (ZIP) [file pone.0295263.s001.zip › ╨┬╜¿╬─╝■╝╨ (2)/groundtruth/berlin_000002_000019_gtFine_labelTrainIds.png]

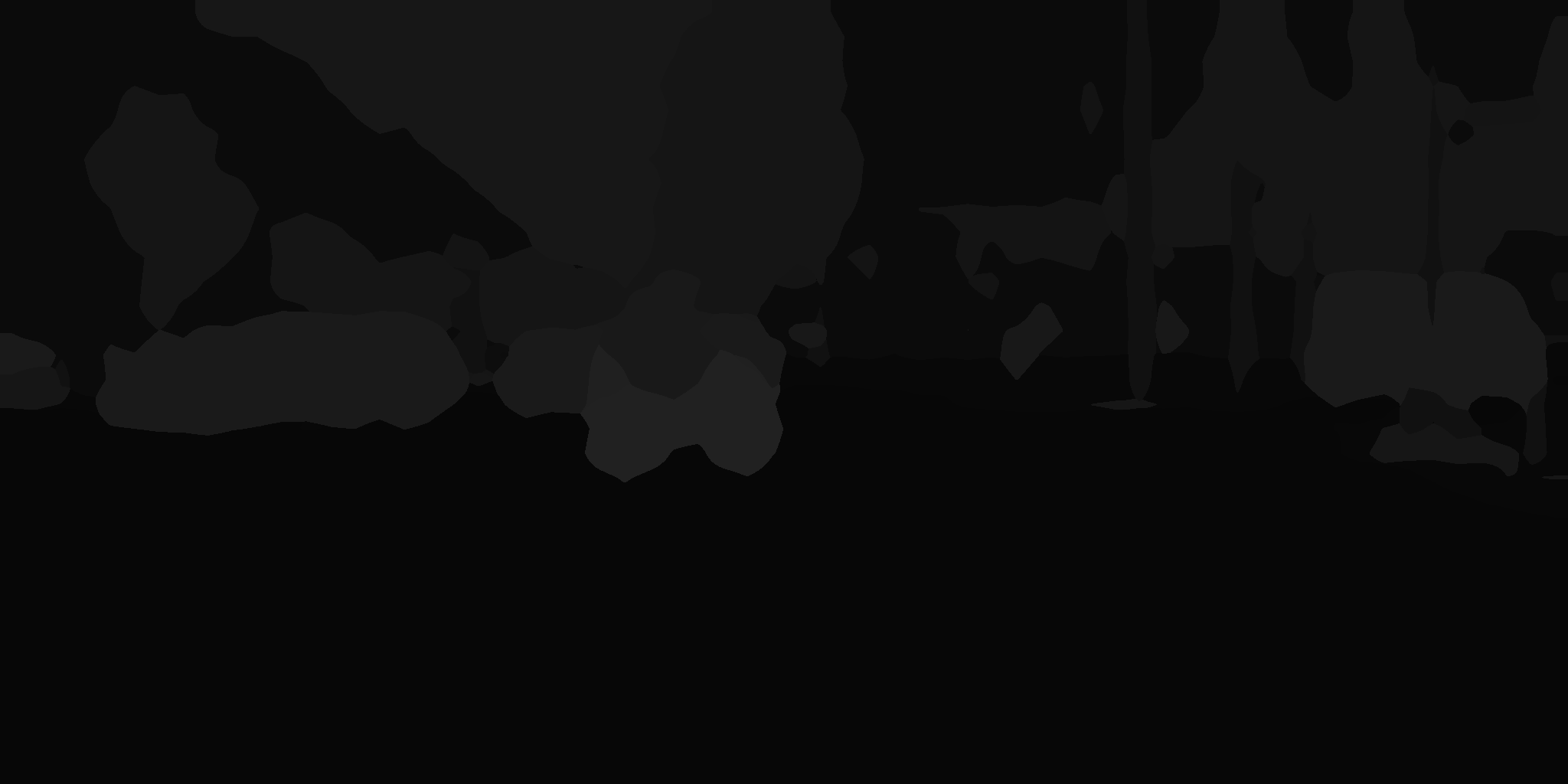

Supplement: S1 Data — (ZIP) [file pone.0295263.s001.zip › ╨┬╜¿╬─╝■╝╨ (2)/groundtruth/berlin_000003_000019_gtFine_labelIds.png]

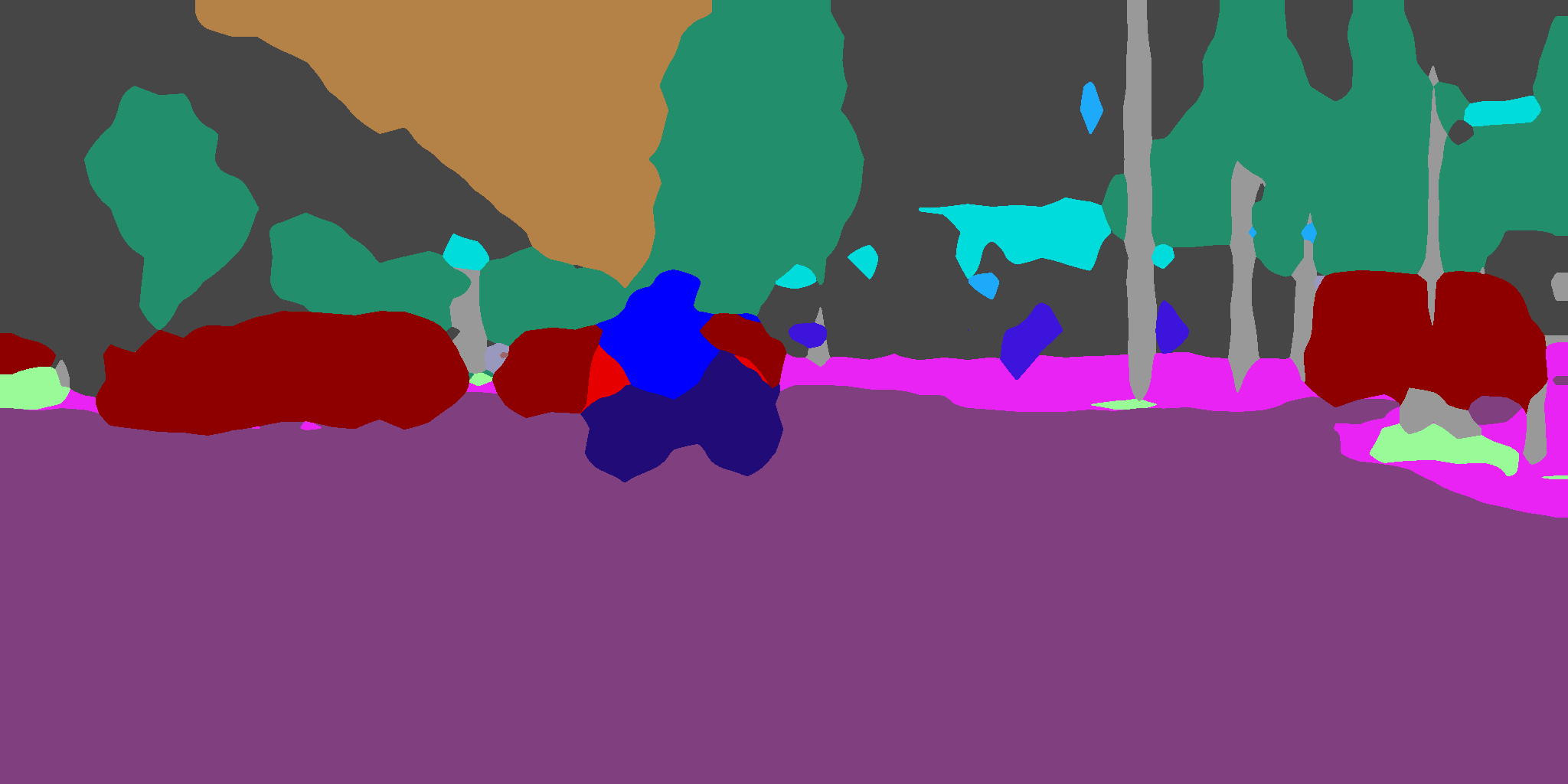

Supplement: S1 Data — (ZIP) [file pone.0295263.s001.zip › ╨┬╜¿╬─╝■╝╨ (2)/groundtruth/berlin_000003_000019_gtFine_labelTrainIds.png]

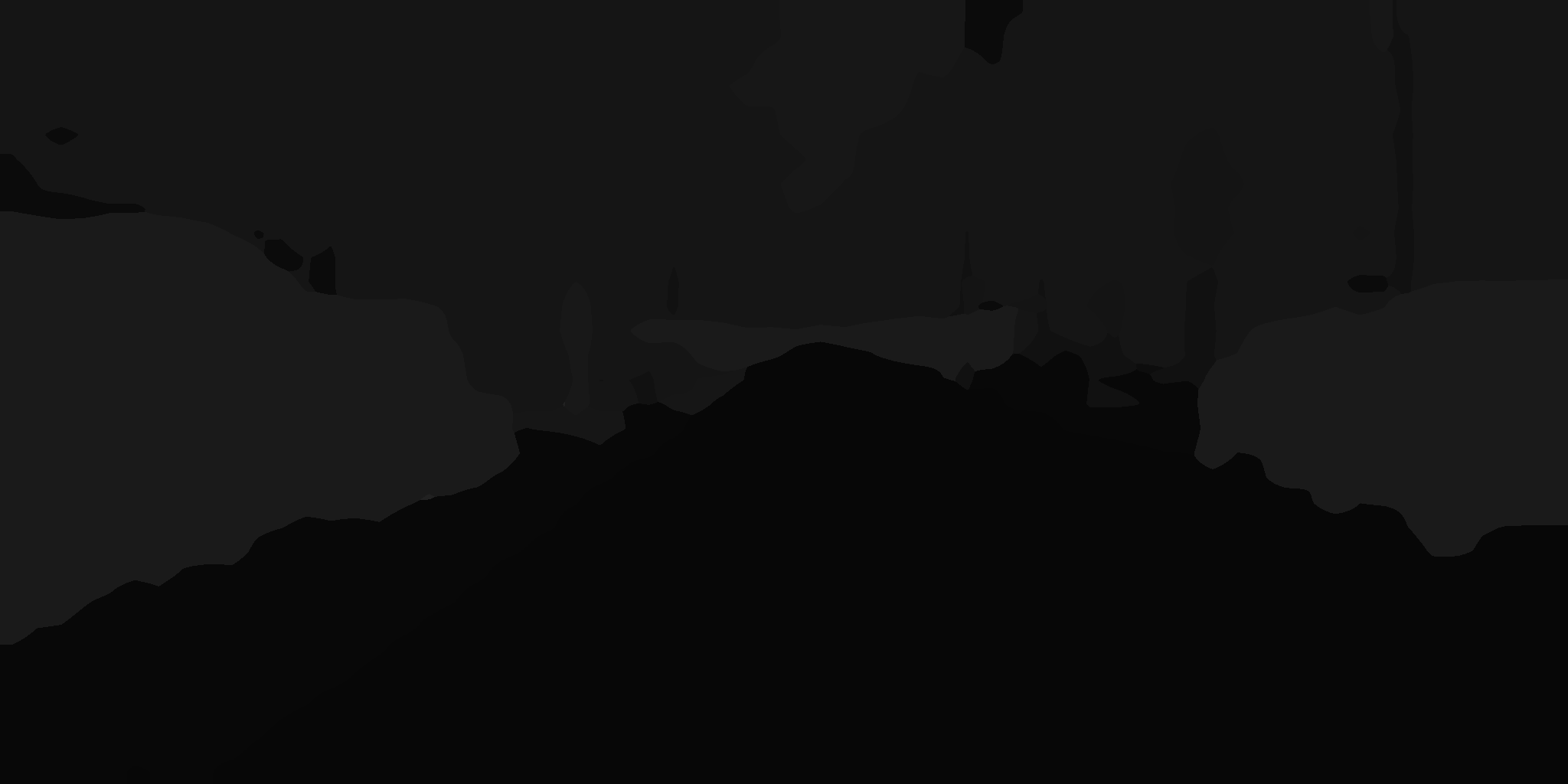

Supplement: S1 Data — (ZIP) [file pone.0295263.s001.zip › ╨┬╜¿╬─╝■╝╨ (2)/groundtruth/berlin_000004_000019_gtFine_labelIds.png]

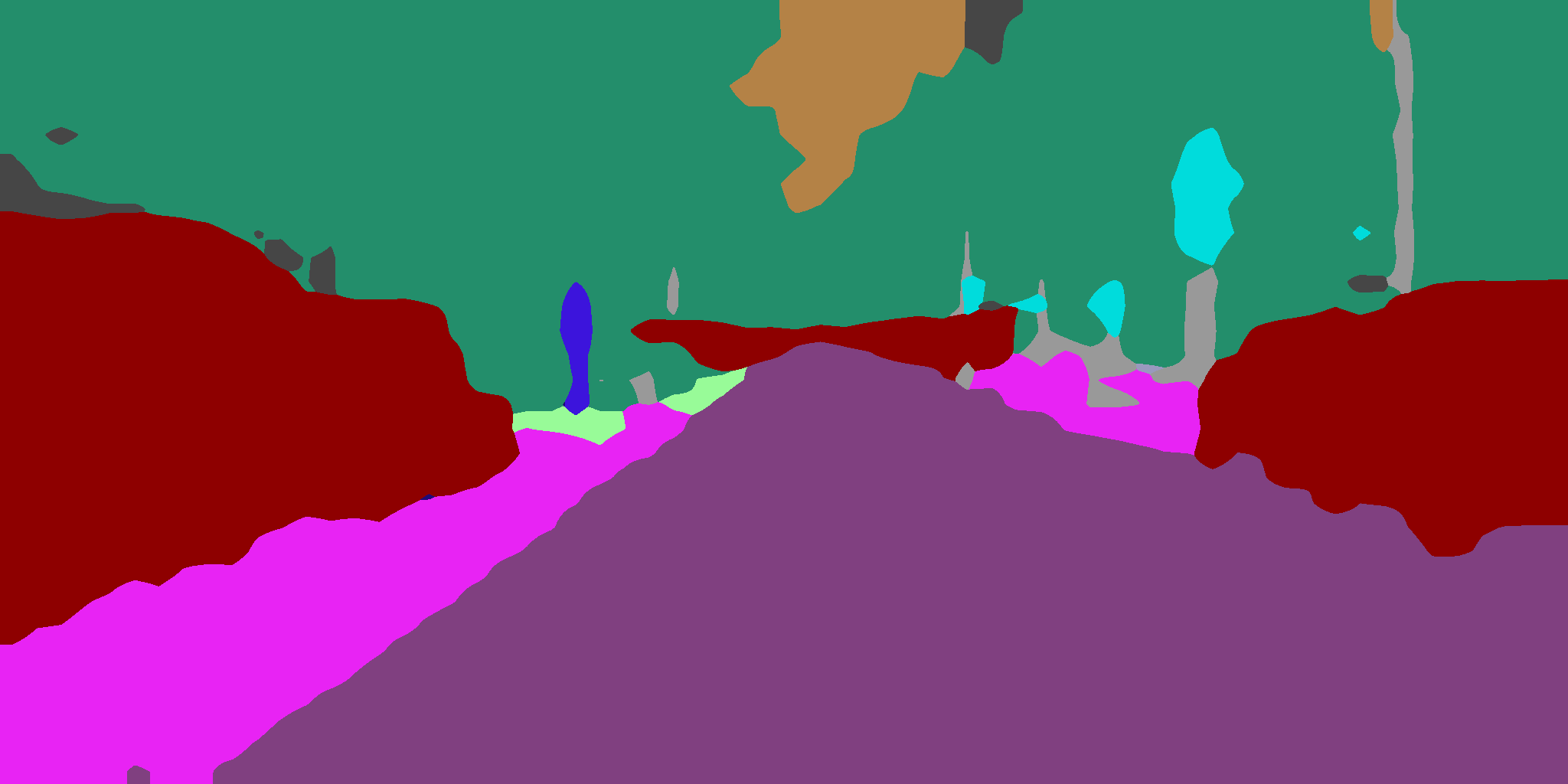

Supplement: S1 Data — (ZIP) [file pone.0295263.s001.zip › ╨┬╜¿╬─╝■╝╨ (2)/groundtruth/berlin_000004_000019_gtFine_labelTrainIds.png]

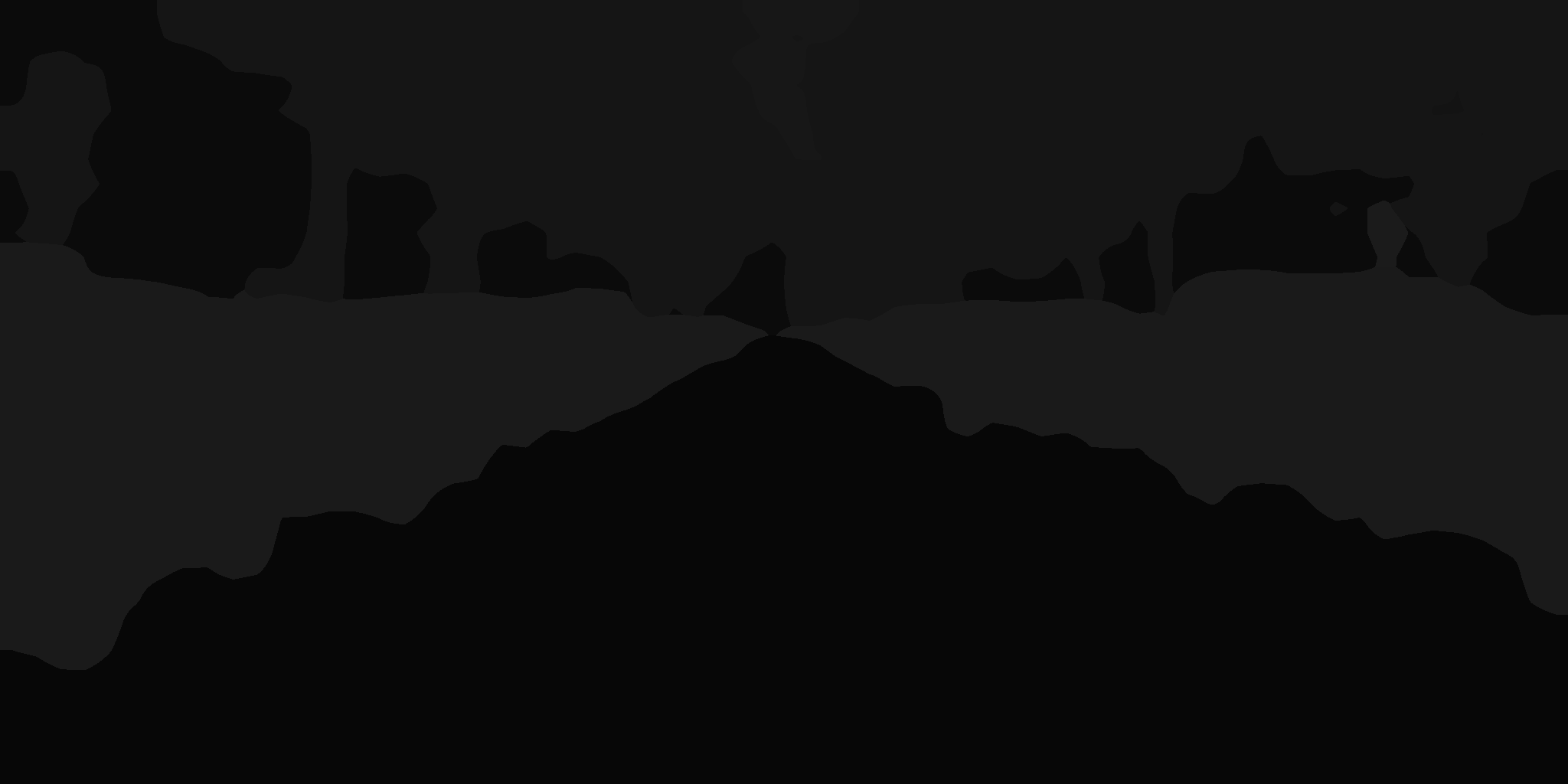

Supplement: S1 Data — (ZIP) [file pone.0295263.s001.zip › ╨┬╜¿╬─╝■╝╨ (2)/groundtruth/berlin_000005_000019_gtFine_labelIds.png]

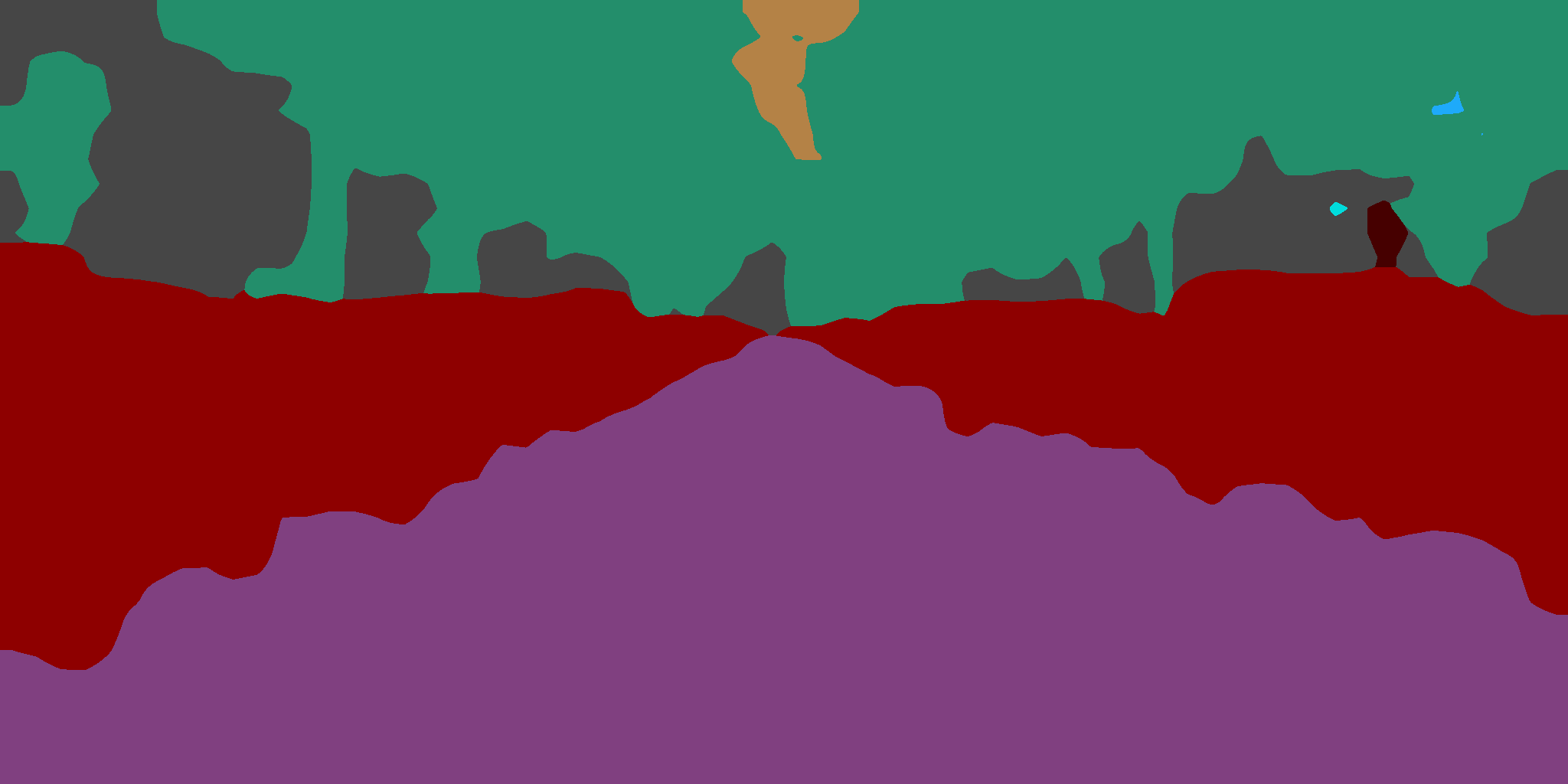

Supplement: S1 Data — (ZIP) [file pone.0295263.s001.zip › ╨┬╜¿╬─╝■╝╨ (2)/groundtruth/berlin_000005_000019_gtFine_labelTrainIds.png]

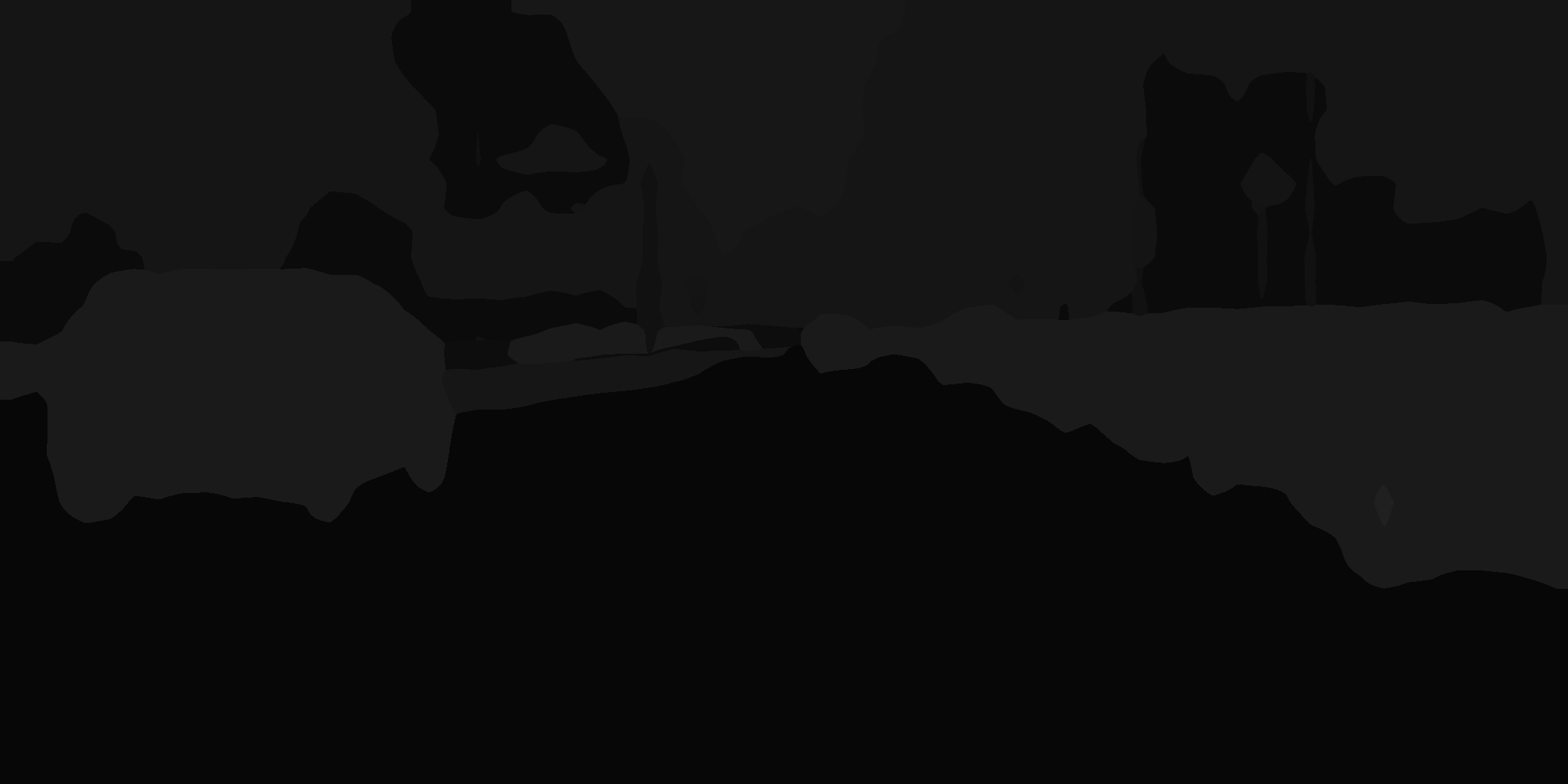

Supplement: S1 Data — (ZIP) [file pone.0295263.s001.zip › ╨┬╜¿╬─╝■╝╨ (2)/groundtruth/berlin_000006_000019_gtFine_labelIds.png]

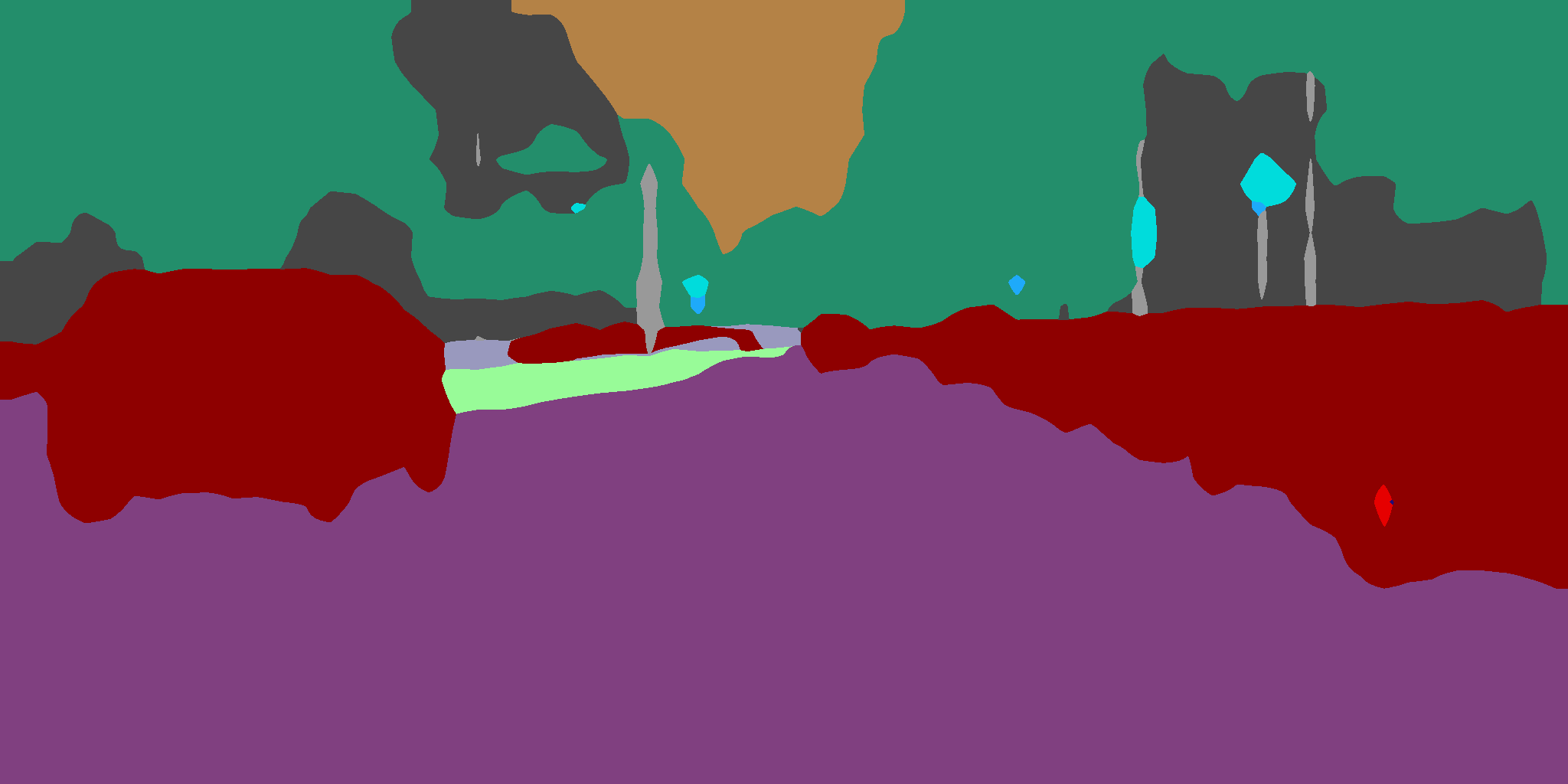

Supplement: S1 Data — (ZIP) [file pone.0295263.s001.zip › ╨┬╜¿╬─╝■╝╨ (2)/groundtruth/berlin_000006_000019_gtFine_labelTrainIds.png]

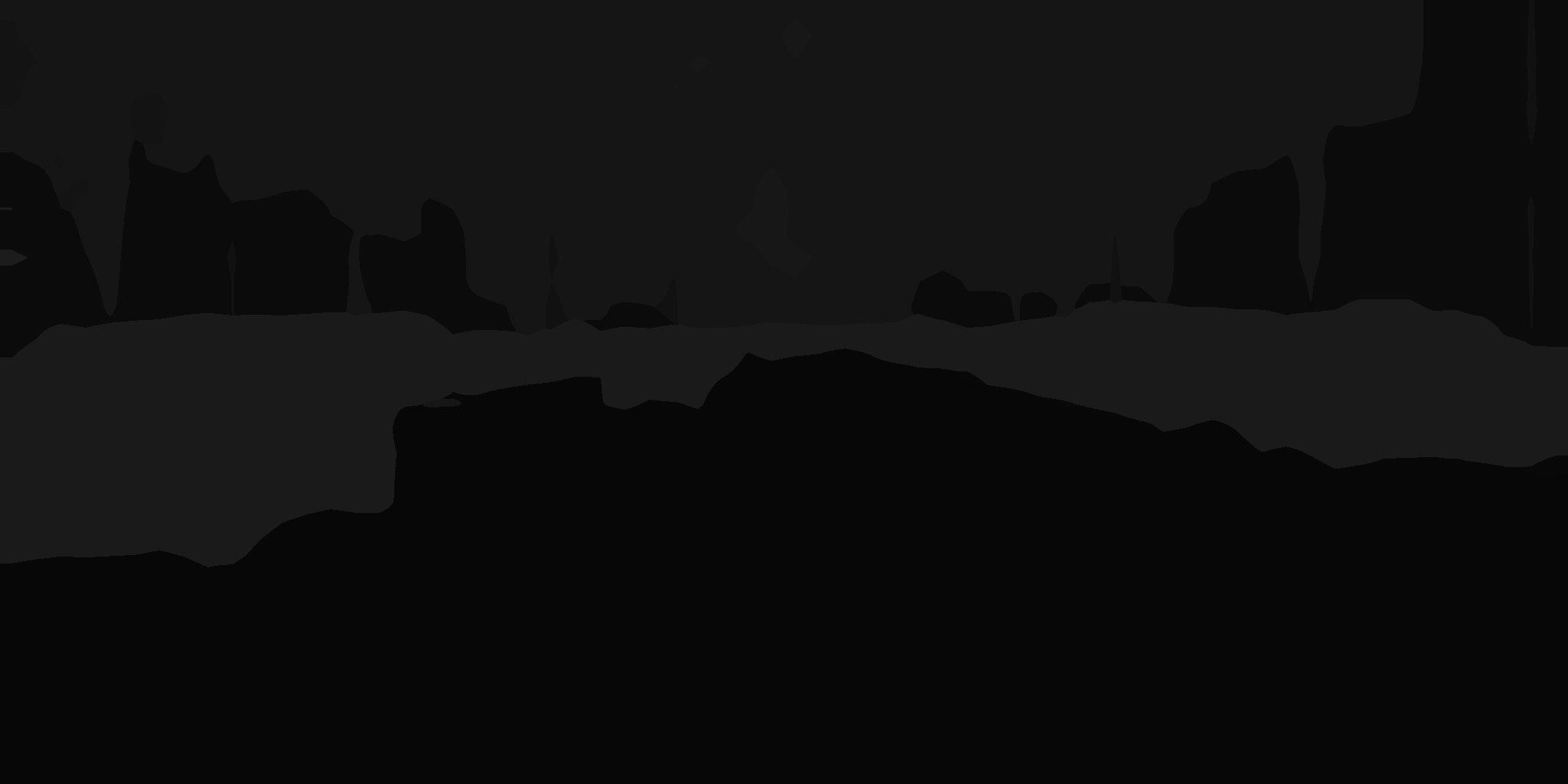

Supplement: S1 Data — (ZIP) [file pone.0295263.s001.zip › ╨┬╜¿╬─╝■╝╨ (2)/groundtruth/berlin_000007_000019_gtFine_labelIds.png]

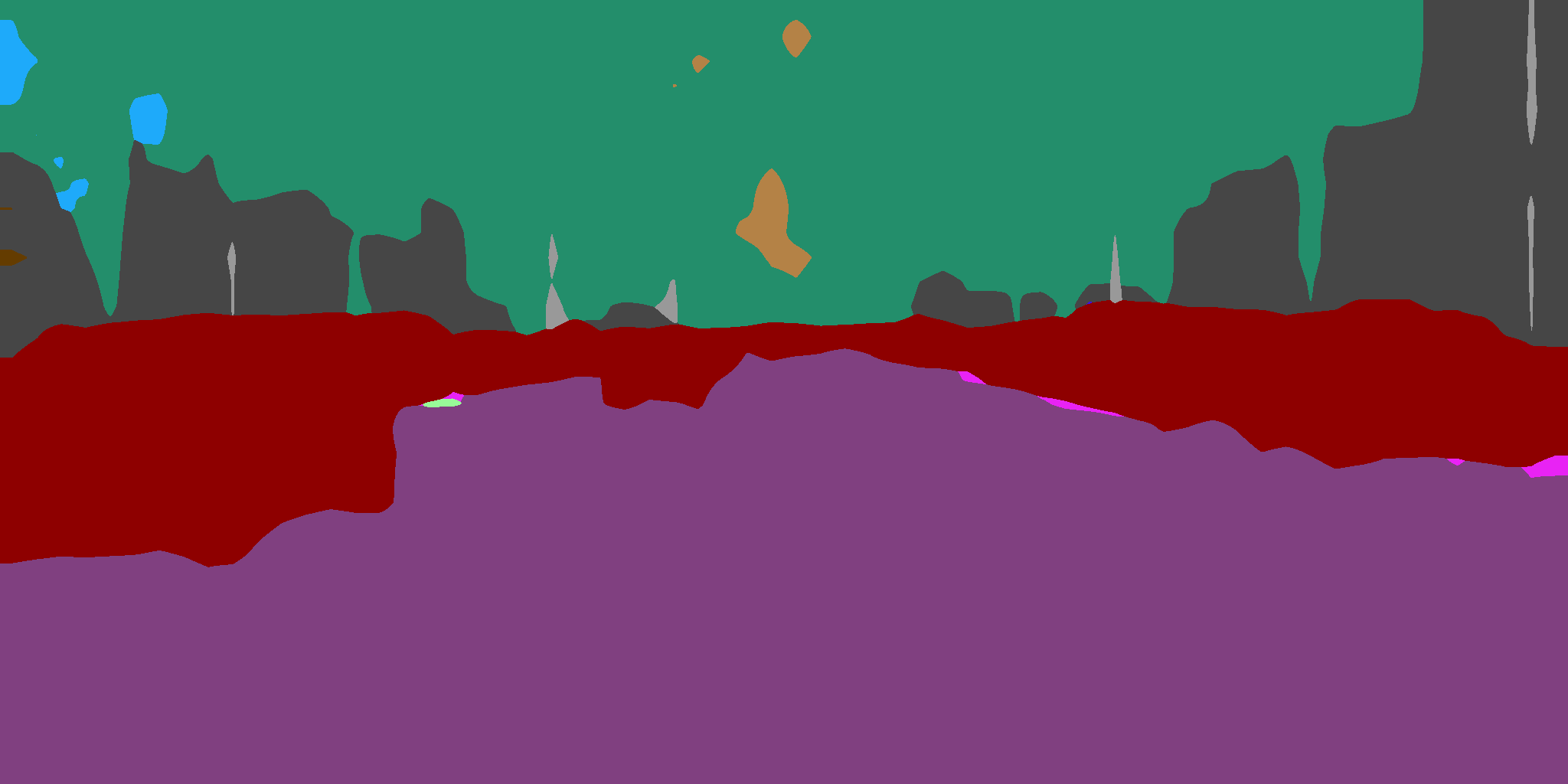

Supplement: S1 Data — (ZIP) [file pone.0295263.s001.zip › ╨┬╜¿╬─╝■╝╨ (2)/groundtruth/berlin_000007_000019_gtFine_labelTrainIds.png]

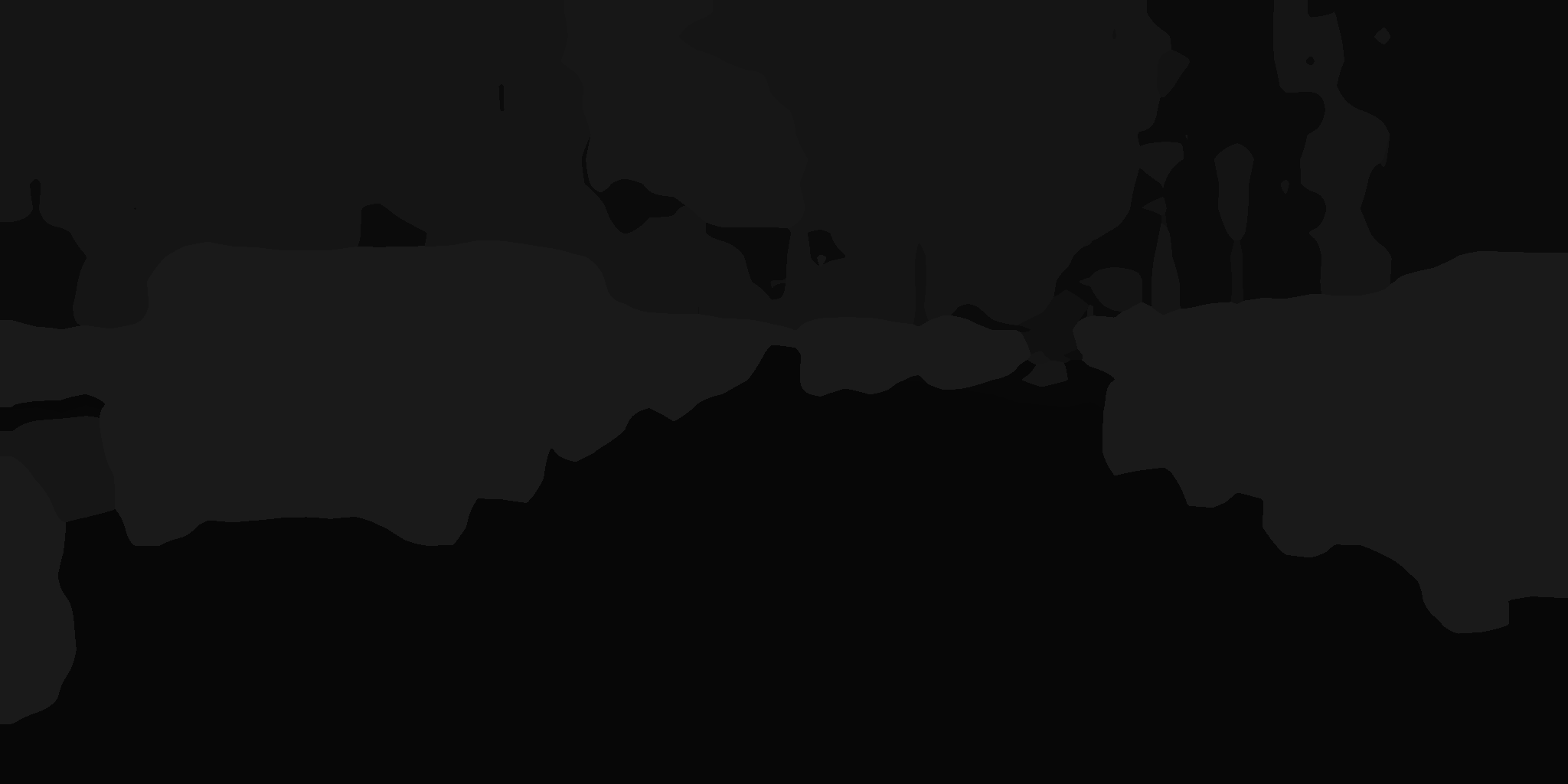

Supplement: S1 Data — (ZIP) [file pone.0295263.s001.zip › ╨┬╜¿╬─╝■╝╨ (2)/groundtruth/berlin_000008_000019_gtFine_labelIds.png]

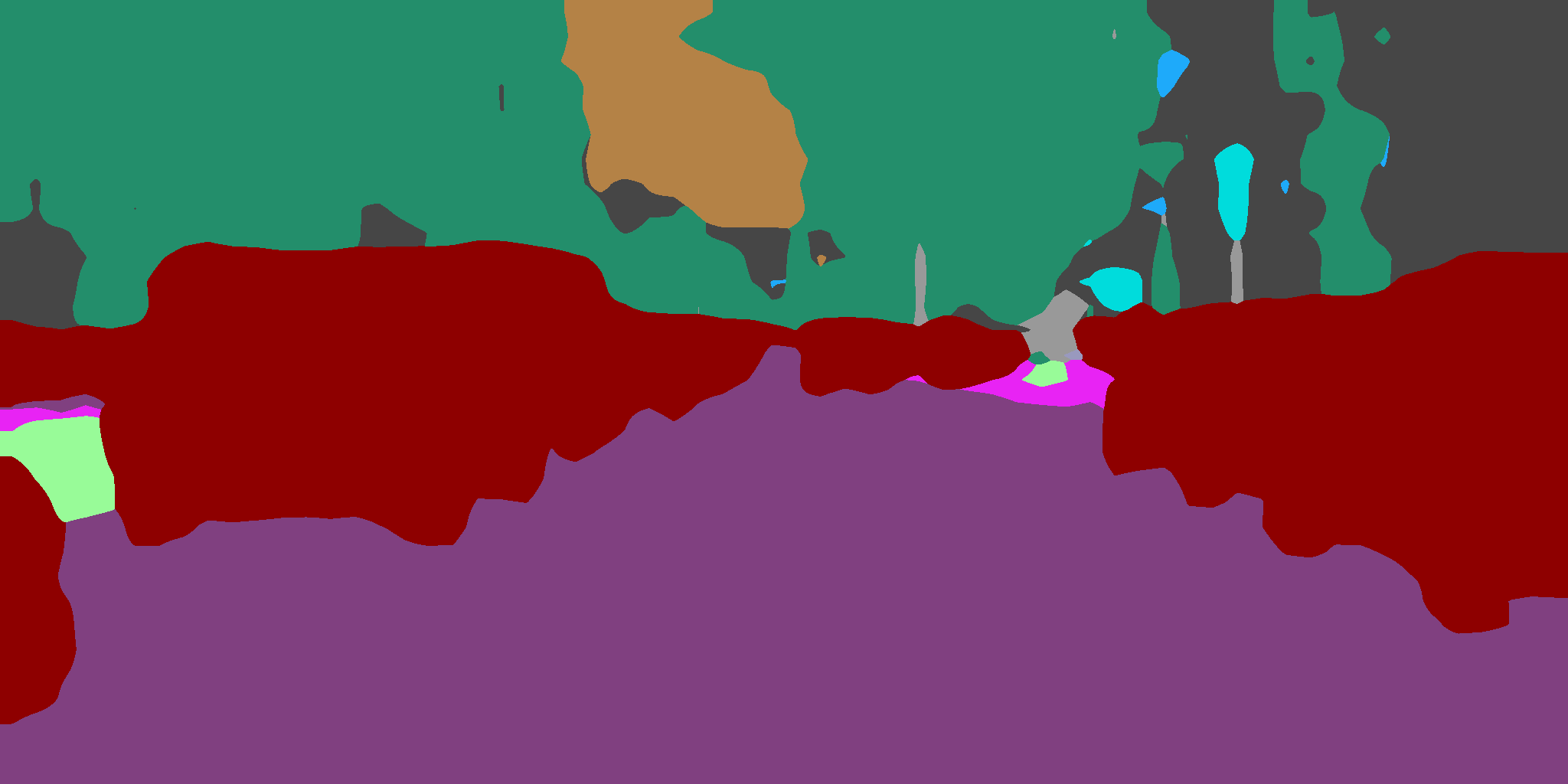

Supplement: S1 Data — (ZIP) [file pone.0295263.s001.zip › ╨┬╜¿╬─╝■╝╨ (2)/groundtruth/berlin_000008_000019_gtFine_labelTrainIds.png]

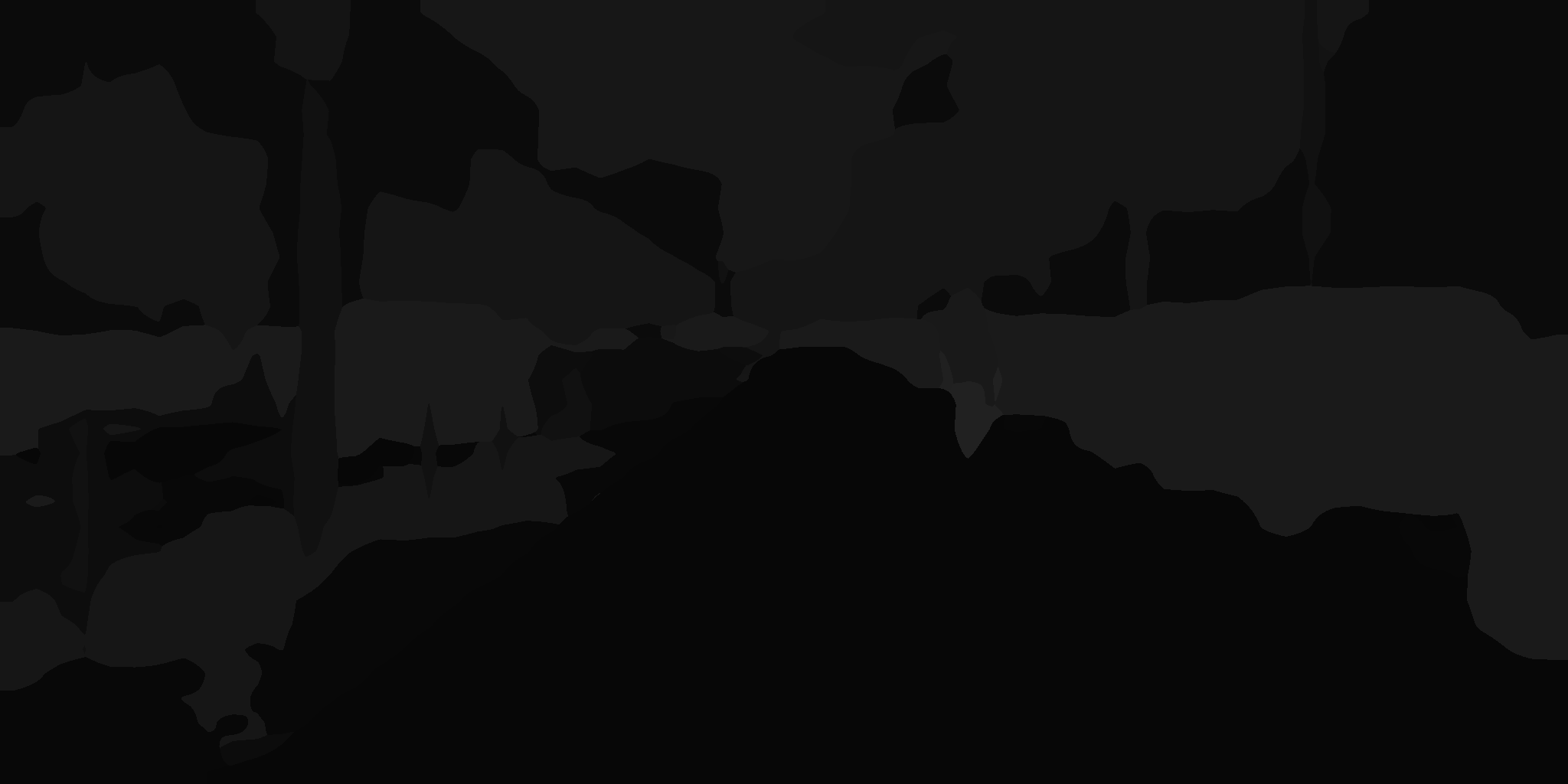

Supplement: S1 Data — (ZIP) [file pone.0295263.s001.zip › ╨┬╜¿╬─╝■╝╨ (2)/groundtruth/berlin_000009_000019_gtFine_labelIds.png]

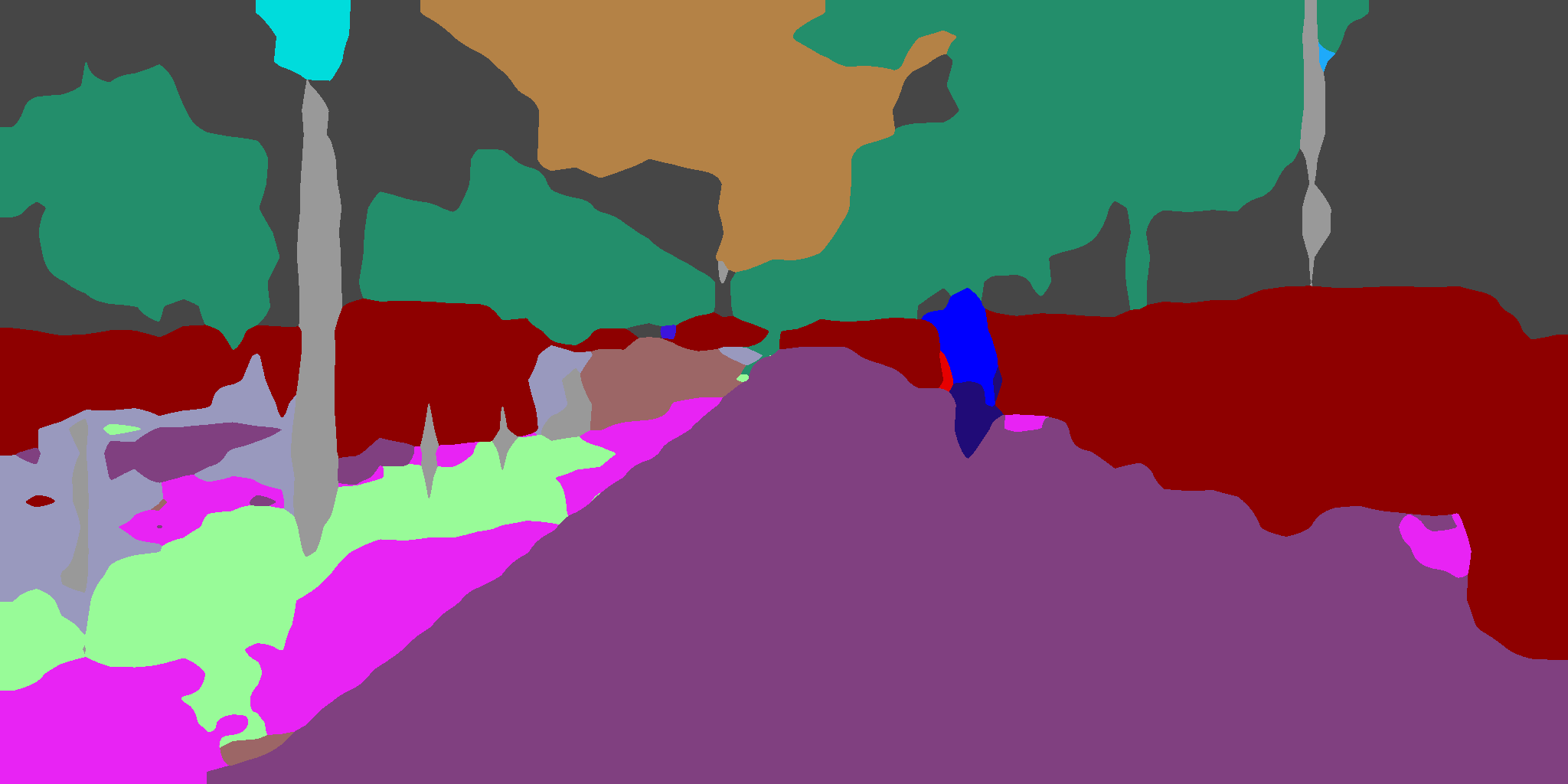

Supplement: S1 Data — (ZIP) [file pone.0295263.s001.zip › ╨┬╜¿╬─╝■╝╨ (2)/groundtruth/berlin_000009_000019_gtFine_labelTrainIds.png]

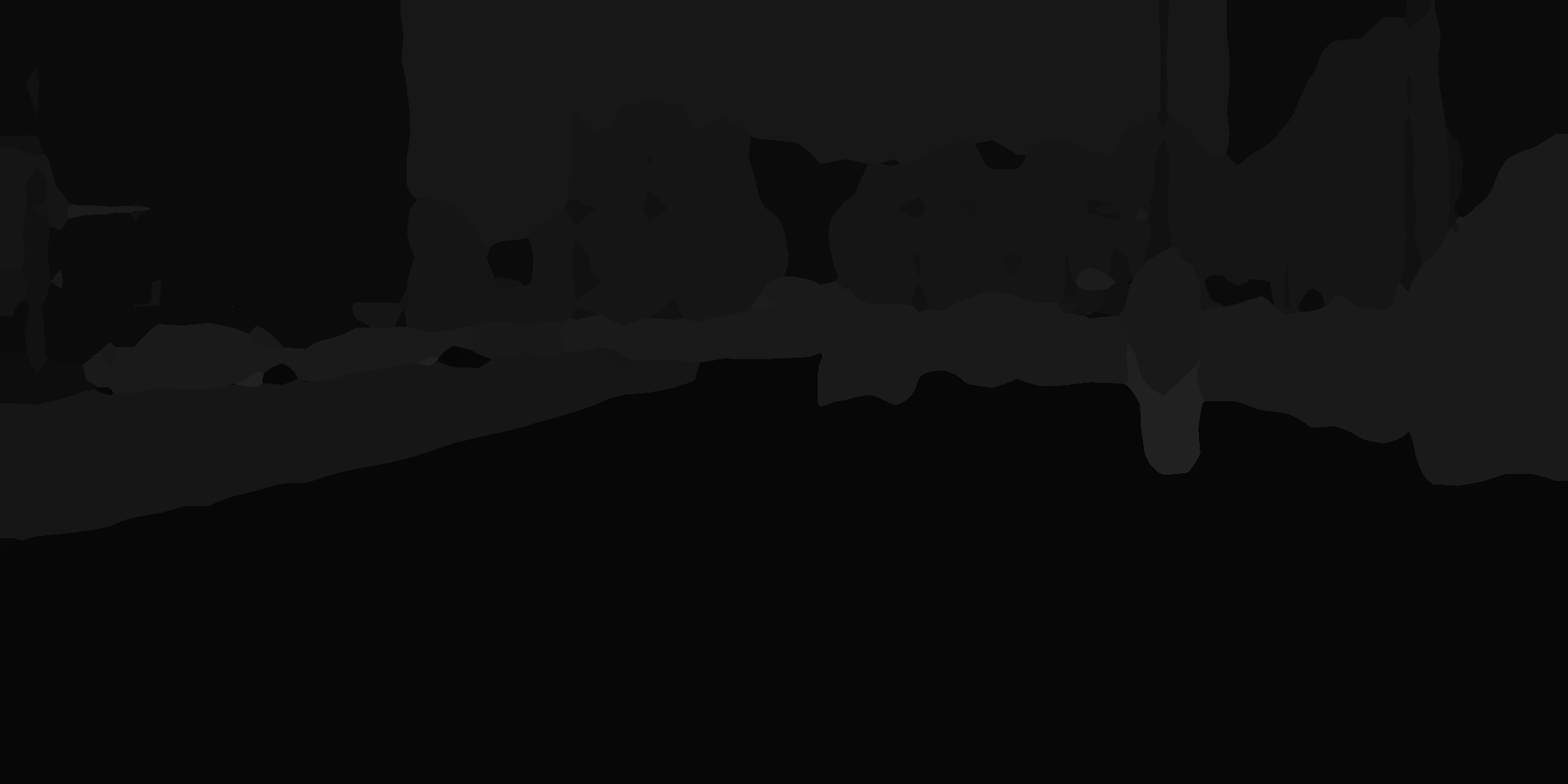

Supplement: S1 Data — (ZIP) [file pone.0295263.s001.zip › ╨┬╜¿╬─╝■╝╨ (2)/groundtruth/berlin_000010_000019_gtFine_labelIds.png]

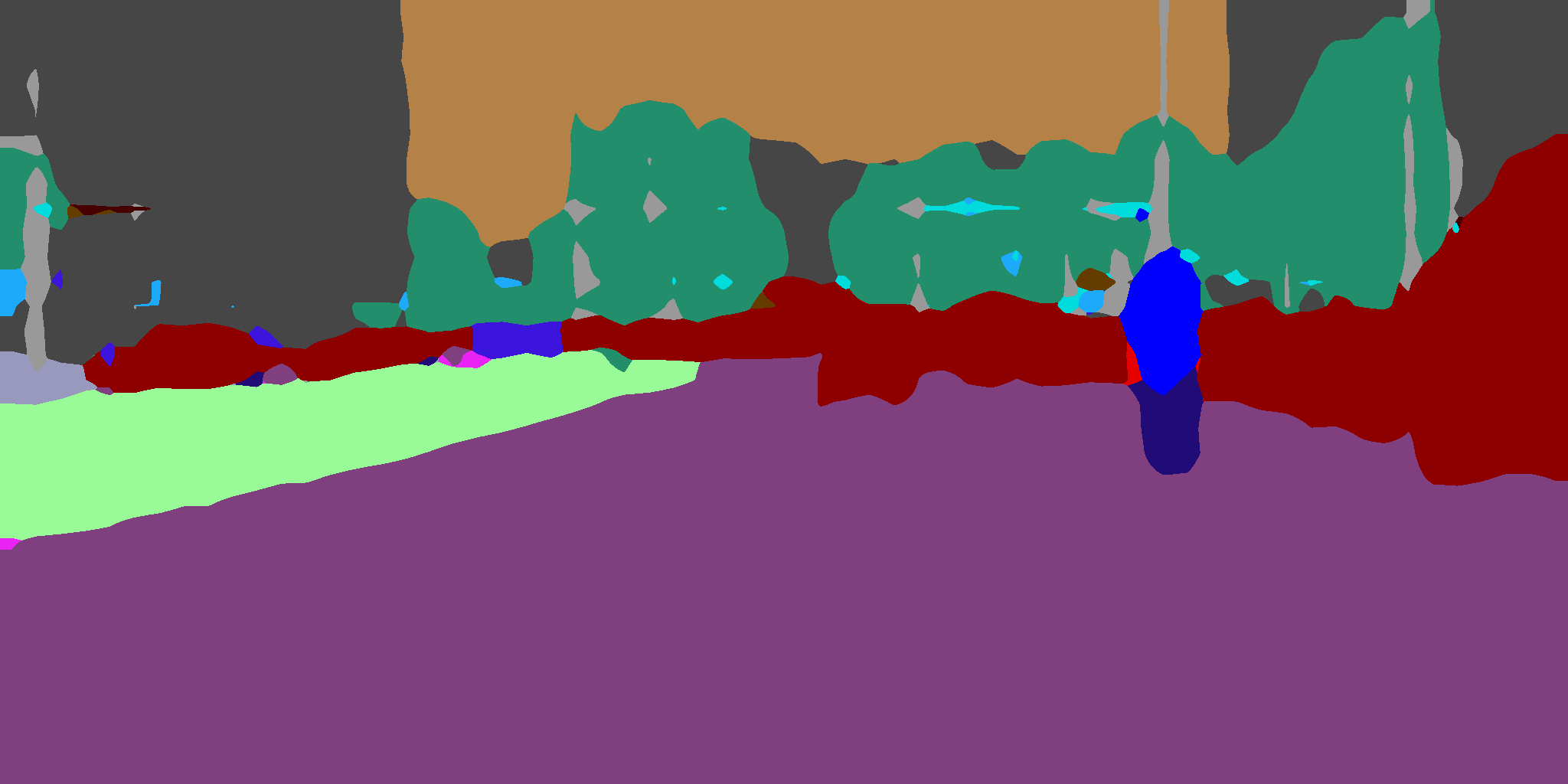

Supplement: S1 Data — (ZIP) [file pone.0295263.s001.zip › ╨┬╜¿╬─╝■╝╨ (2)/groundtruth/berlin_000010_000019_gtFine_labelTrainIds.png]

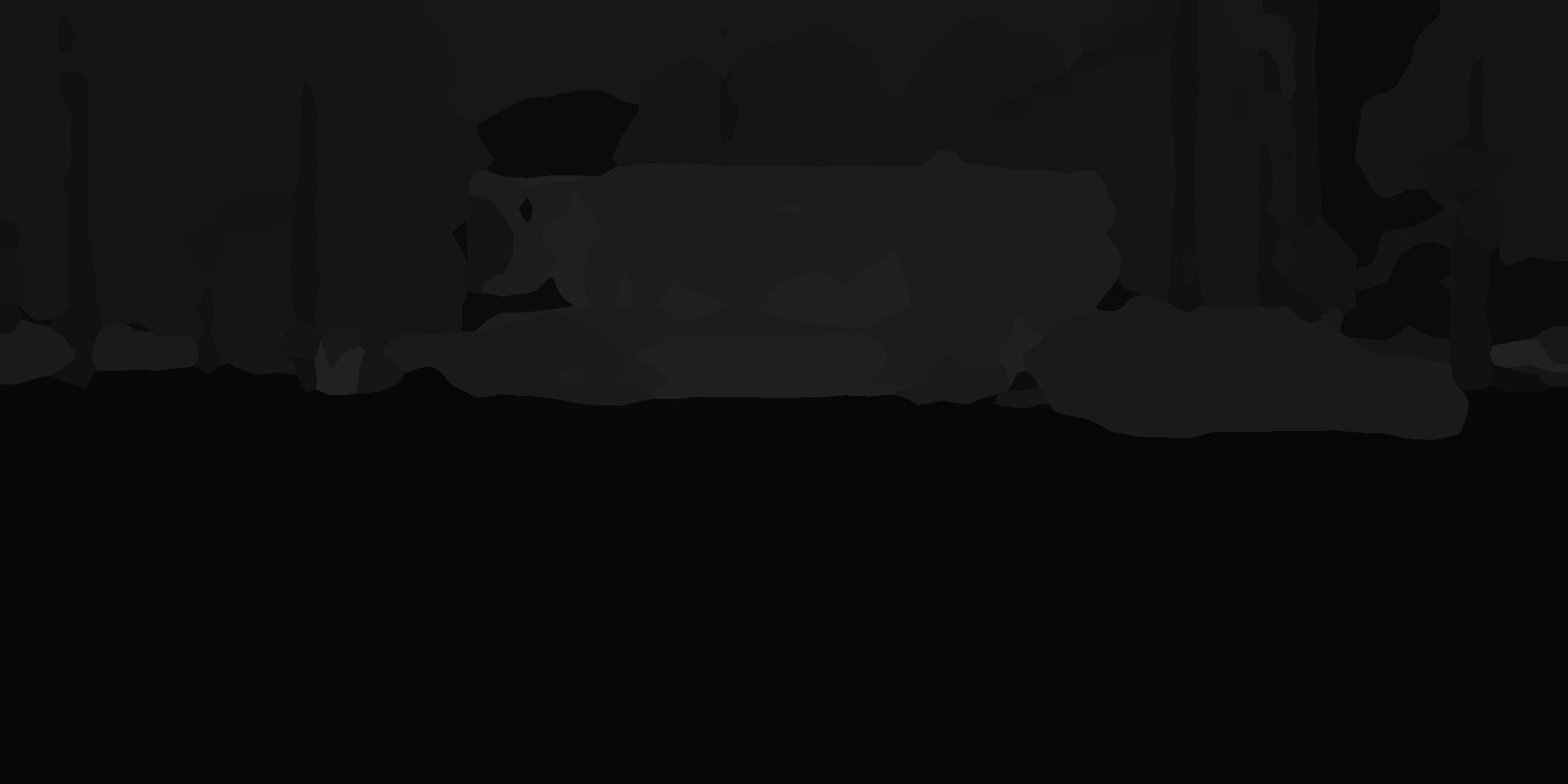

Supplement: S1 Data — (ZIP) [file pone.0295263.s001.zip › ╨┬╜¿╬─╝■╝╨ (2)/groundtruth/berlin_000011_000019_gtFine_labelIds.png]

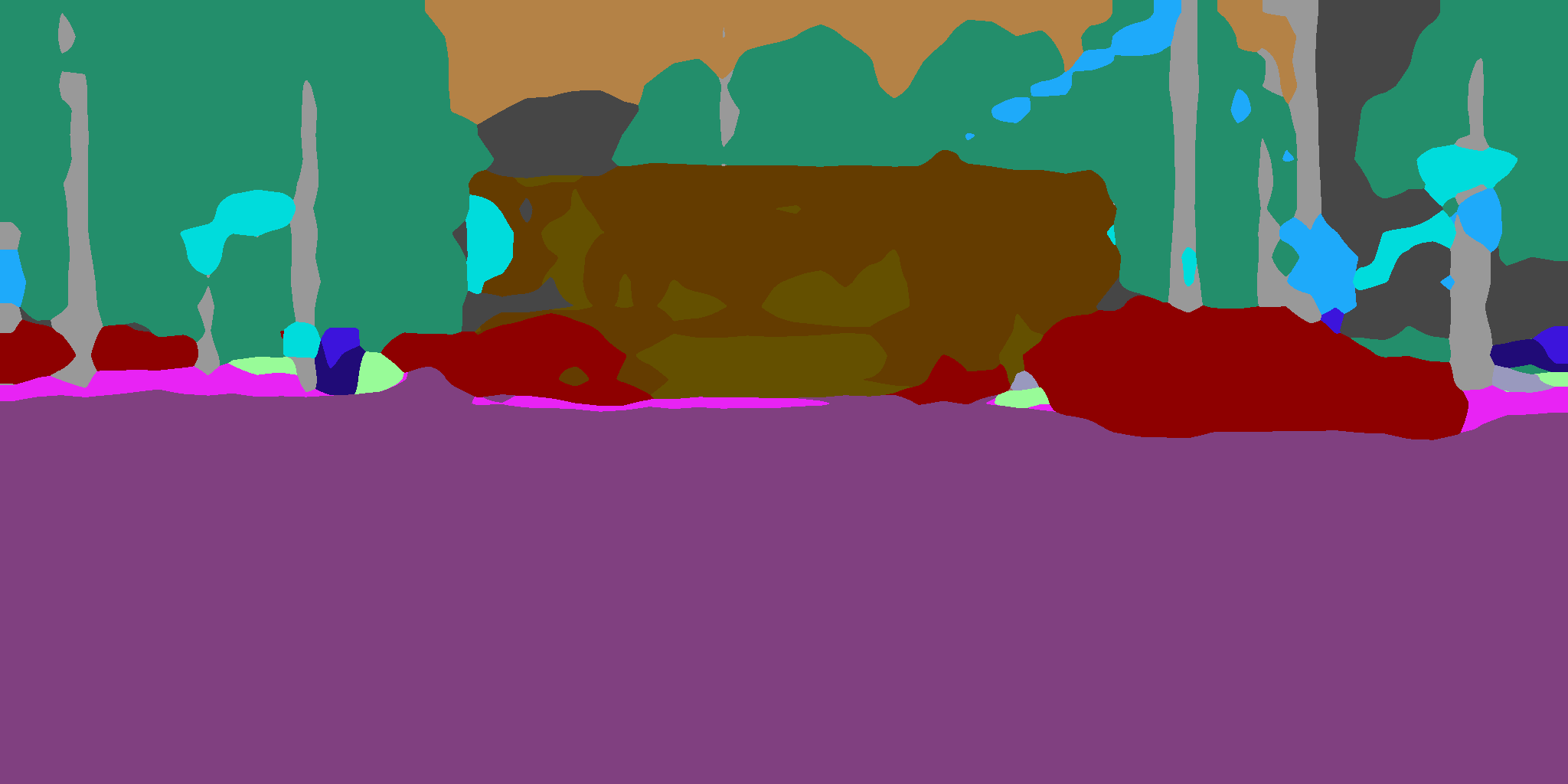

Supplement: S1 Data — (ZIP) [file pone.0295263.s001.zip › ╨┬╜¿╬─╝■╝╨ (2)/groundtruth/berlin_000011_000019_gtFine_labelTrainIds.png]

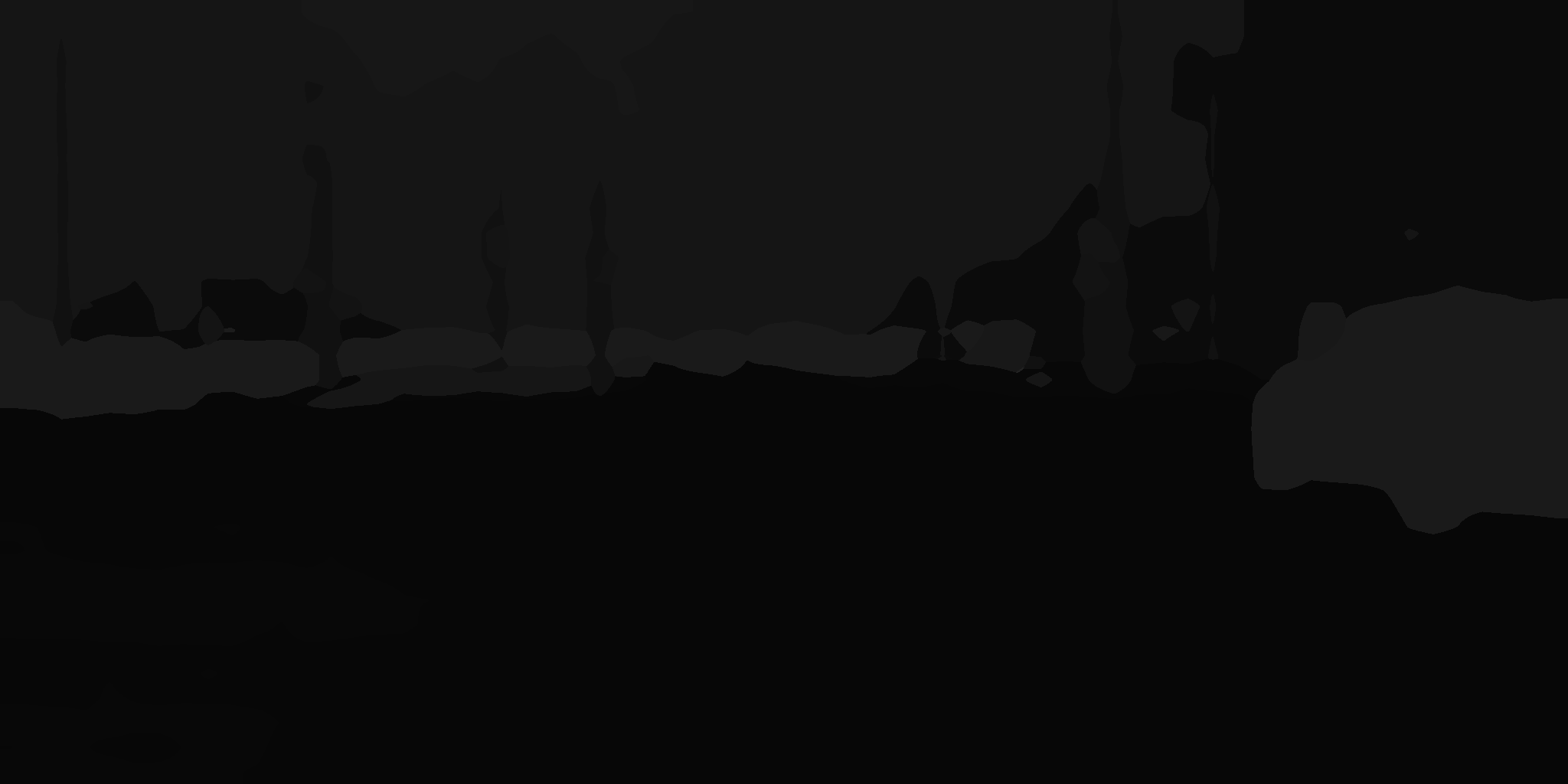

Supplement: S1 Data — (ZIP) [file pone.0295263.s001.zip › ╨┬╜¿╬─╝■╝╨ (2)/groundtruth/berlin_000012_000019_gtFine_labelIds.png]

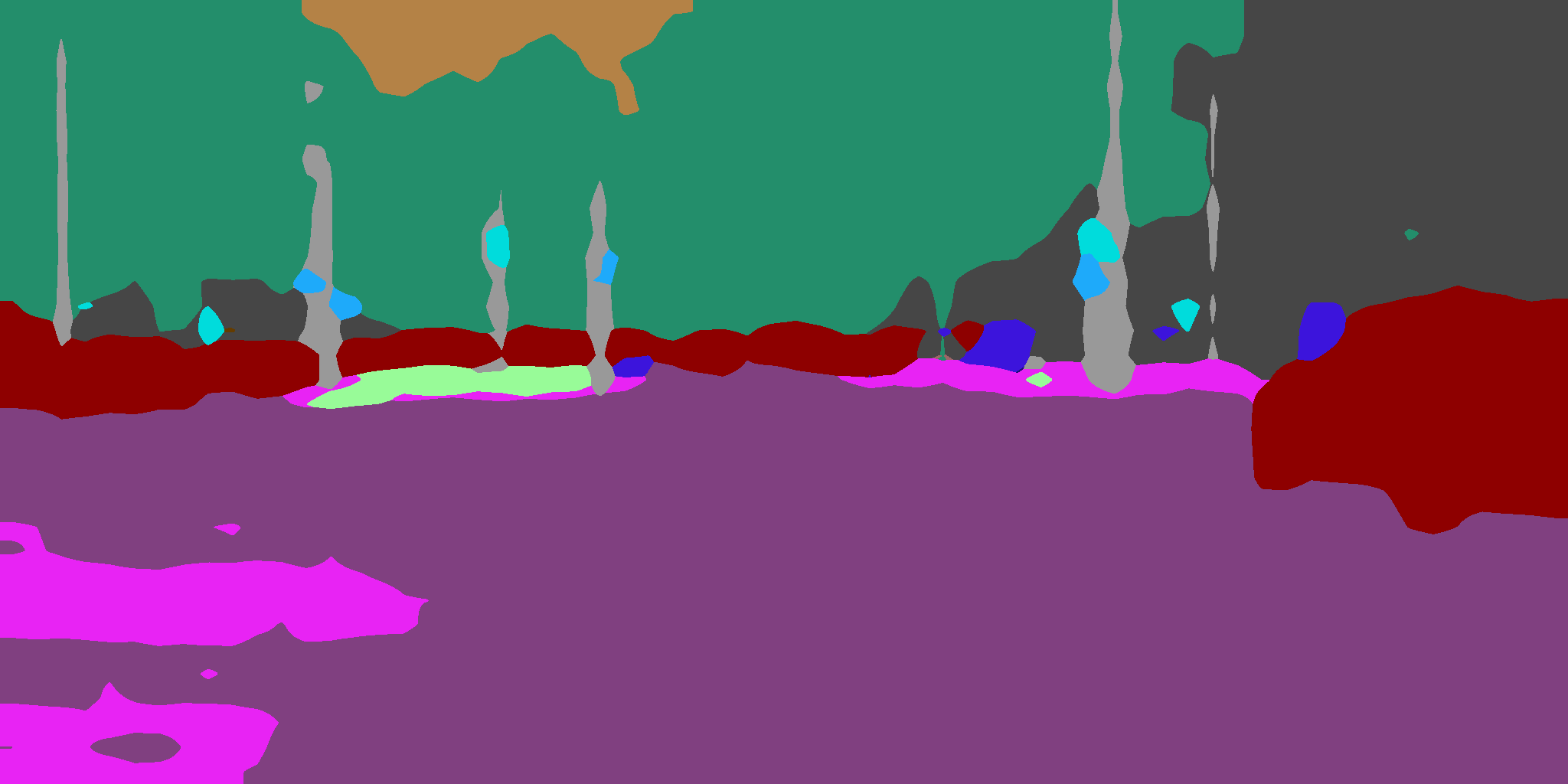

Supplement: S1 Data — (ZIP) [file pone.0295263.s001.zip › ╨┬╜¿╬─╝■╝╨ (2)/groundtruth/berlin_000012_000019_gtFine_labelTrainIds.png]

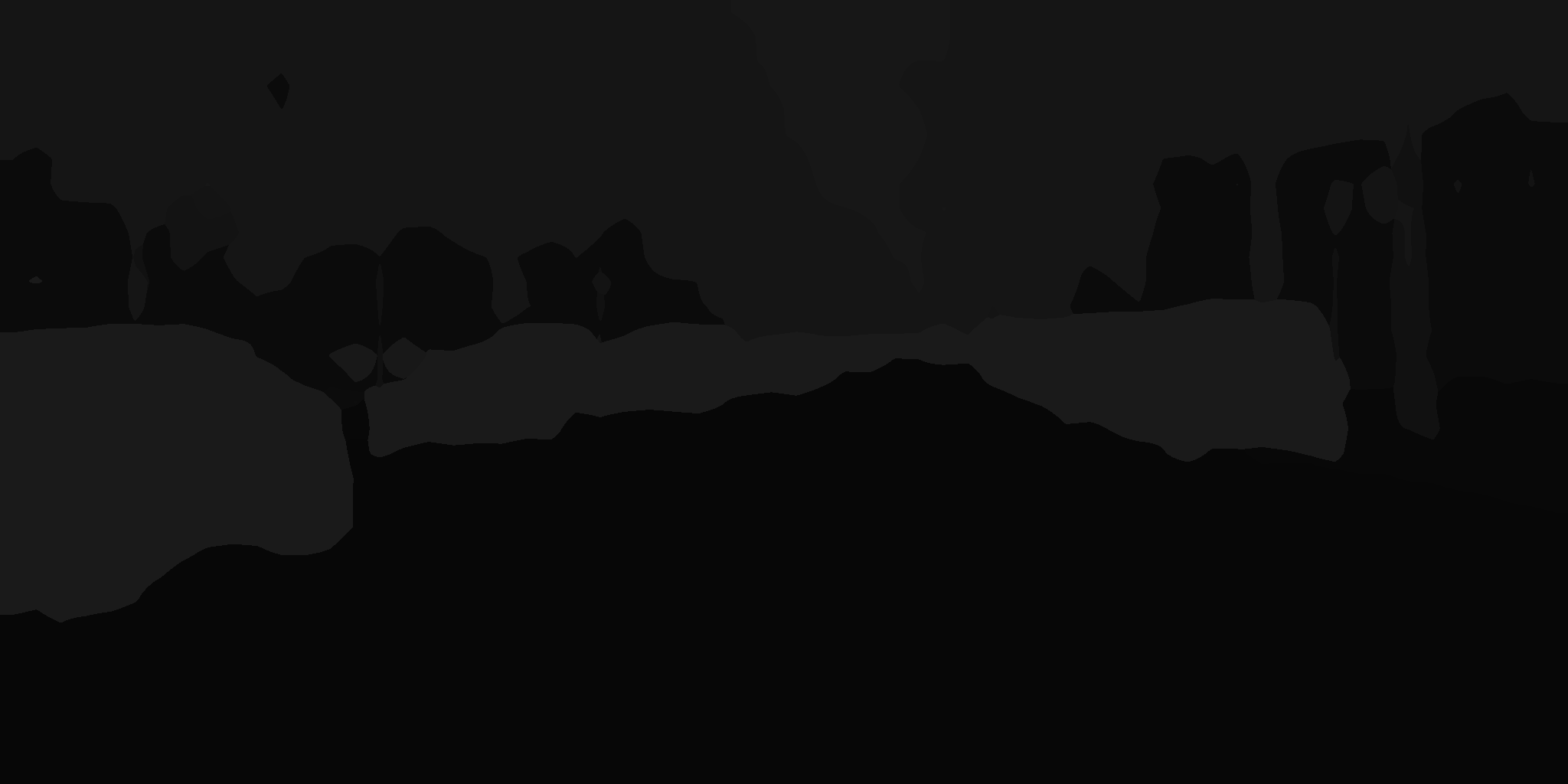

Supplement: S1 Data — (ZIP) [file pone.0295263.s001.zip › ╨┬╜¿╬─╝■╝╨ (2)/groundtruth/berlin_000013_000019_gtFine_labelIds.png]

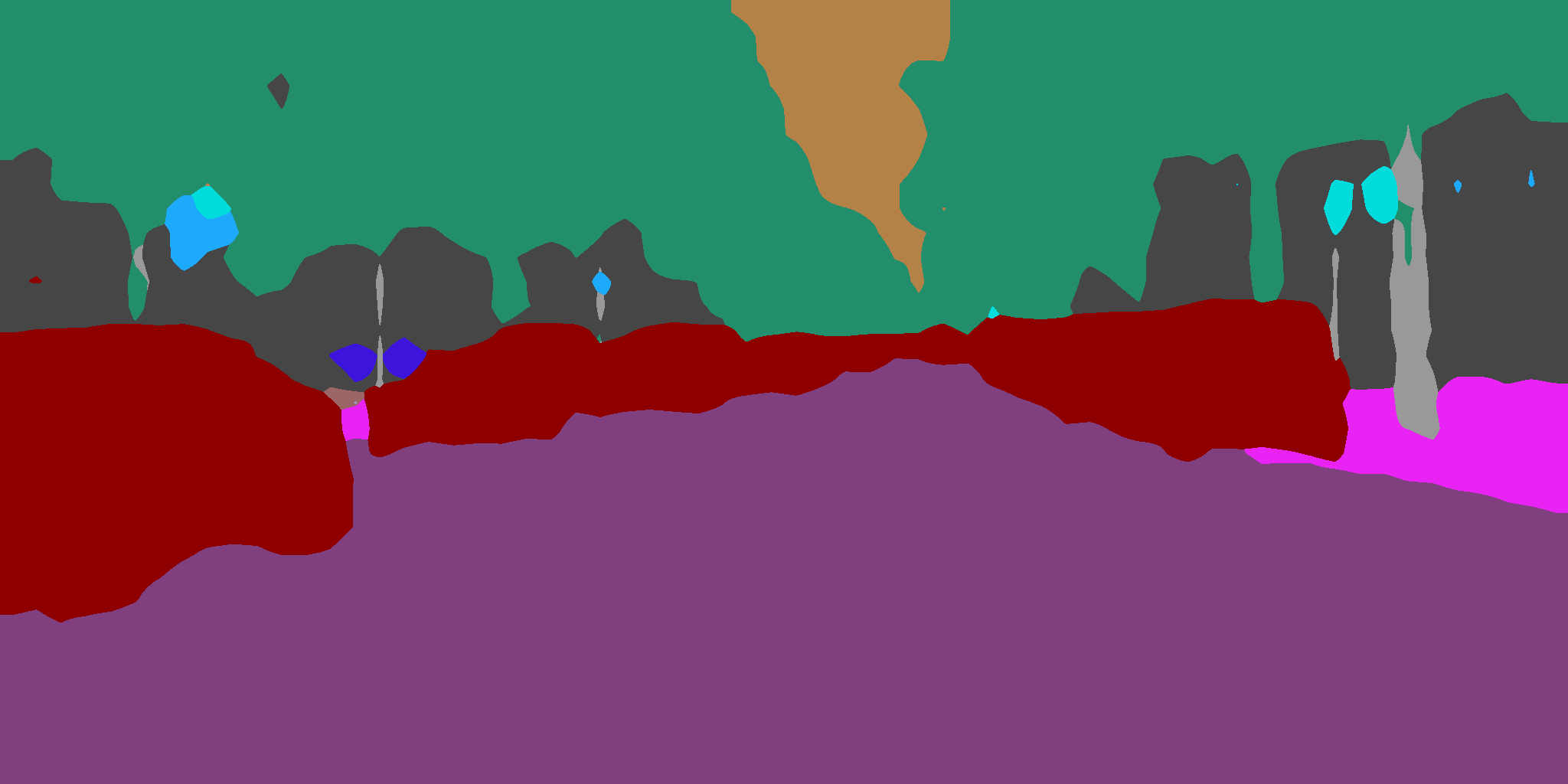

Supplement: S1 Data — (ZIP) [file pone.0295263.s001.zip › ╨┬╜¿╬─╝■╝╨ (2)/groundtruth/berlin_000013_000019_gtFine_labelTrainIds.png]

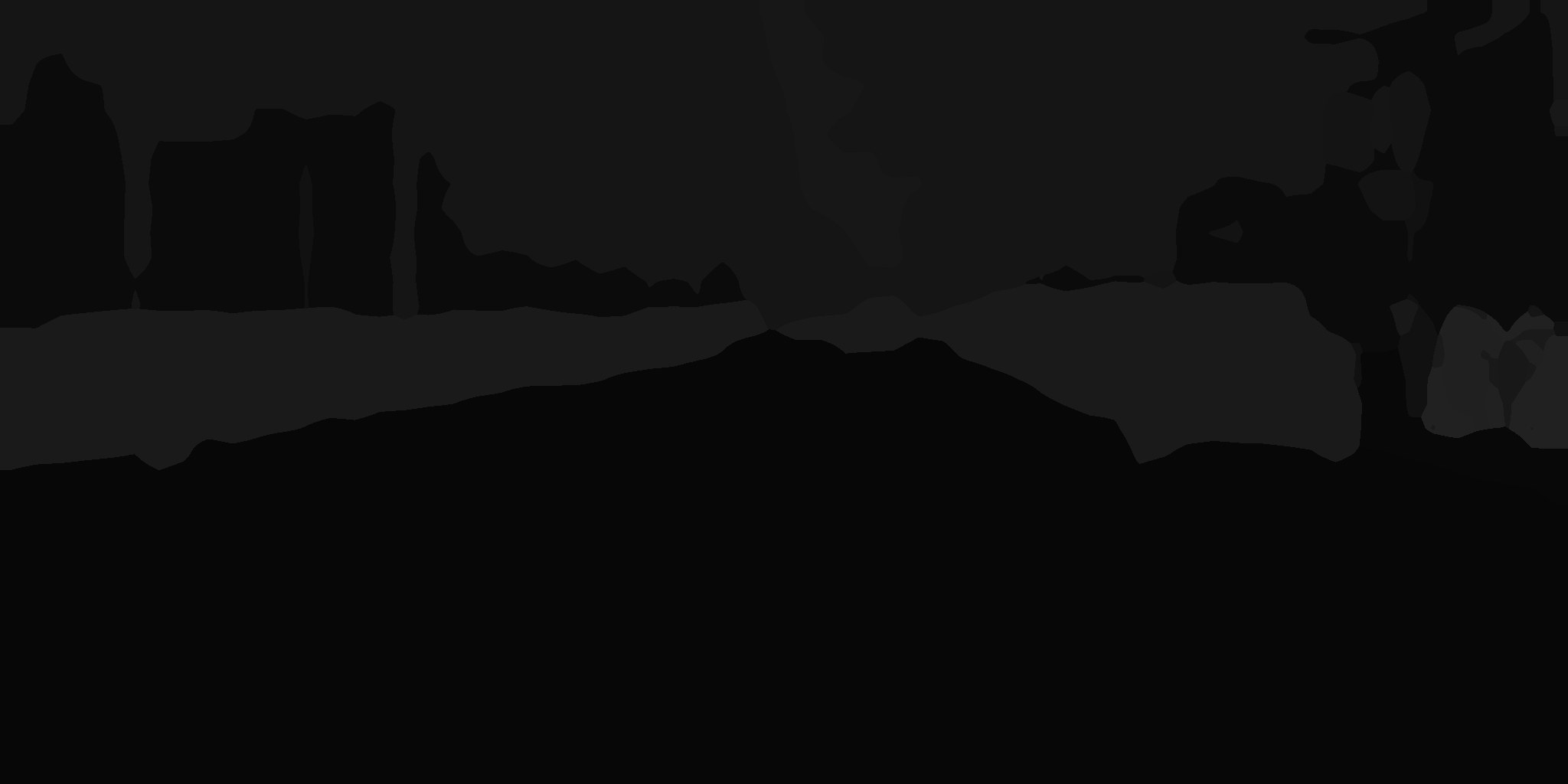

Supplement: S1 Data — (ZIP) [file pone.0295263.s001.zip › ╨┬╜¿╬─╝■╝╨ (2)/groundtruth/berlin_000014_000019_gtFine_labelIds.png]

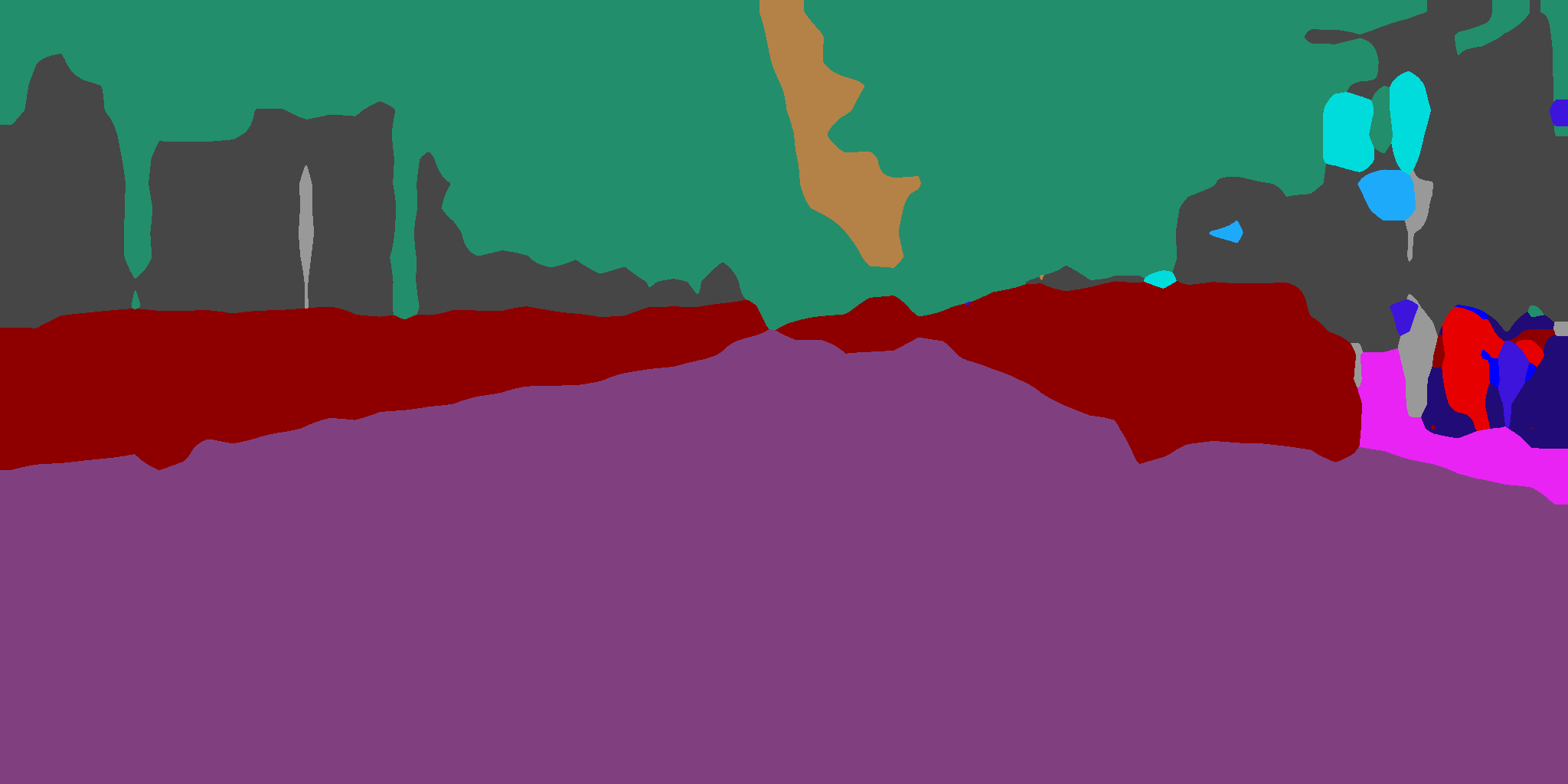

Supplement: S1 Data — (ZIP) [file pone.0295263.s001.zip › ╨┬╜¿╬─╝■╝╨ (2)/groundtruth/berlin_000014_000019_gtFine_labelTrainIds.png]

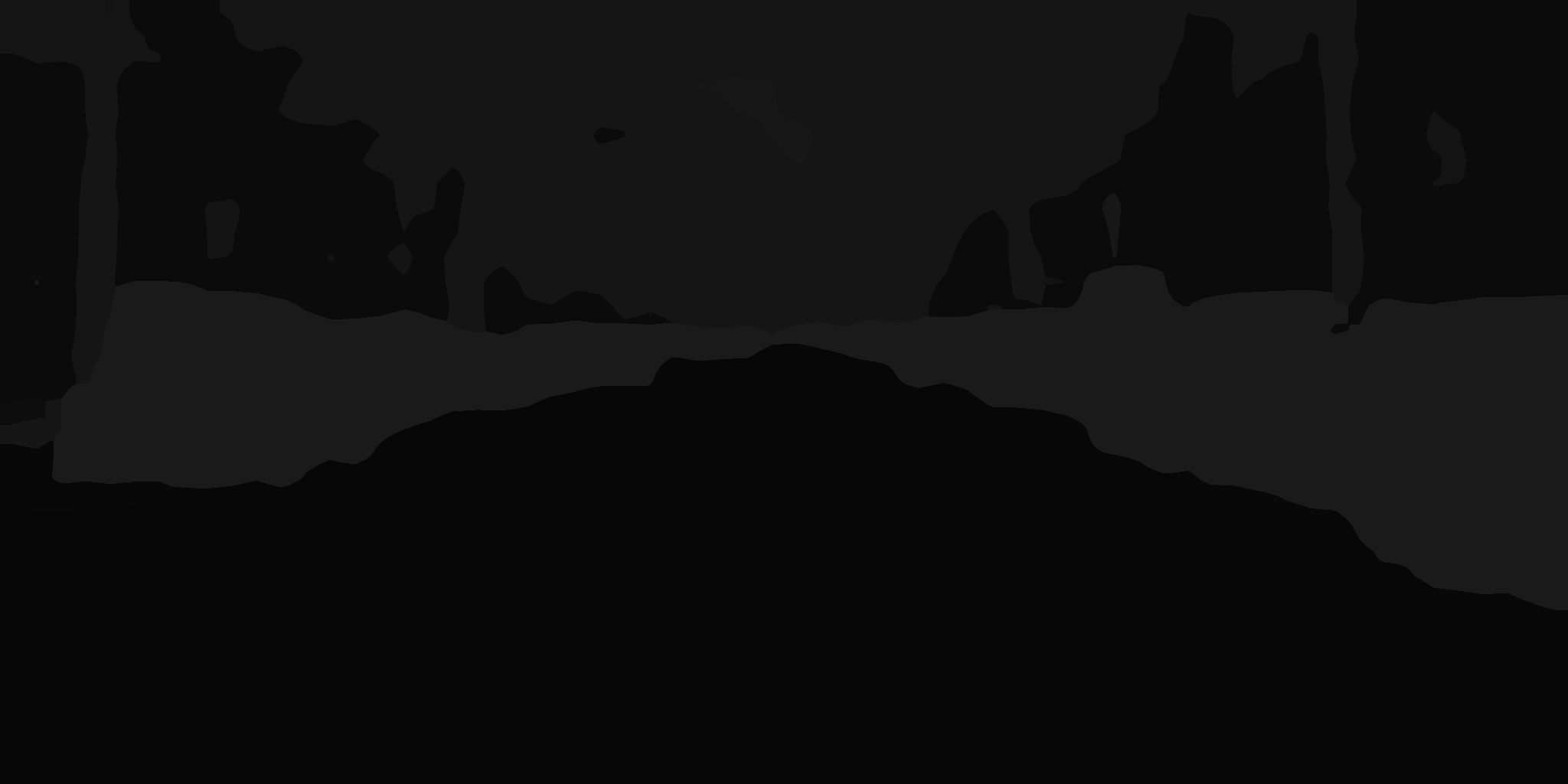

Supplement: S1 Data — (ZIP) [file pone.0295263.s001.zip › ╨┬╜¿╬─╝■╝╨ (2)/groundtruth/berlin_000015_000019_gtFine_labelIds.png]

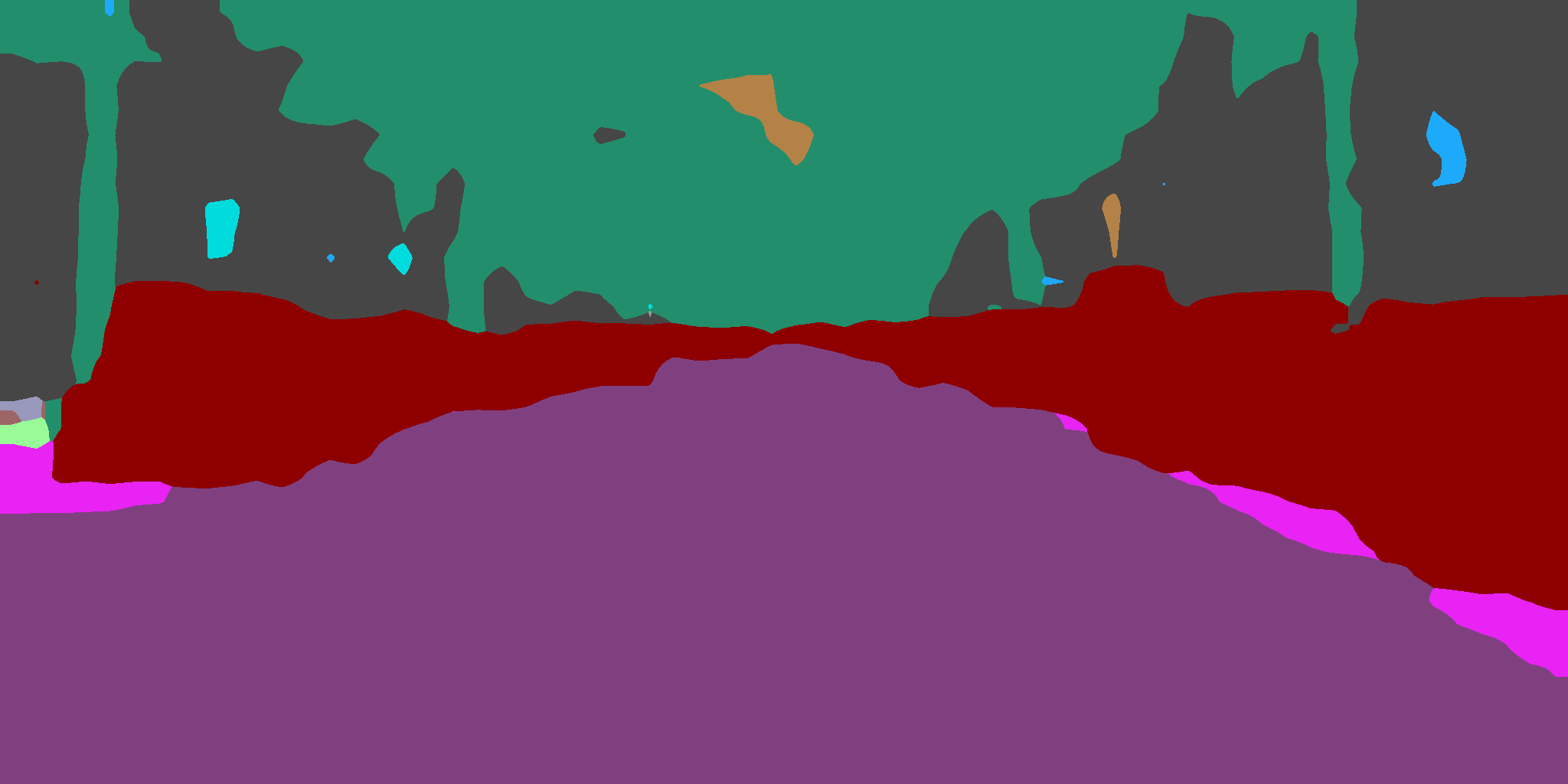

Supplement: S1 Data — (ZIP) [file pone.0295263.s001.zip › ╨┬╜¿╬─╝■╝╨ (2)/groundtruth/berlin_000015_000019_gtFine_labelTrainIds.png]

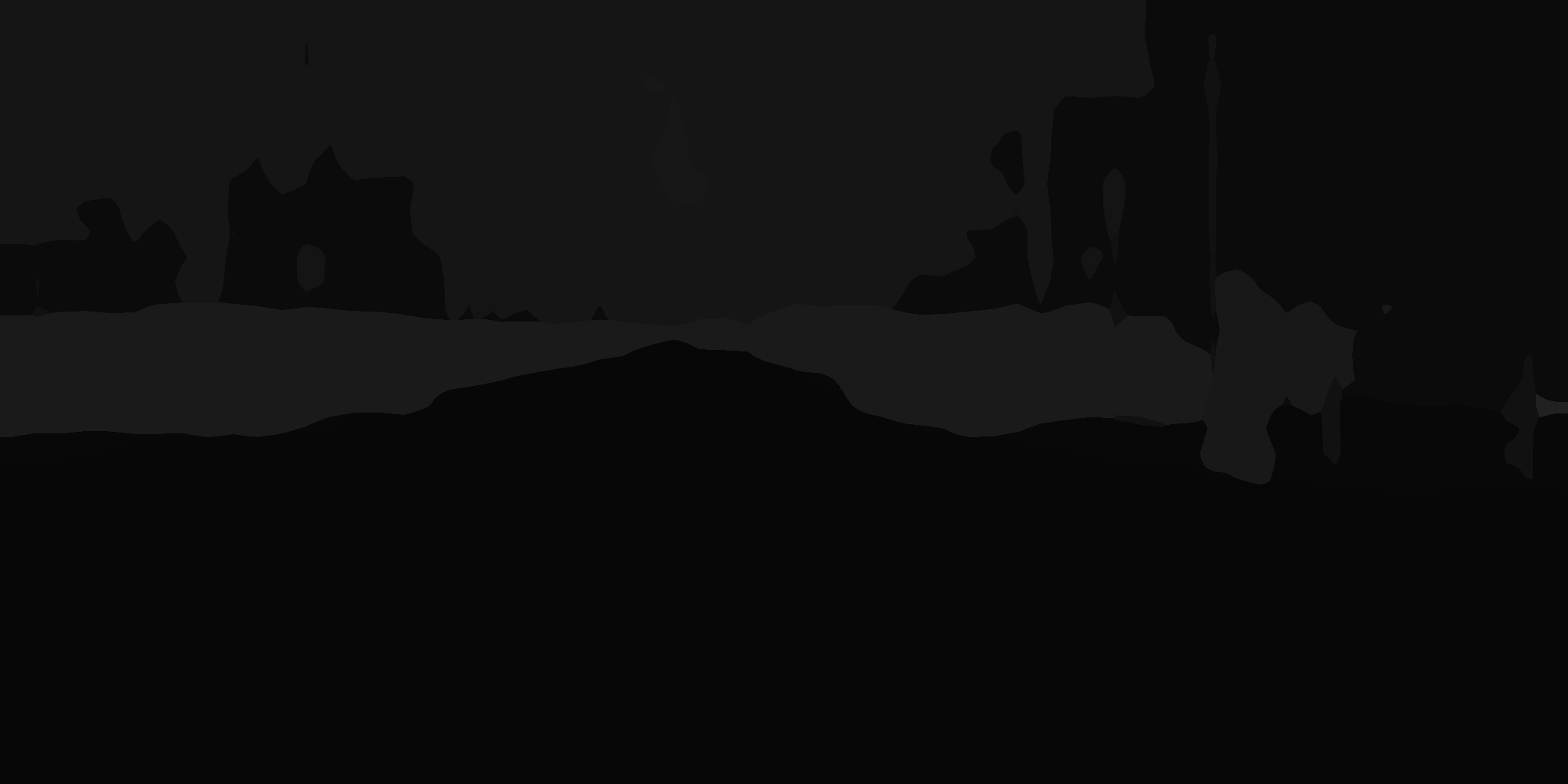

Supplement: S1 Data — (ZIP) [file pone.0295263.s001.zip › ╨┬╜¿╬─╝■╝╨ (2)/groundtruth/berlin_000016_000019_gtFine_labelIds.png]

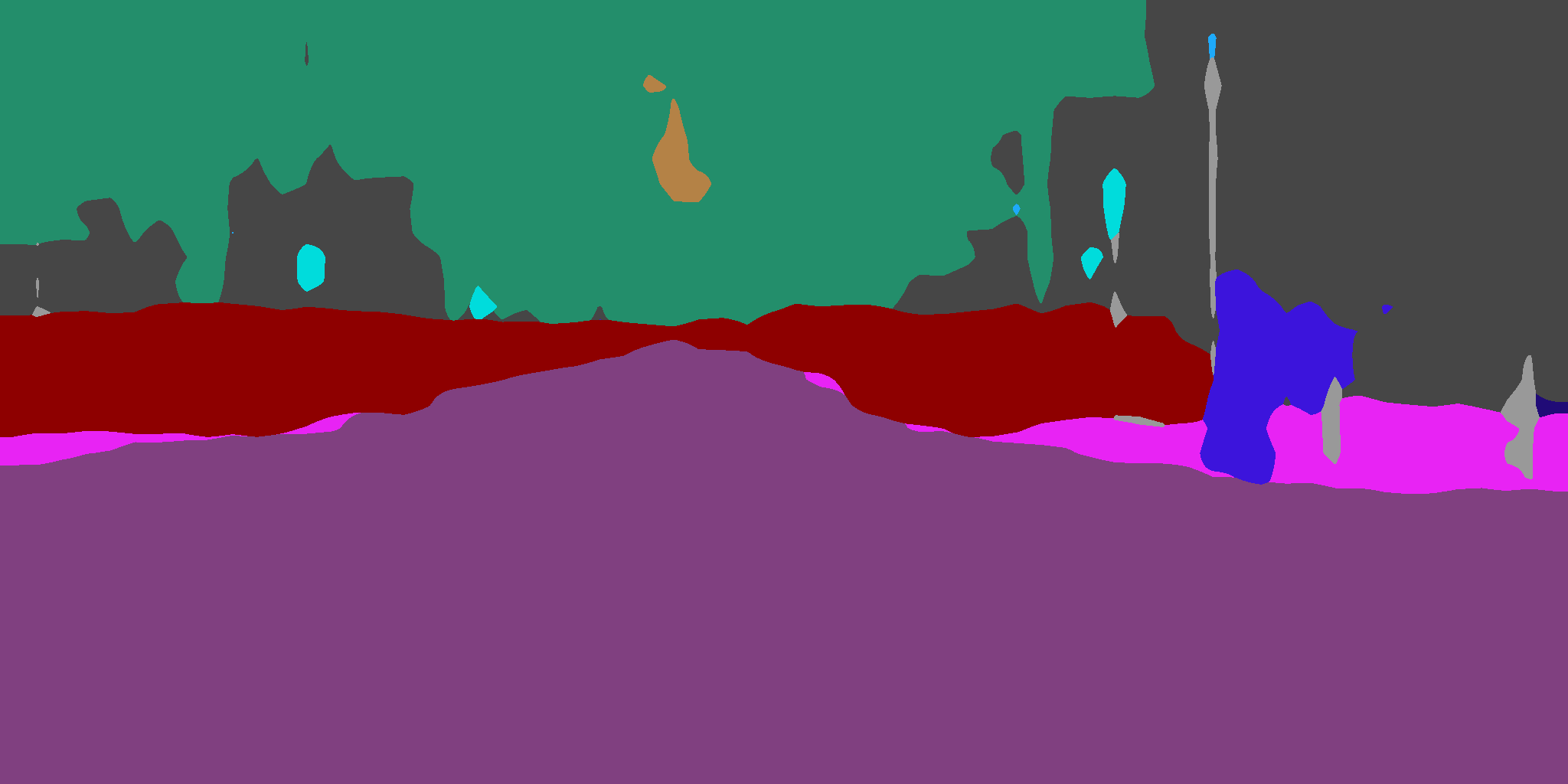

Supplement: S1 Data — (ZIP) [file pone.0295263.s001.zip › ╨┬╜¿╬─╝■╝╨ (2)/groundtruth/berlin_000016_000019_gtFine_labelTrainIds.png]

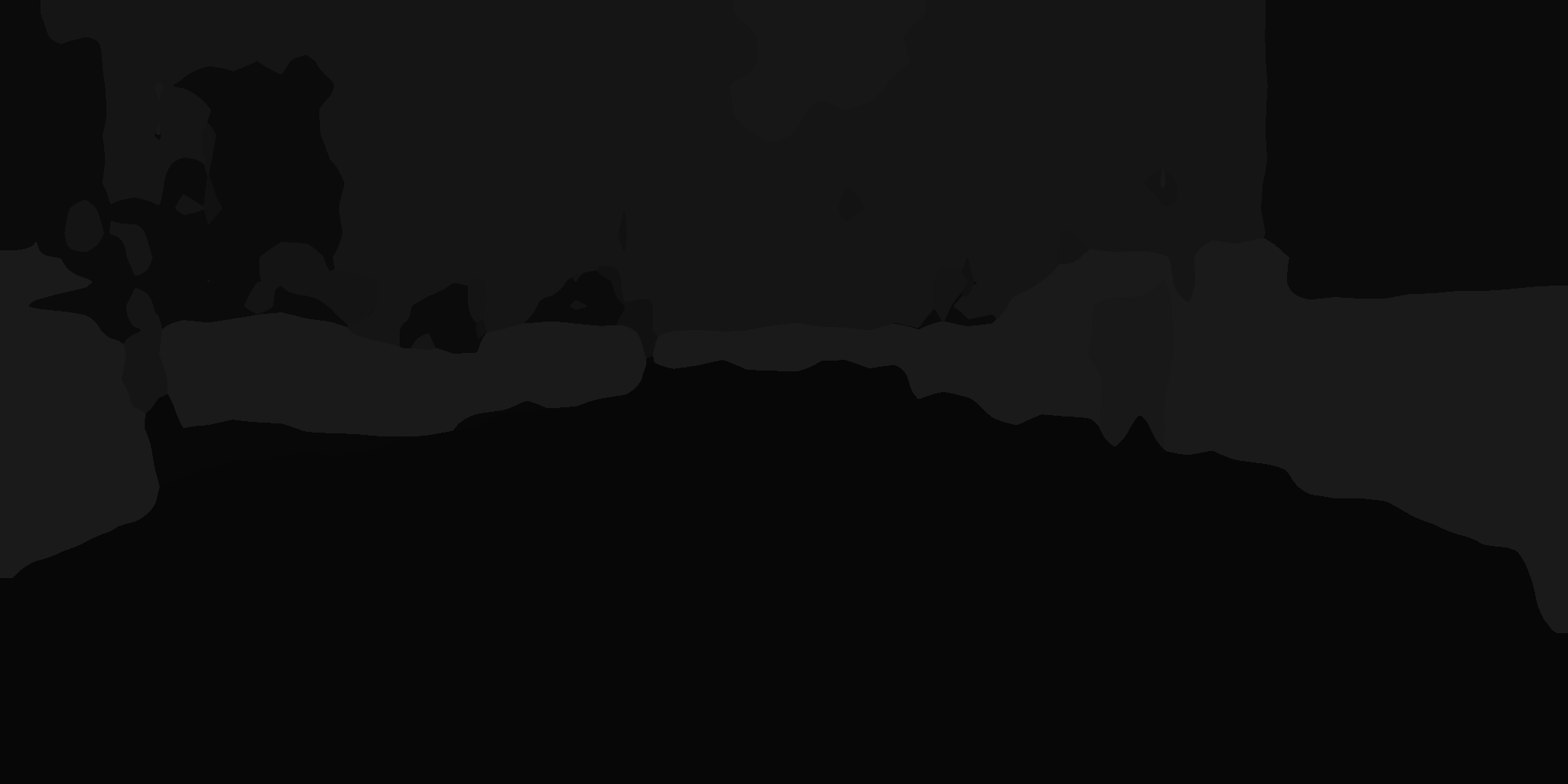

Supplement: S1 Data — (ZIP) [file pone.0295263.s001.zip › ╨┬╜¿╬─╝■╝╨ (2)/groundtruth/berlin_000017_000019_gtFine_labelIds.png]

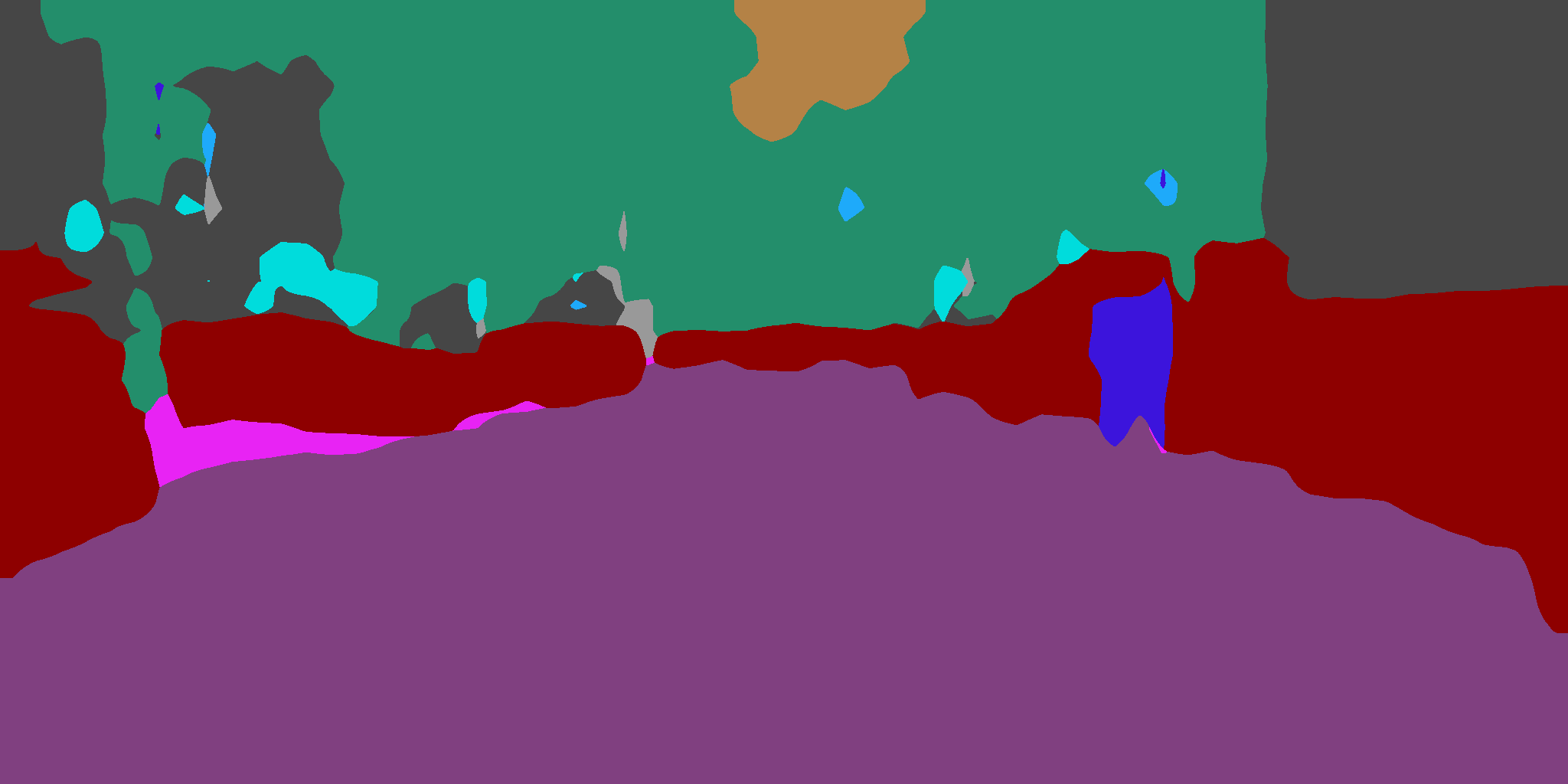

Supplement: S1 Data — (ZIP) [file pone.0295263.s001.zip › ╨┬╜¿╬─╝■╝╨ (2)/groundtruth/berlin_000017_000019_gtFine_labelTrainIds.png]

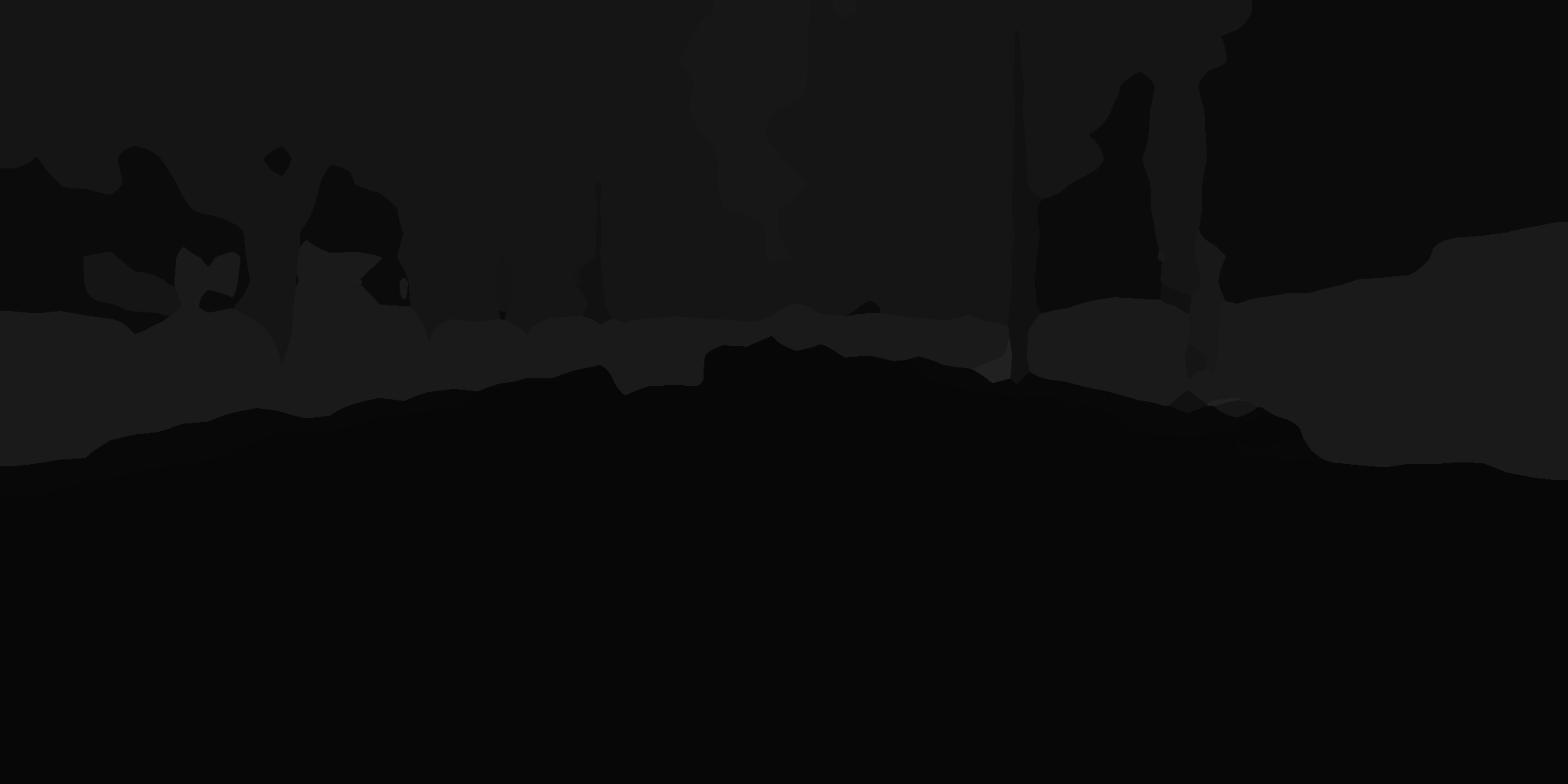

Supplement: S1 Data — (ZIP) [file pone.0295263.s001.zip › ╨┬╜¿╬─╝■╝╨ (2)/groundtruth/berlin_000018_000019_gtFine_labelIds.png]

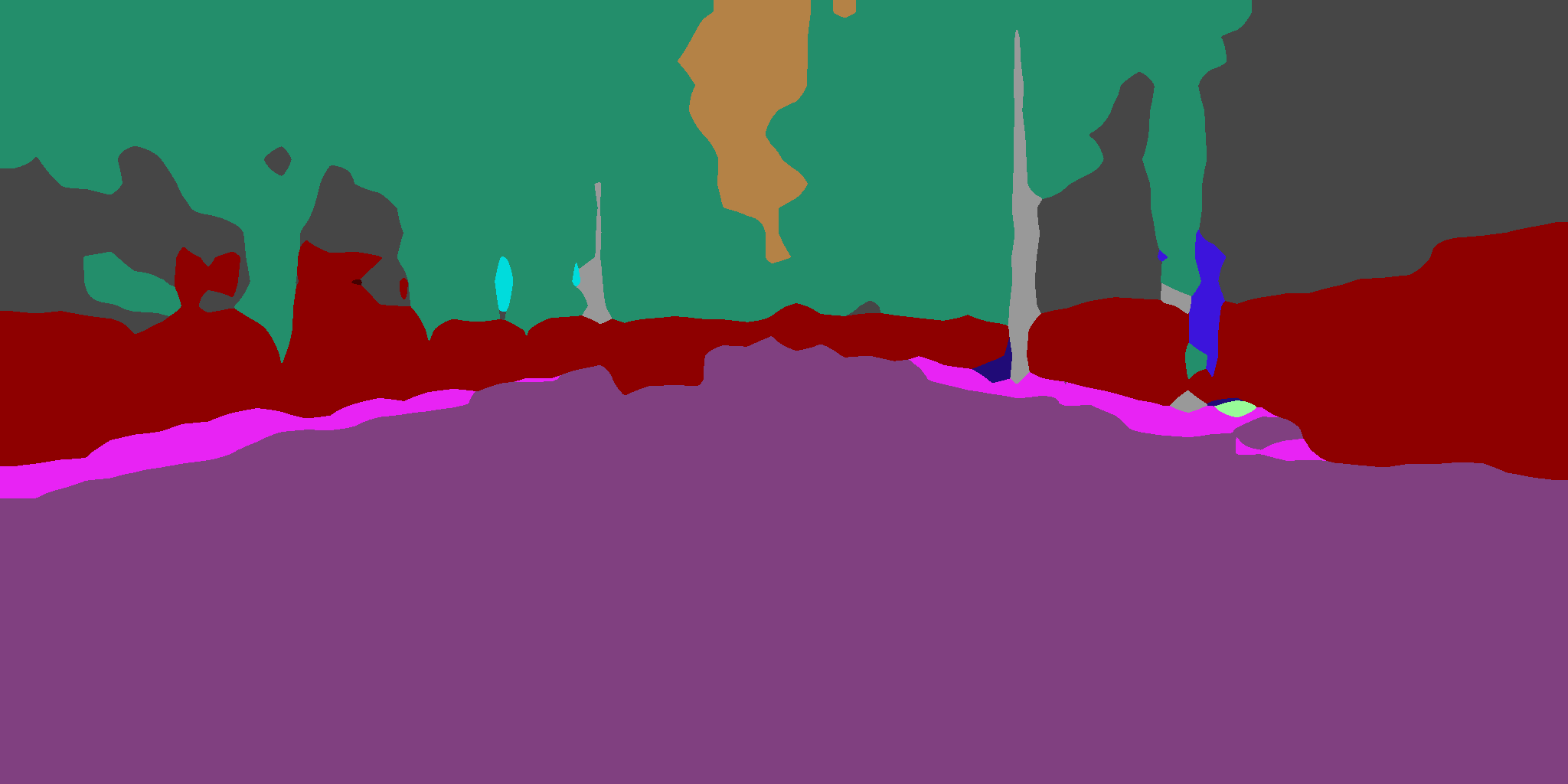

Supplement: S1 Data — (ZIP) [file pone.0295263.s001.zip › ╨┬╜¿╬─╝■╝╨ (2)/groundtruth/berlin_000018_000019_gtFine_labelTrainIds.png]

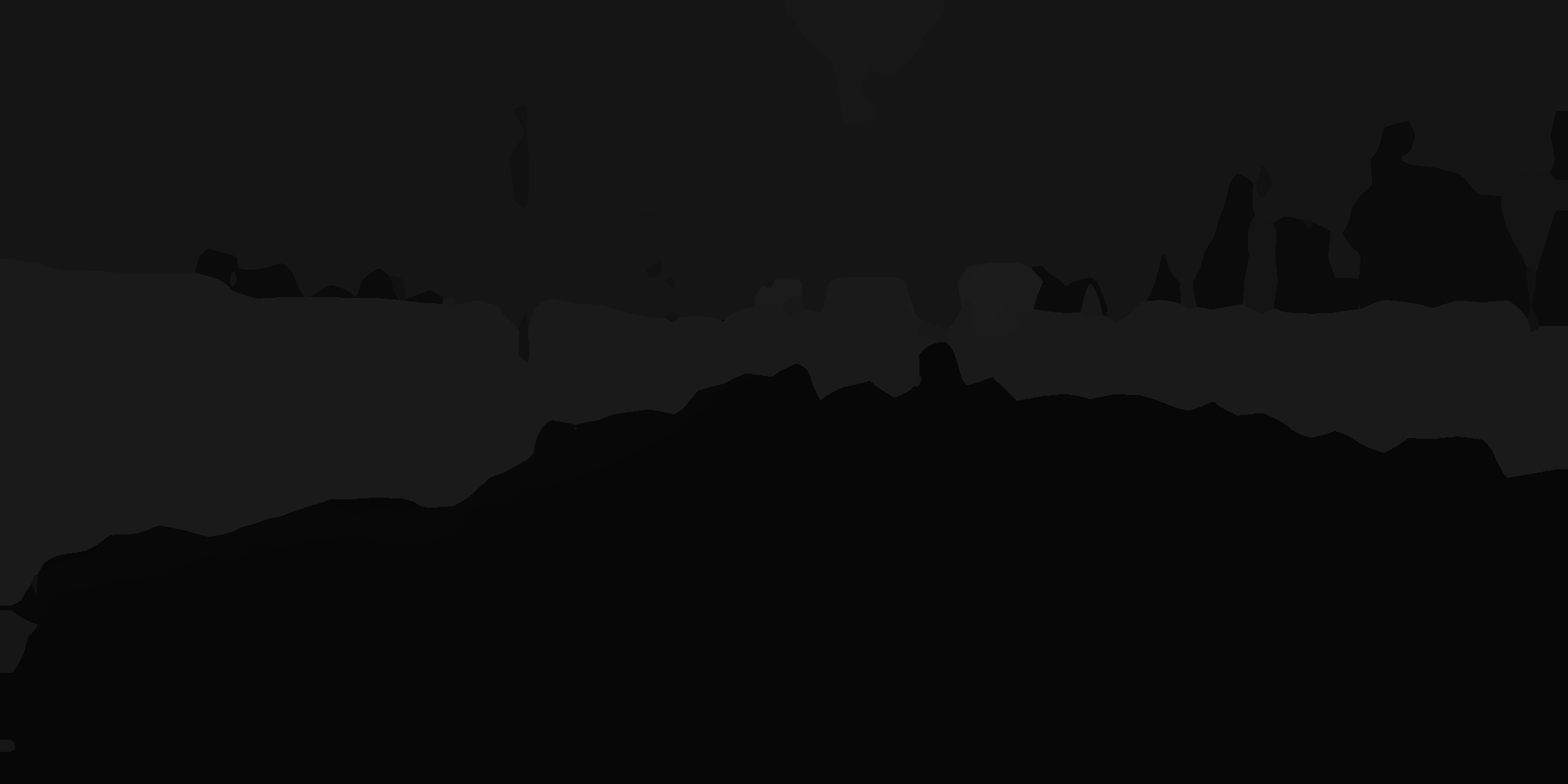

Supplement: S1 Data — (ZIP) [file pone.0295263.s001.zip › ╨┬╜¿╬─╝■╝╨ (2)/groundtruth/berlin_000019_000019_gtFine_labelIds.png]

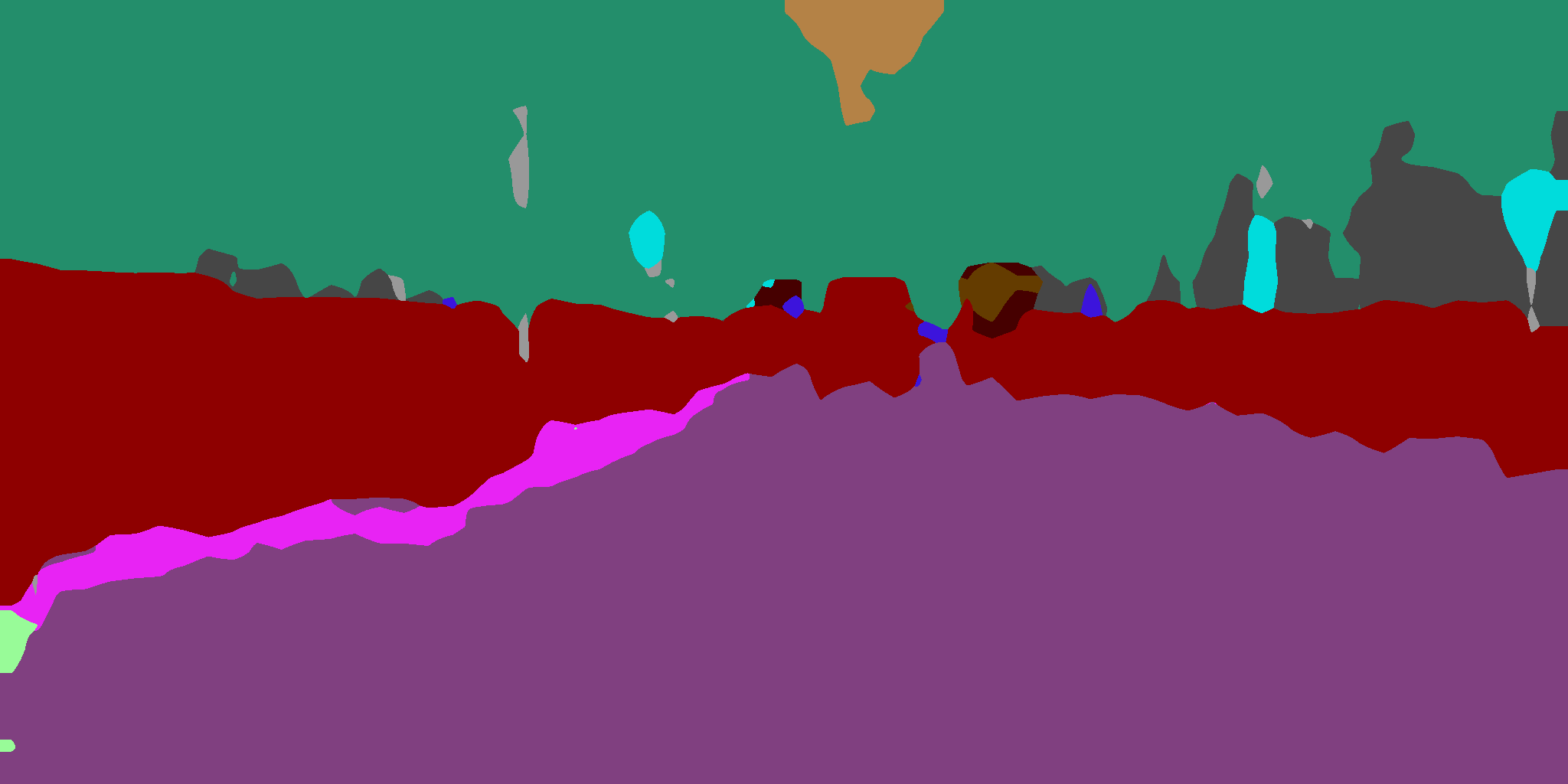

Supplement: S1 Data — (ZIP) [file pone.0295263.s001.zip › ╨┬╜¿╬─╝■╝╨ (2)/groundtruth/berlin_000019_000019_gtFine_labelTrainIds.png]

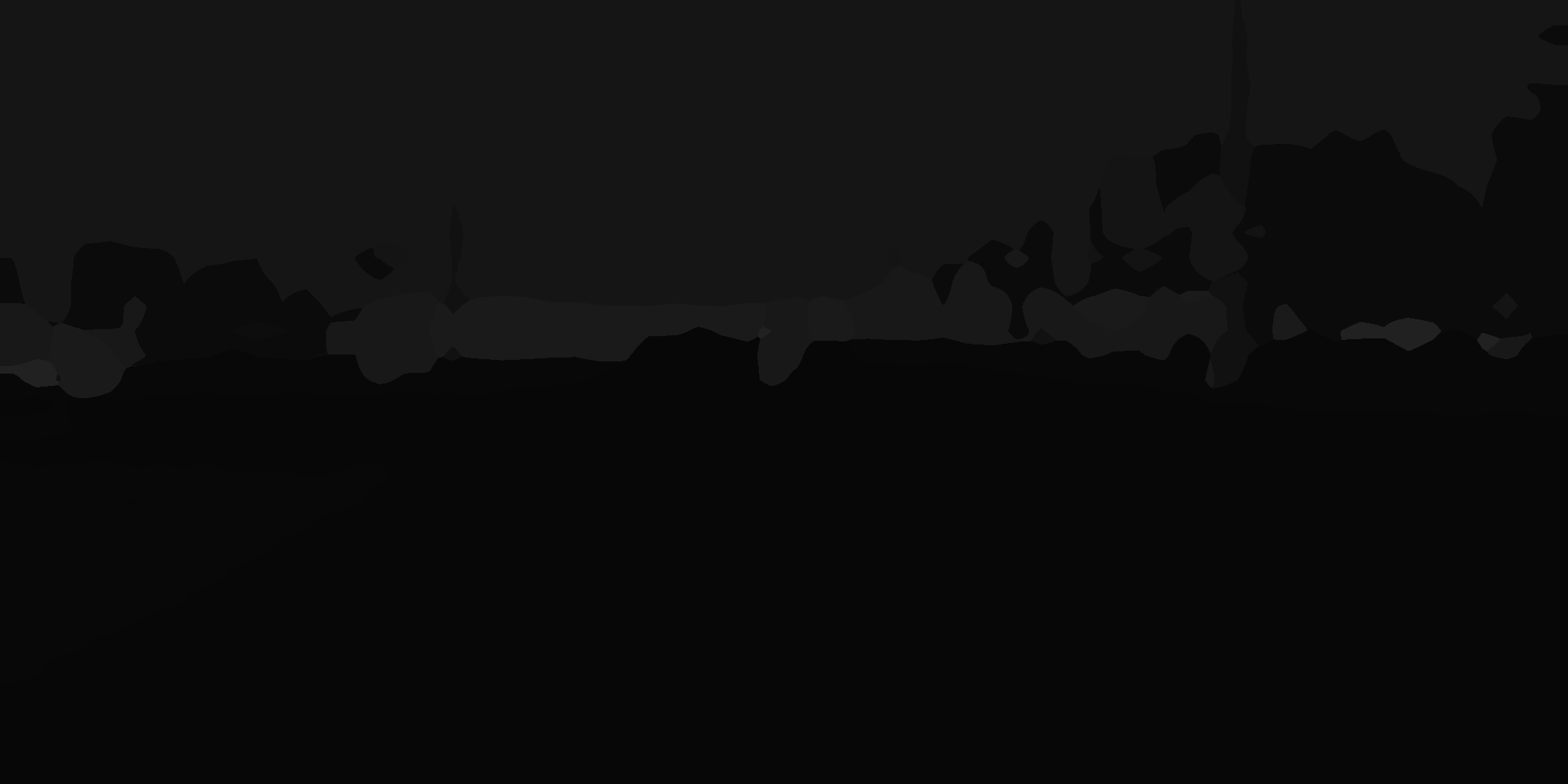

Supplement: S1 Data — (ZIP) [file pone.0295263.s001.zip › ╨┬╜¿╬─╝■╝╨ (2)/groundtruth/berlin_000020_000019_gtFine_labelIds.png]

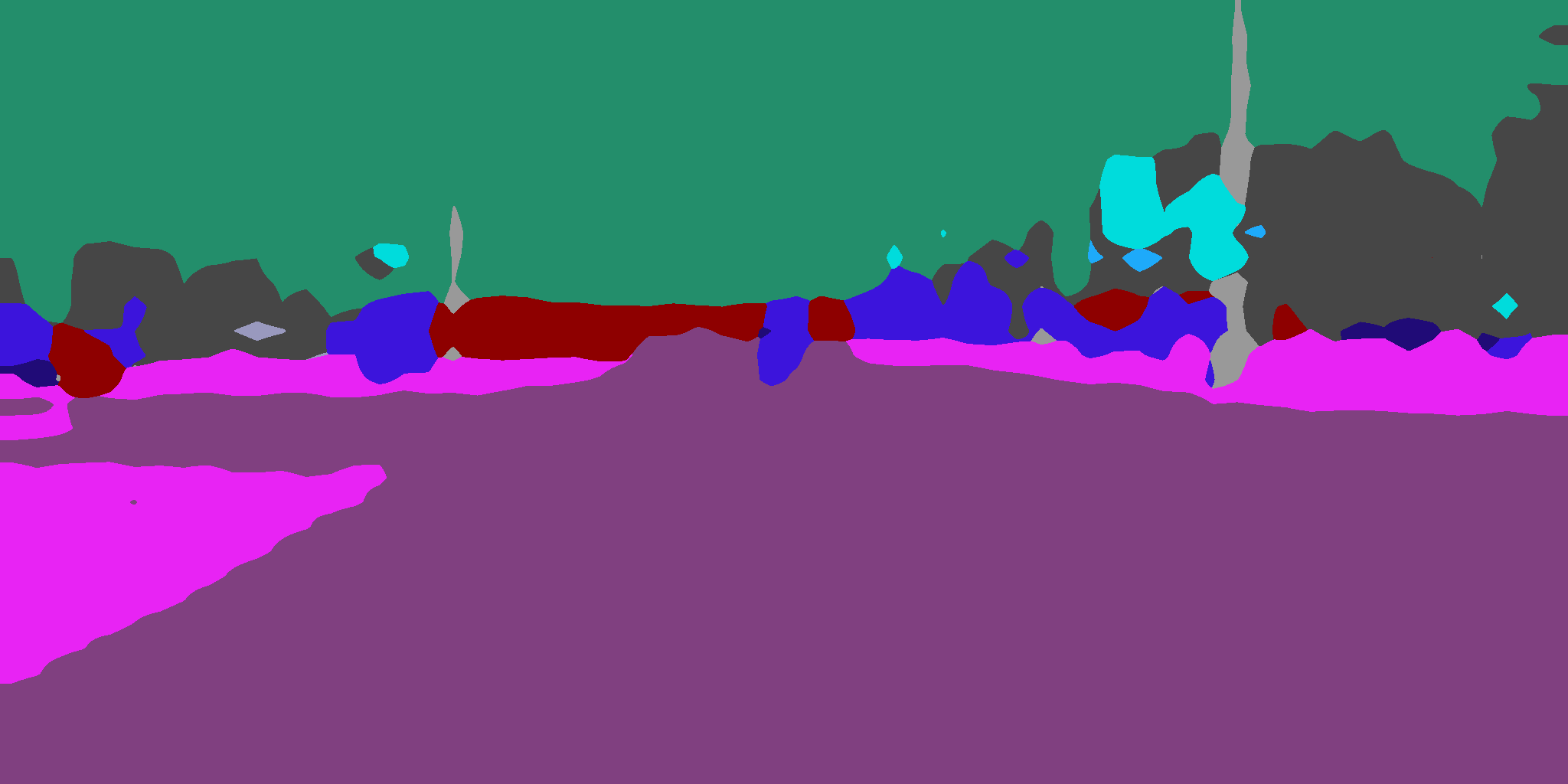

Supplement: S1 Data — (ZIP) [file pone.0295263.s001.zip › ╨┬╜¿╬─╝■╝╨ (2)/groundtruth/berlin_000020_000019_gtFine_labelTrainIds.png]

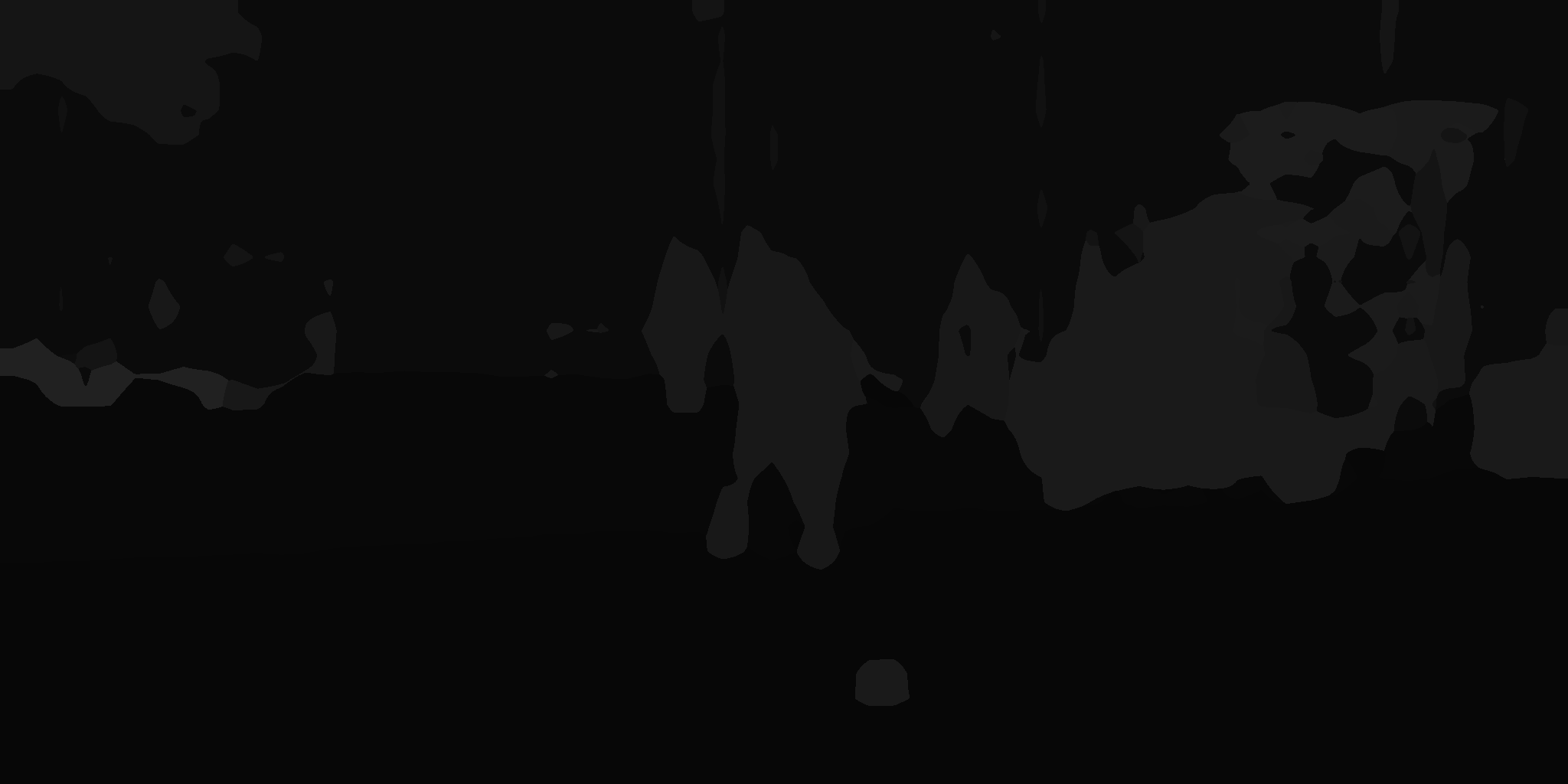

Supplement: S1 Data — (ZIP) [file pone.0295263.s001.zip › ╨┬╜¿╬─╝■╝╨ (2)/groundtruth/berlin_000021_000019_gtFine_labelIds.png]

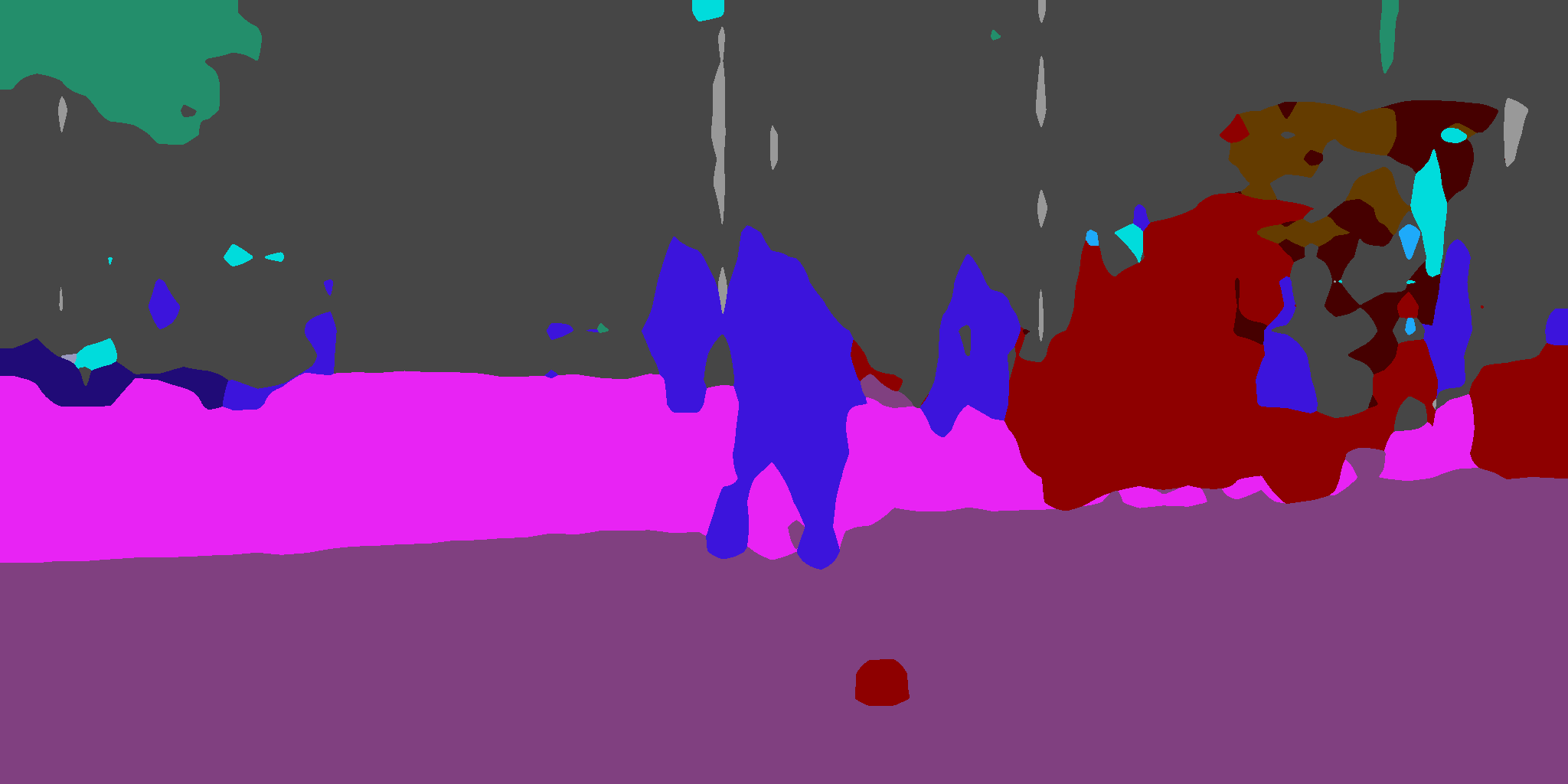

Supplement: S1 Data — (ZIP) [file pone.0295263.s001.zip › ╨┬╜¿╬─╝■╝╨ (2)/groundtruth/berlin_000021_000019_gtFine_labelTrainIds.png]

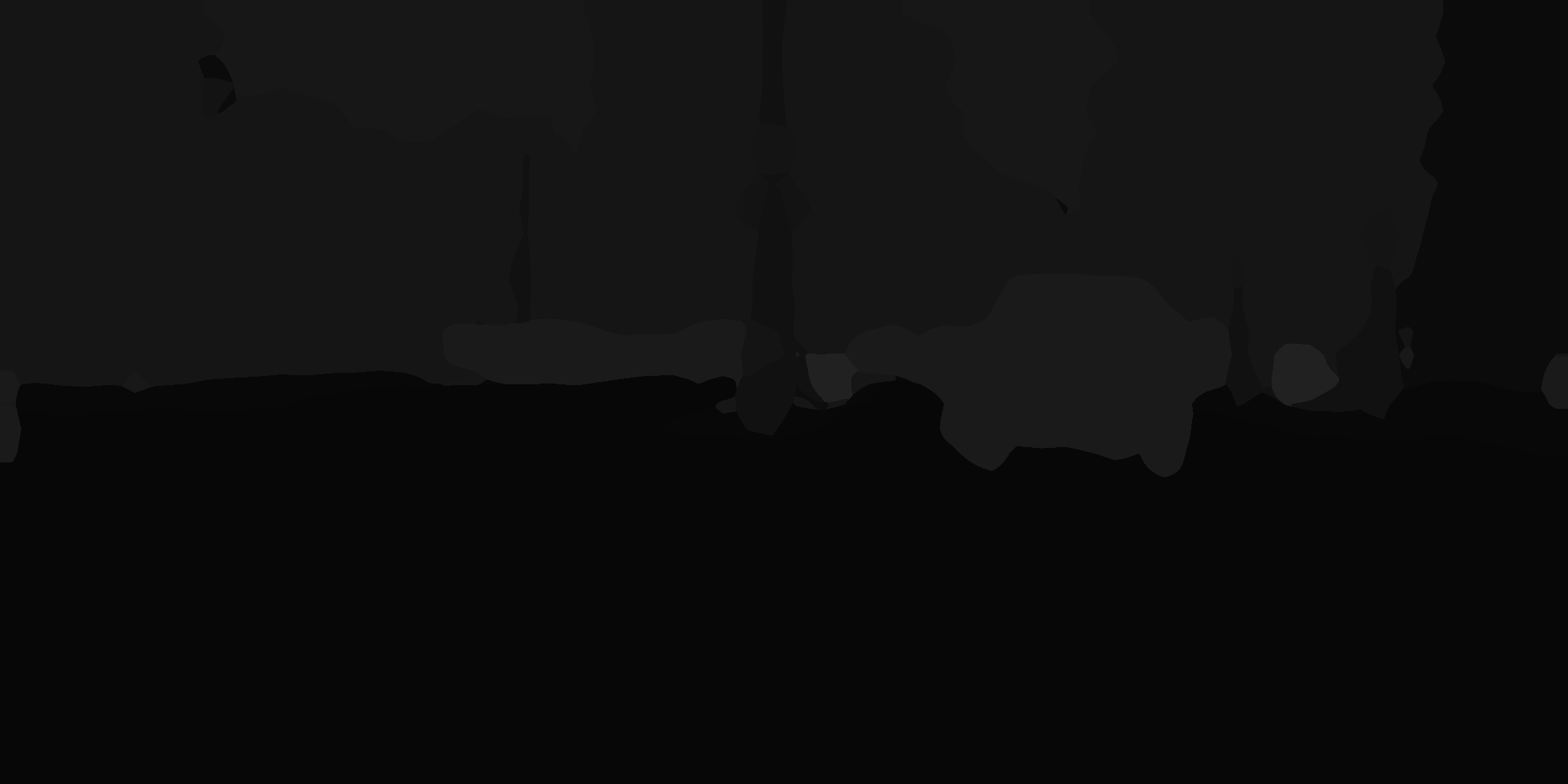

Supplement: S1 Data — (ZIP) [file pone.0295263.s001.zip › ╨┬╜¿╬─╝■╝╨ (2)/groundtruth/berlin_000022_000019_gtFine_labelIds.png]

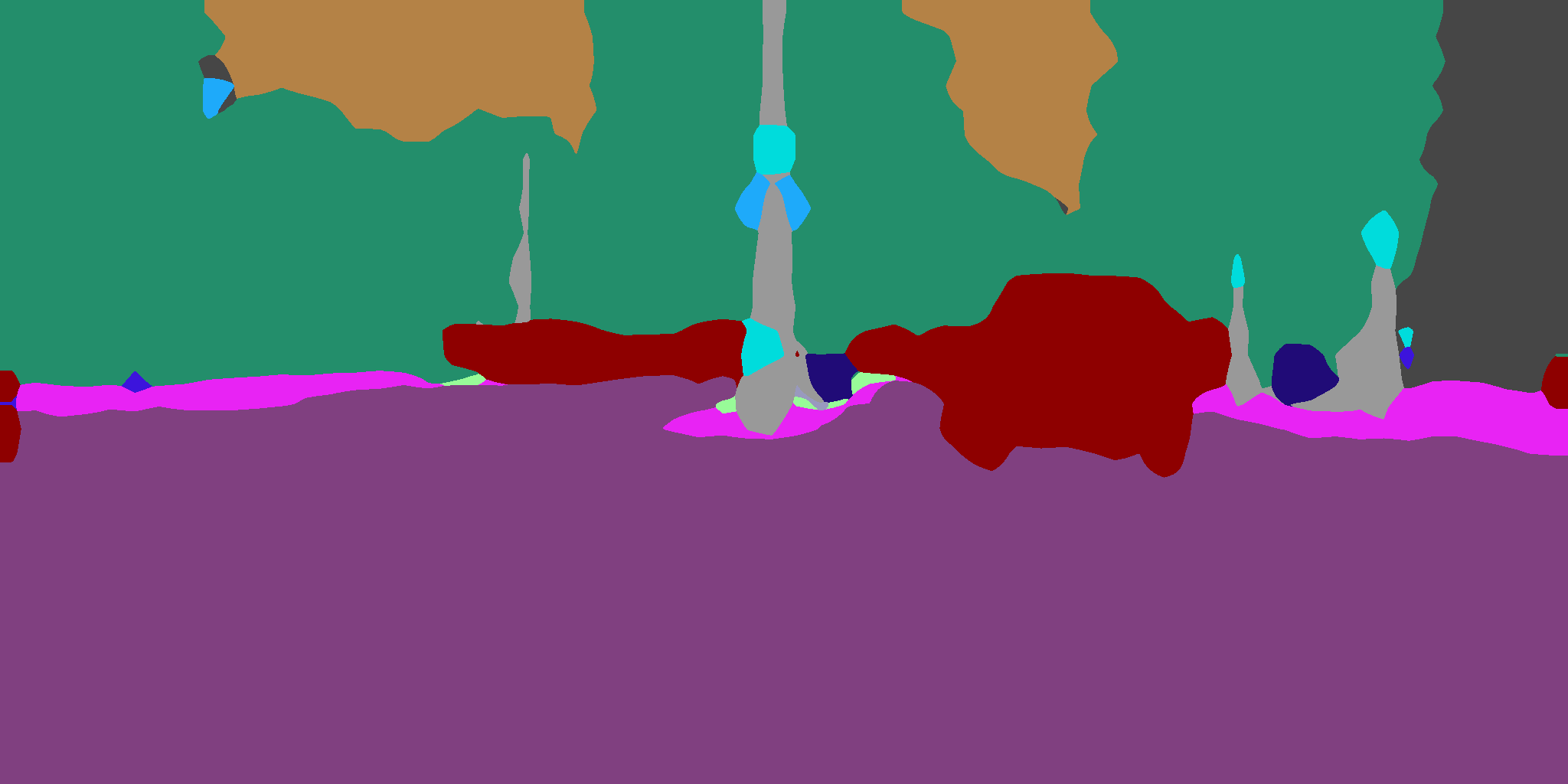

Supplement: S1 Data — (ZIP) [file pone.0295263.s001.zip › ╨┬╜¿╬─╝■╝╨ (2)/groundtruth/berlin_000022_000019_gtFine_labelTrainIds.png]

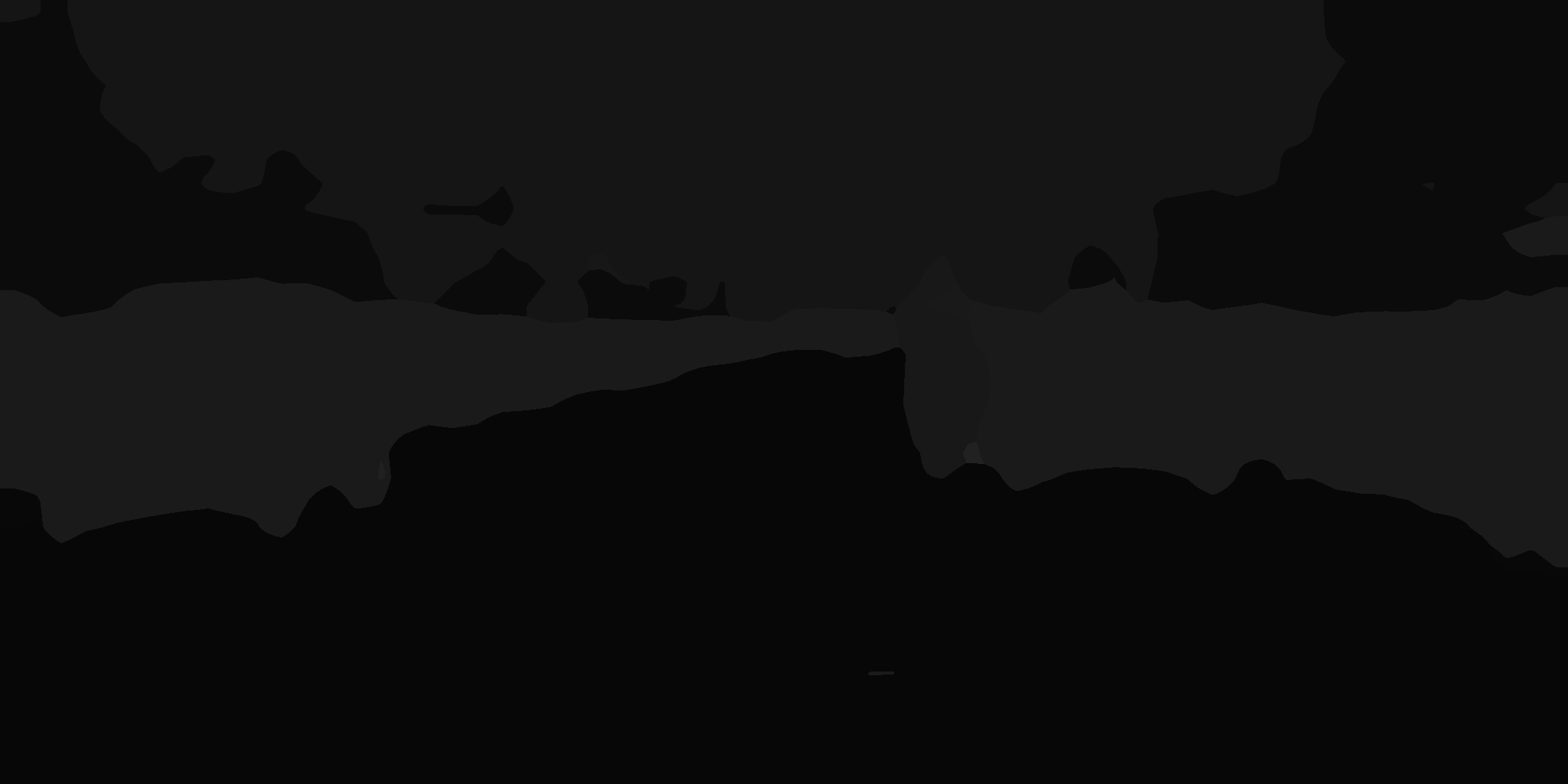

Supplement: S1 Data — (ZIP) [file pone.0295263.s001.zip › ╨┬╜¿╬─╝■╝╨ (2)/groundtruth/berlin_000023_000019_gtFine_labelIds.png]

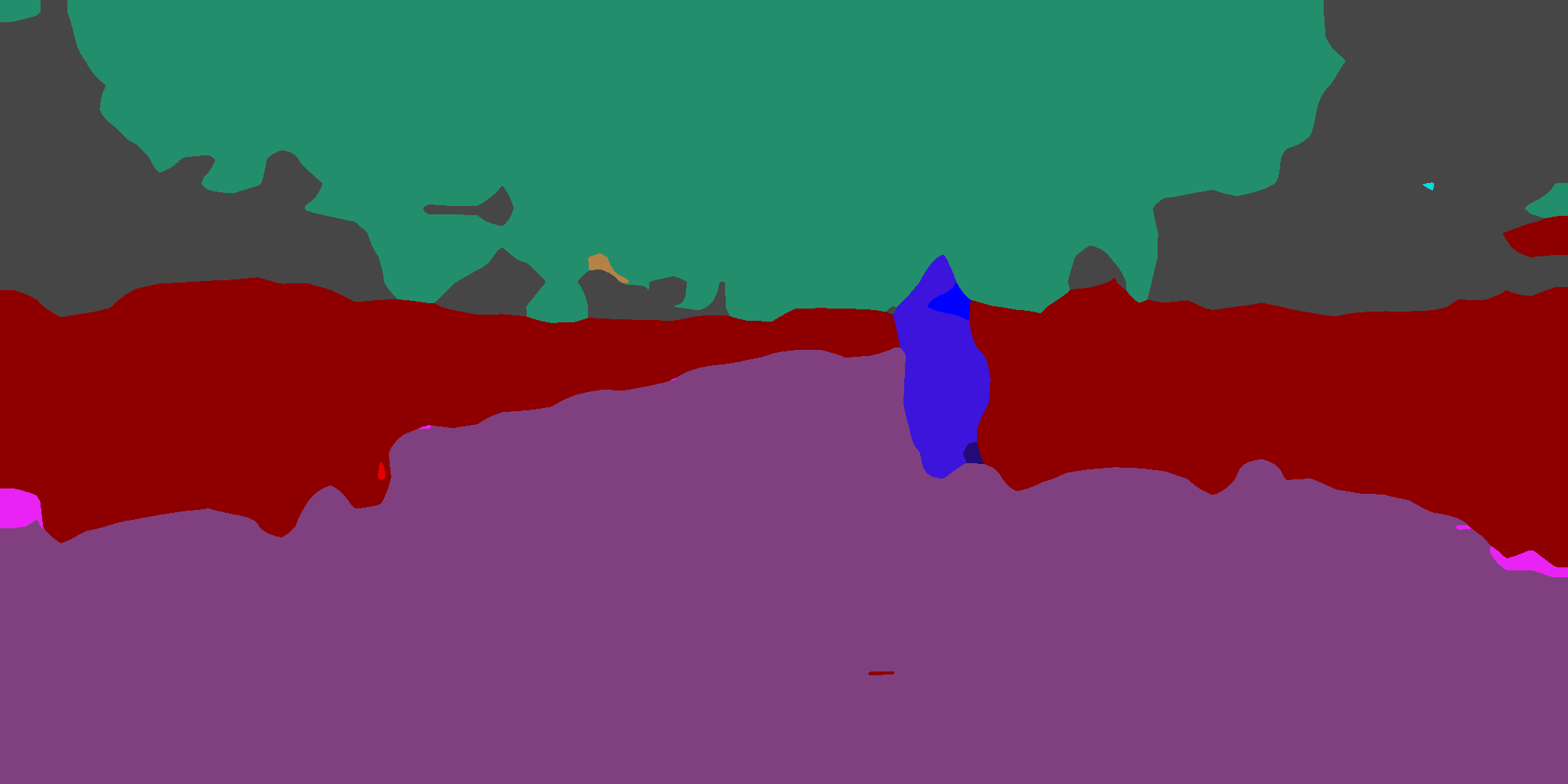

Supplement: S1 Data — (ZIP) [file pone.0295263.s001.zip › ╨┬╜¿╬─╝■╝╨ (2)/groundtruth/berlin_000023_000019_gtFine_labelTrainIds.png]

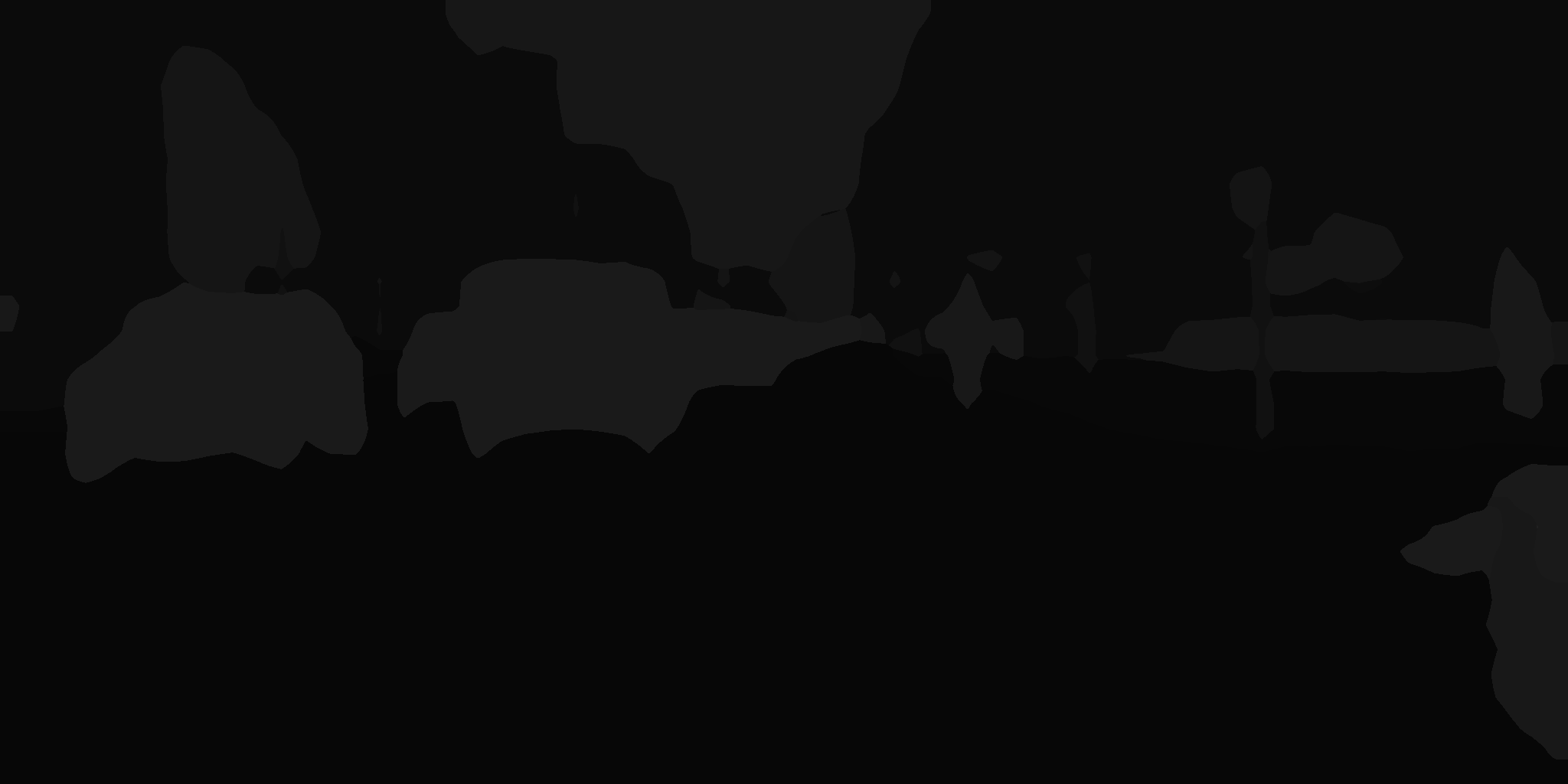

Supplement: S1 Data — (ZIP) [file pone.0295263.s001.zip › ╨┬╜¿╬─╝■╝╨ (2)/groundtruth/berlin_000024_000019_gtFine_labelIds.png]

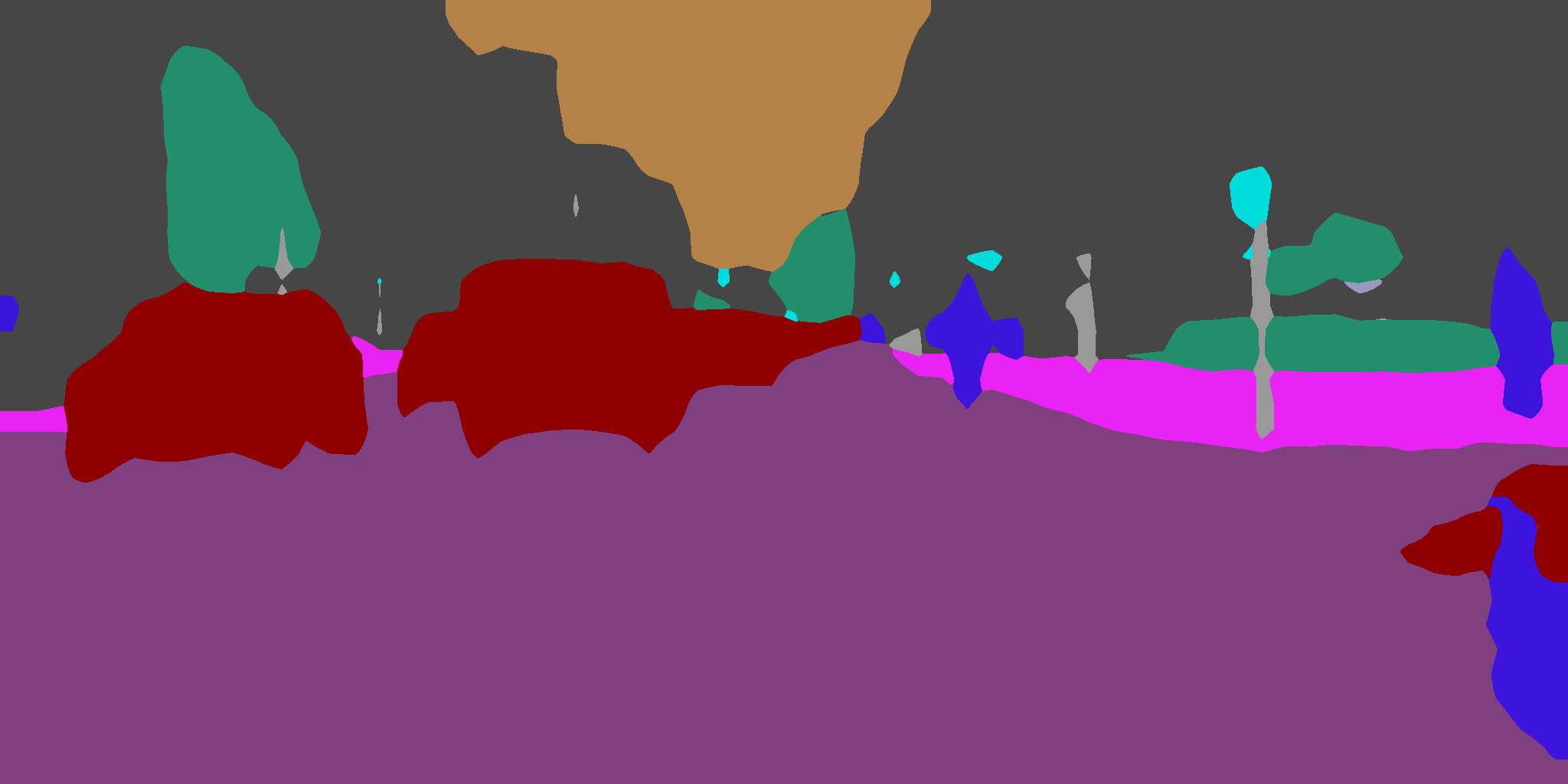

Supplement: S1 Data — (ZIP) [file pone.0295263.s001.zip › ╨┬╜¿╬─╝■╝╨ (2)/groundtruth/berlin_000024_000019_gtFine_labelTrainIds.png]

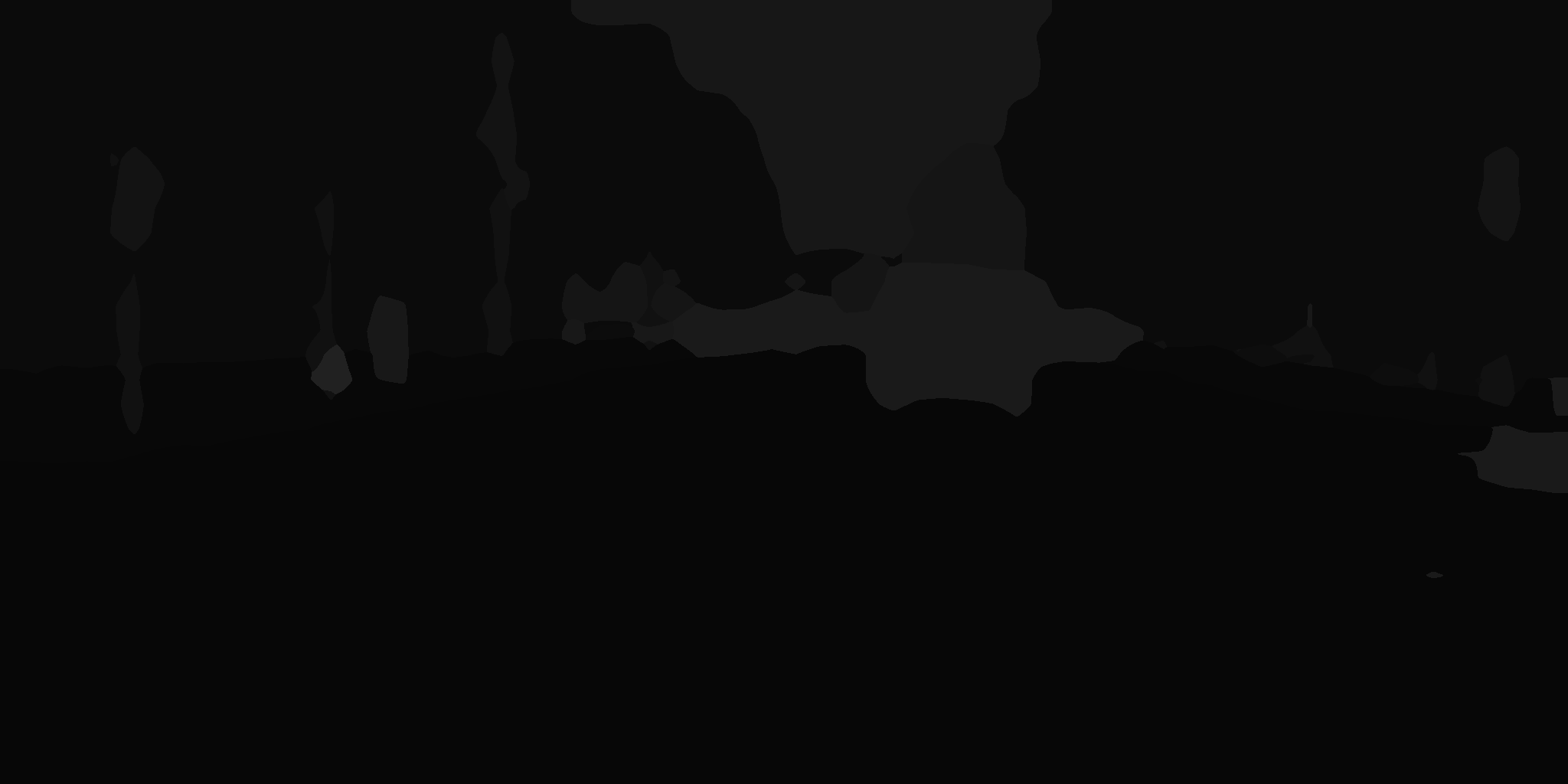

Supplement: S1 Data — (ZIP) [file pone.0295263.s001.zip › ╨┬╜¿╬─╝■╝╨ (2)/groundtruth/berlin_000025_000019_gtFine_labelIds.png]

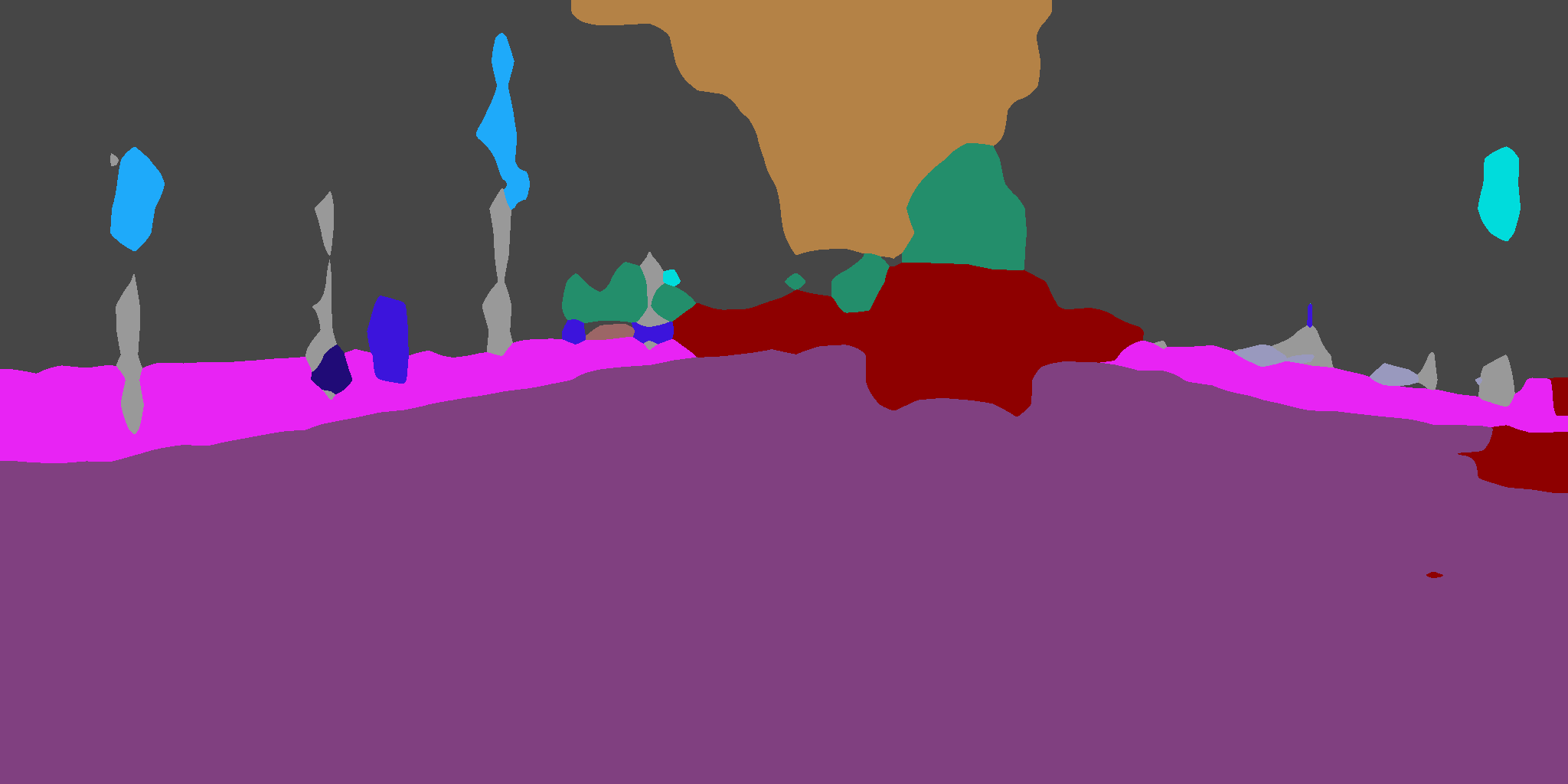

Supplement: S1 Data — (ZIP) [file pone.0295263.s001.zip › ╨┬╜¿╬─╝■╝╨ (2)/groundtruth/berlin_000025_000019_gtFine_labelTrainIds.png]

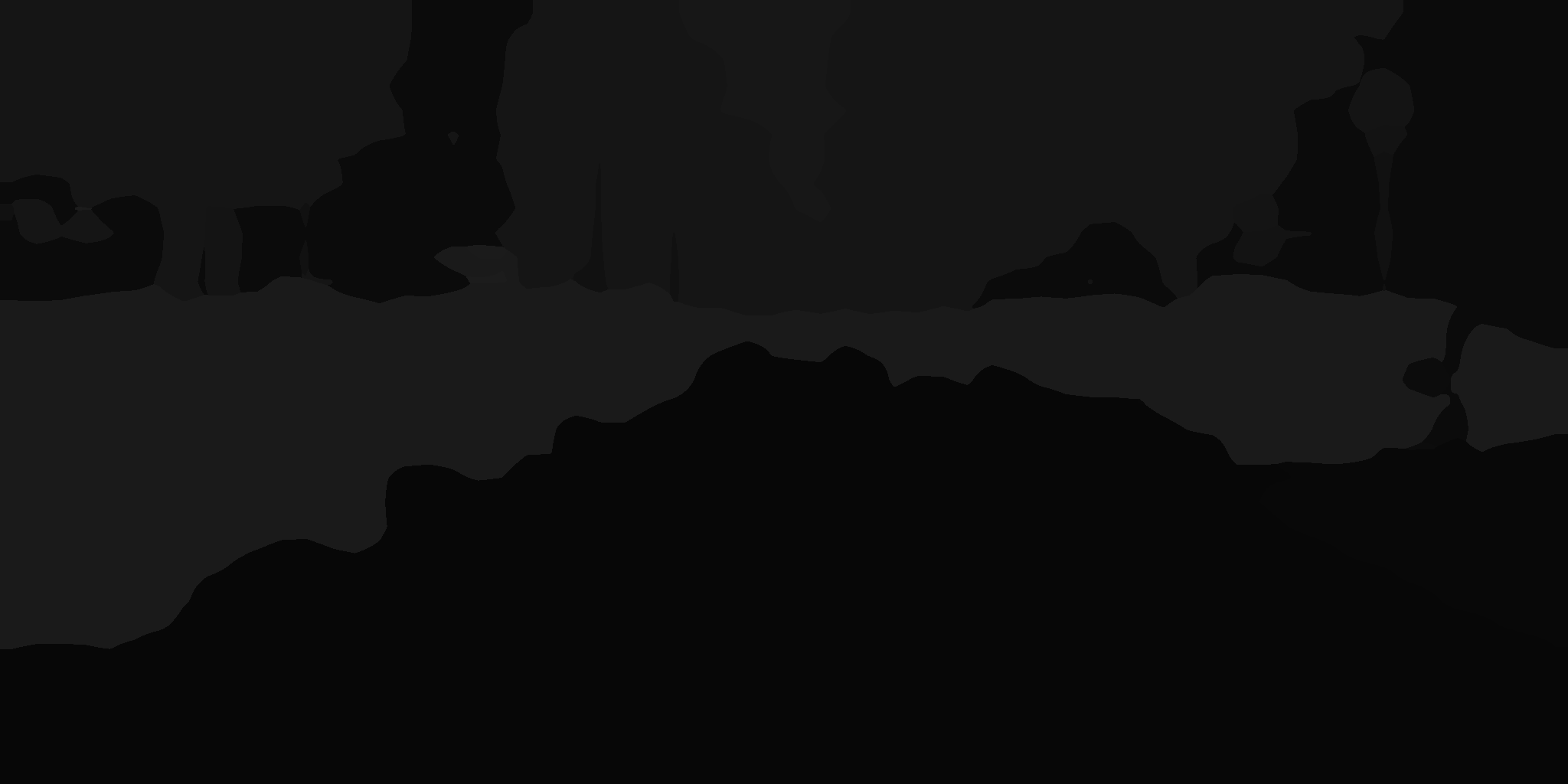

Supplement: S1 Data — (ZIP) [file pone.0295263.s001.zip › ╨┬╜¿╬─╝■╝╨ (2)/groundtruth/berlin_000026_000019_gtFine_labelIds.png]

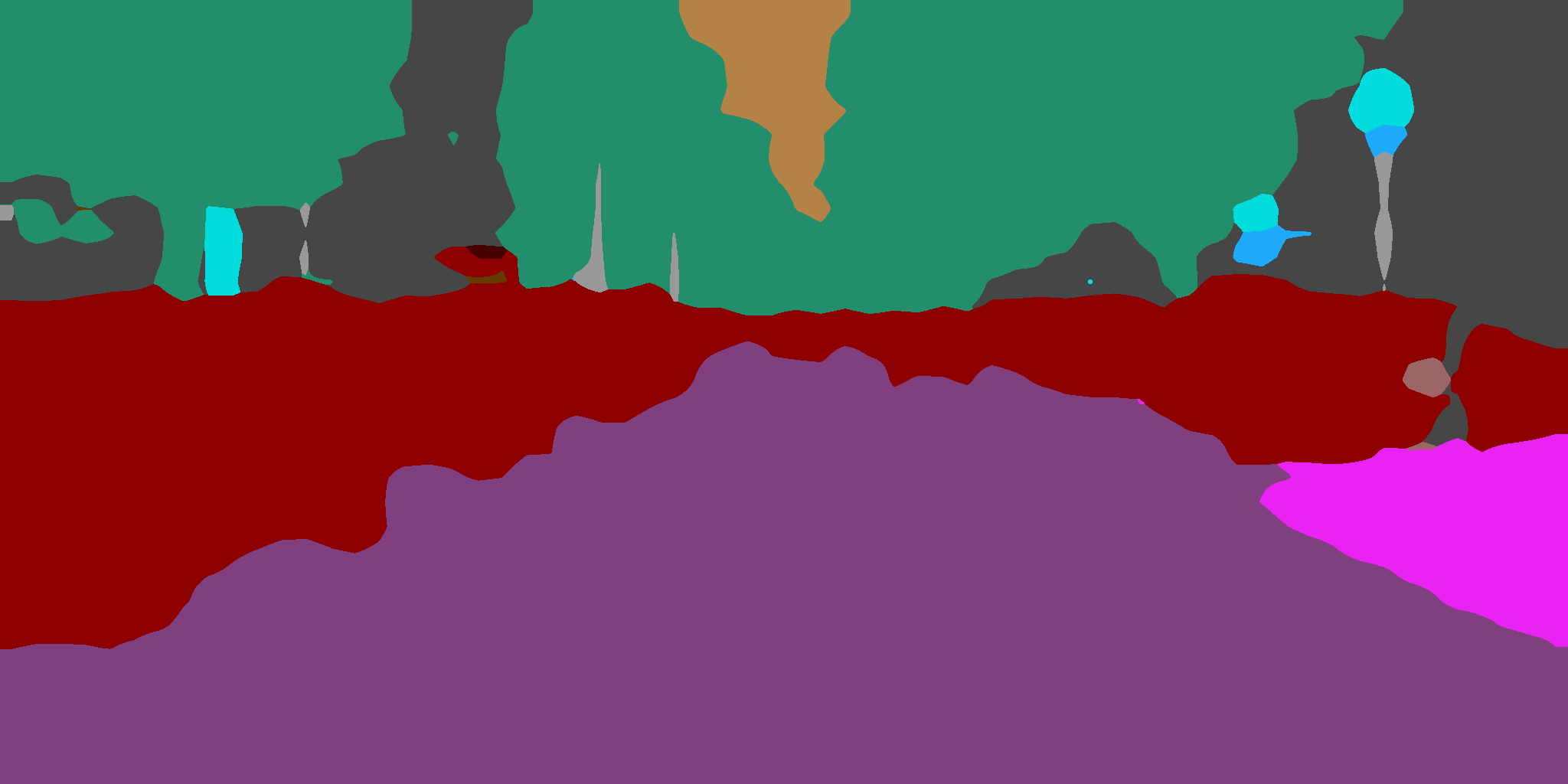

Supplement: S1 Data — (ZIP) [file pone.0295263.s001.zip › ╨┬╜¿╬─╝■╝╨ (2)/groundtruth/berlin_000026_000019_gtFine_labelTrainIds.png]

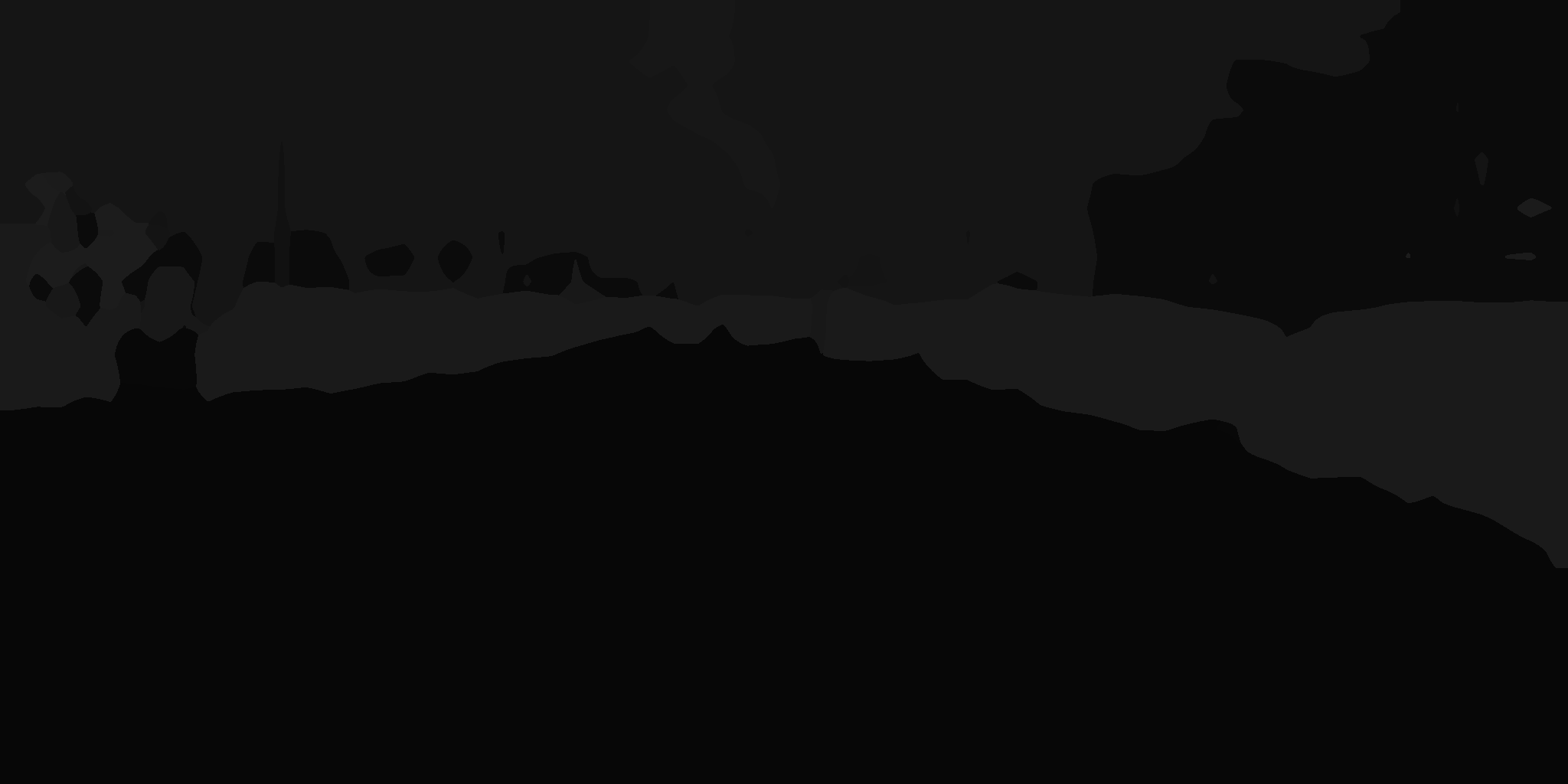

Supplement: S1 Data — (ZIP) [file pone.0295263.s001.zip › ╨┬╜¿╬─╝■╝╨ (2)/groundtruth/berlin_000027_000019_gtFine_labelIds.png]

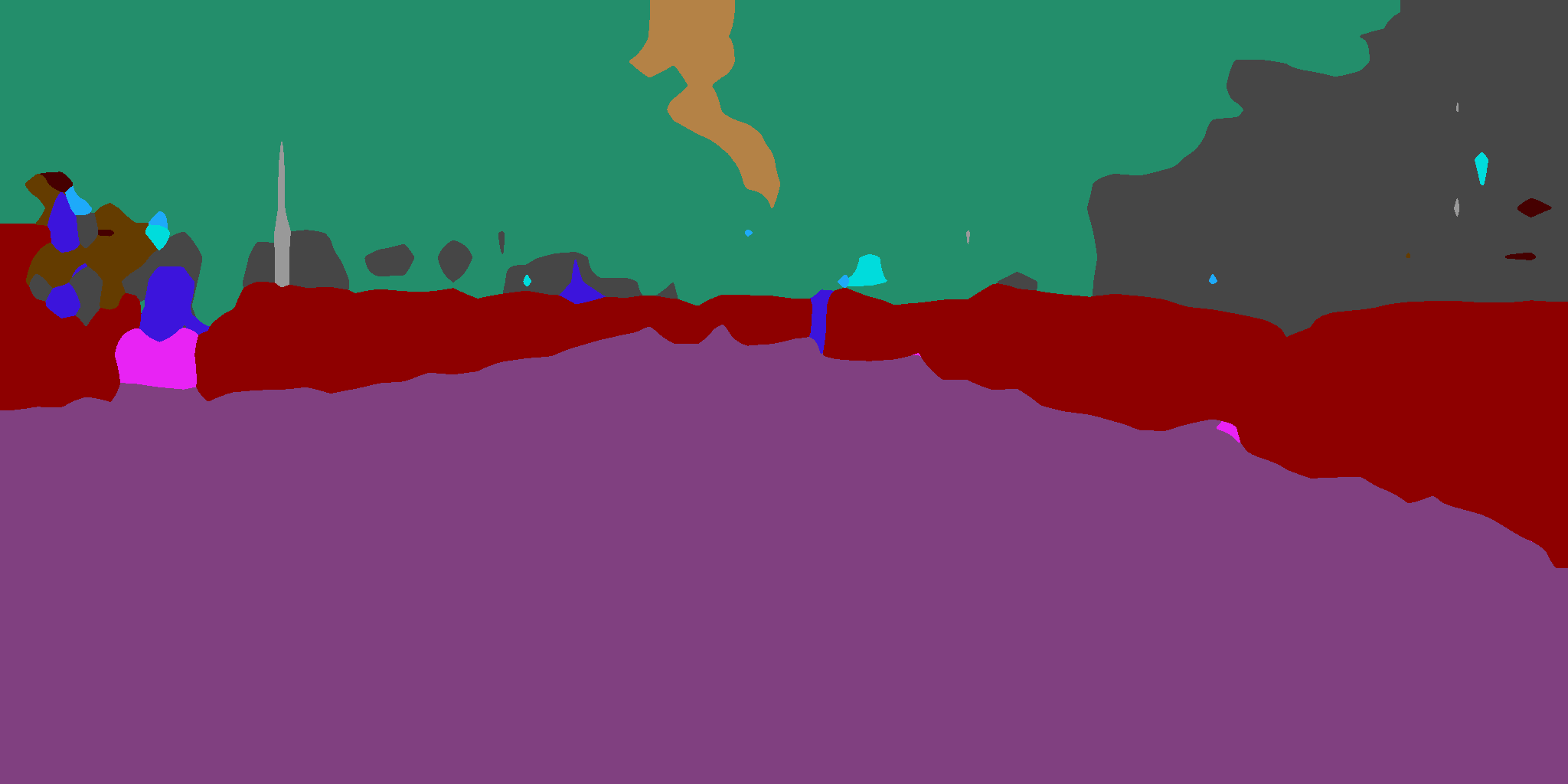

Supplement: S1 Data — (ZIP) [file pone.0295263.s001.zip › ╨┬╜¿╬─╝■╝╨ (2)/groundtruth/berlin_000027_000019_gtFine_labelTrainIds.png]

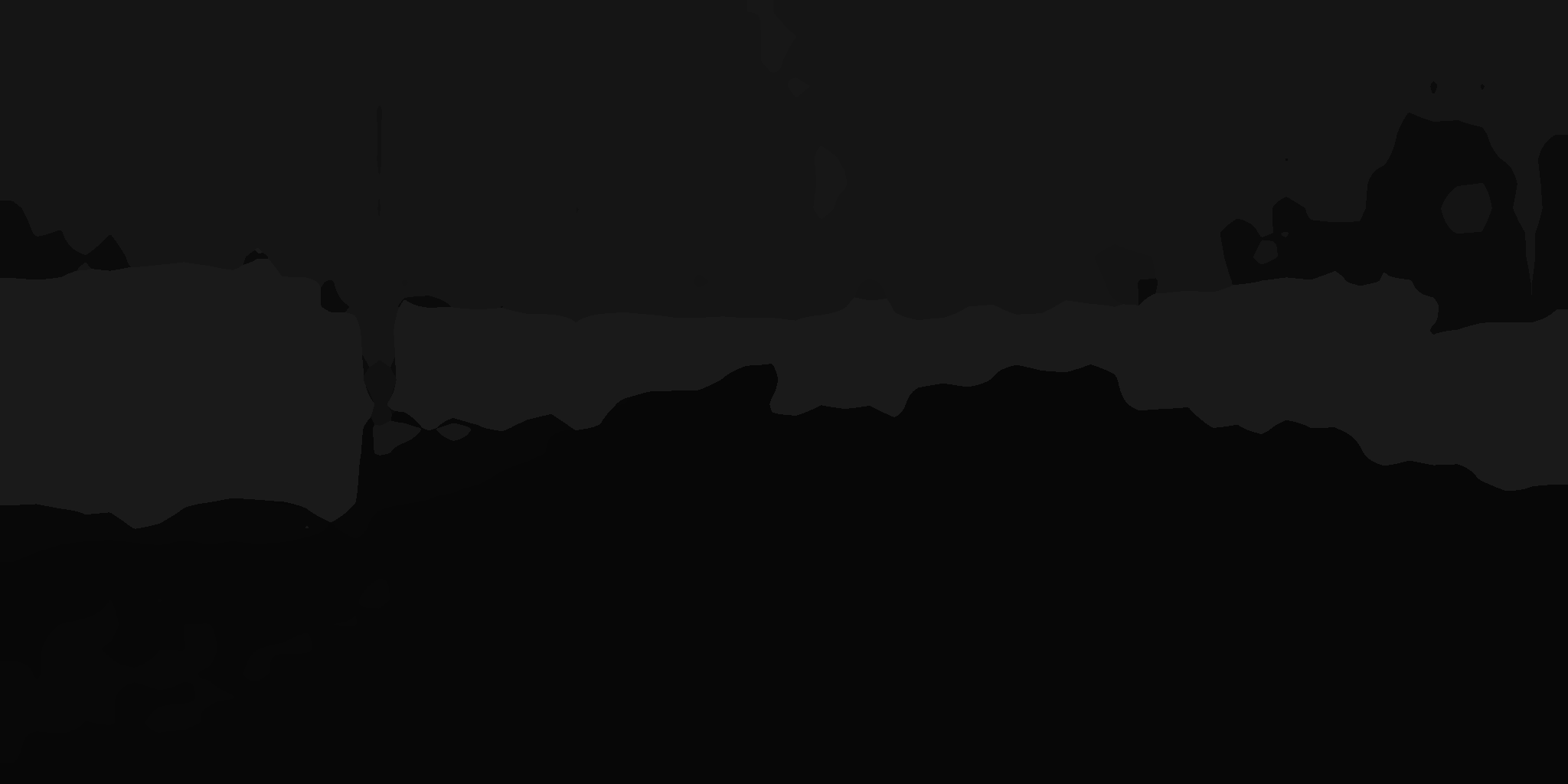

Supplement: S1 Data — (ZIP) [file pone.0295263.s001.zip › ╨┬╜¿╬─╝■╝╨ (2)/groundtruth/berlin_000028_000019_gtFine_labelIds.png]

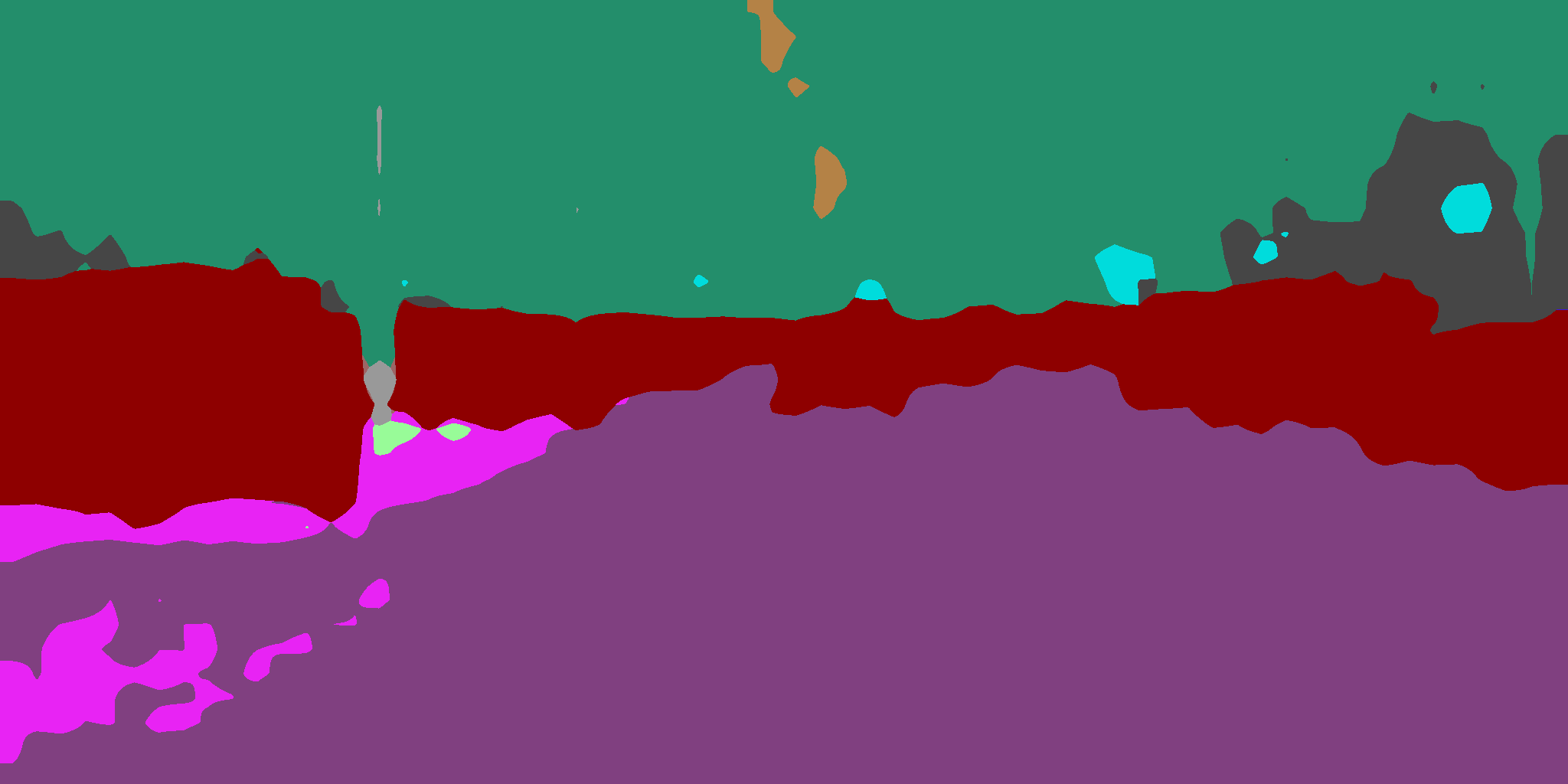

Supplement: S1 Data — (ZIP) [file pone.0295263.s001.zip › ╨┬╜¿╬─╝■╝╨ (2)/groundtruth/berlin_000028_000019_gtFine_labelTrainIds.png]

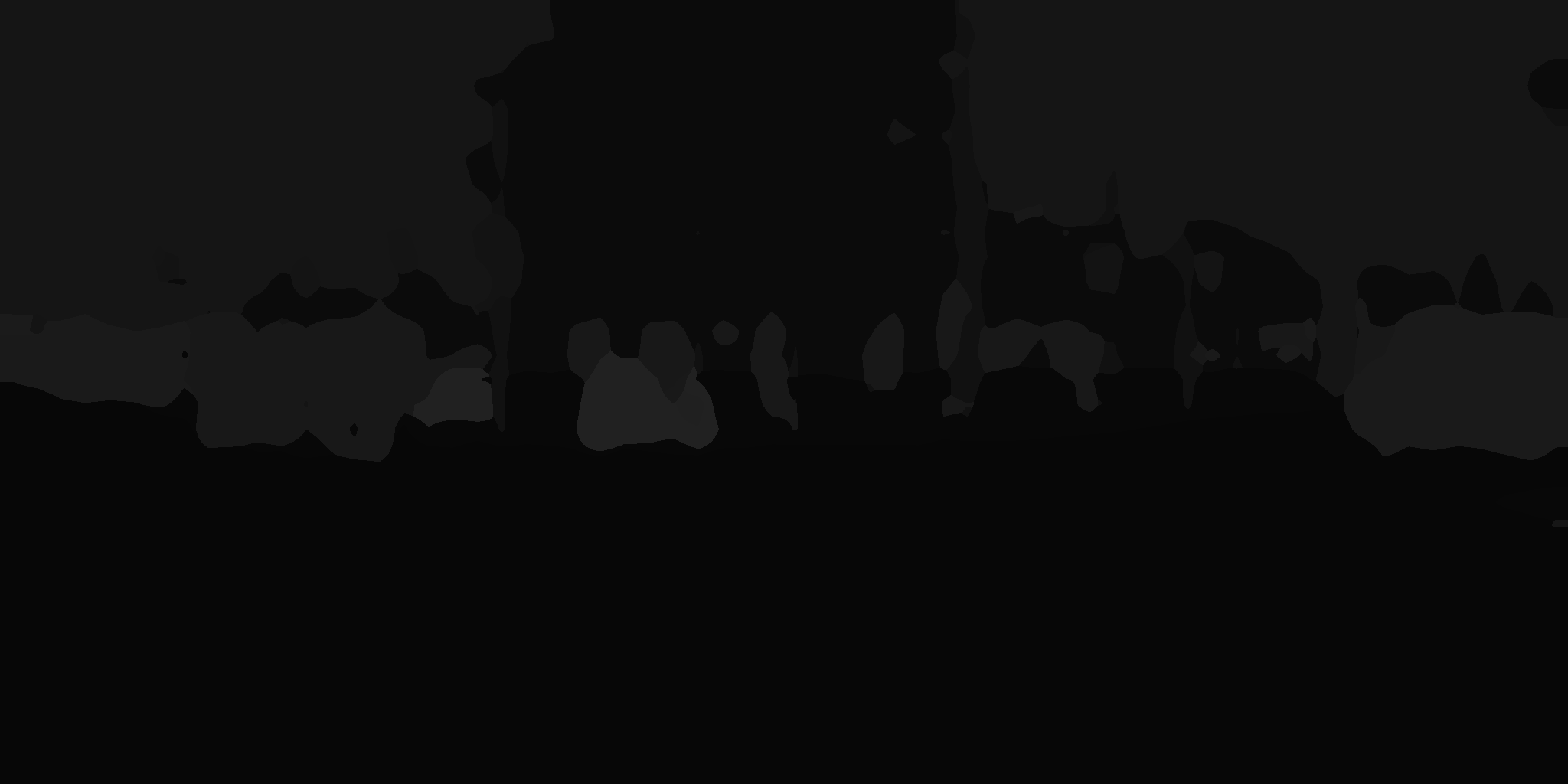

Supplement: S1 Data — (ZIP) [file pone.0295263.s001.zip › ╨┬╜¿╬─╝■╝╨ (2)/groundtruth/berlin_000029_000019_gtFine_labelIds.png]

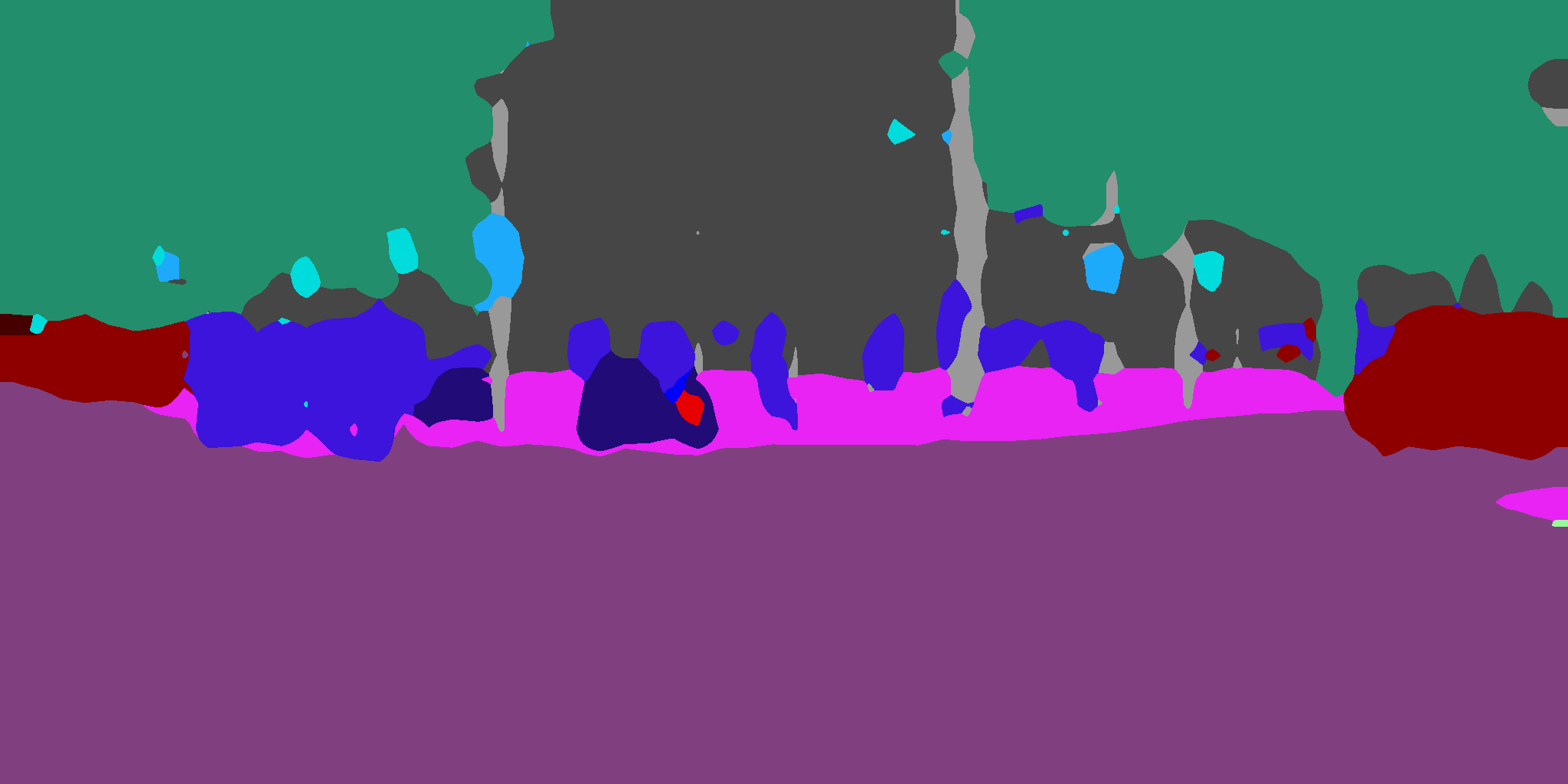

Supplement: S1 Data — (ZIP) [file pone.0295263.s001.zip › ╨┬╜¿╬─╝■╝╨ (2)/groundtruth/berlin_000029_000019_gtFine_labelTrainIds.png]

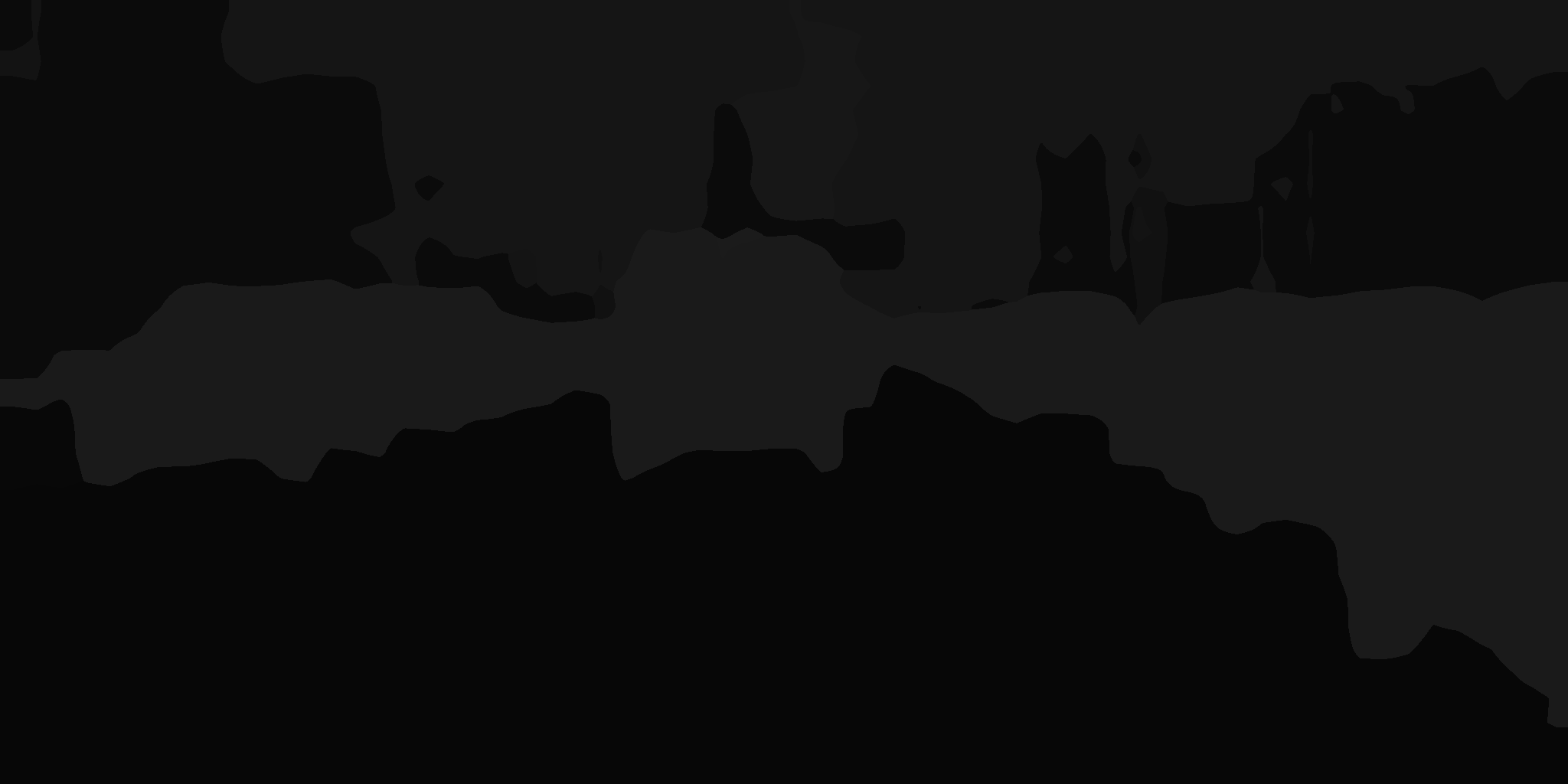

Supplement: S1 Data — (ZIP) [file pone.0295263.s001.zip › ╨┬╜¿╬─╝■╝╨ (2)/groundtruth/berlin_000030_000019_gtFine_labelIds.png]

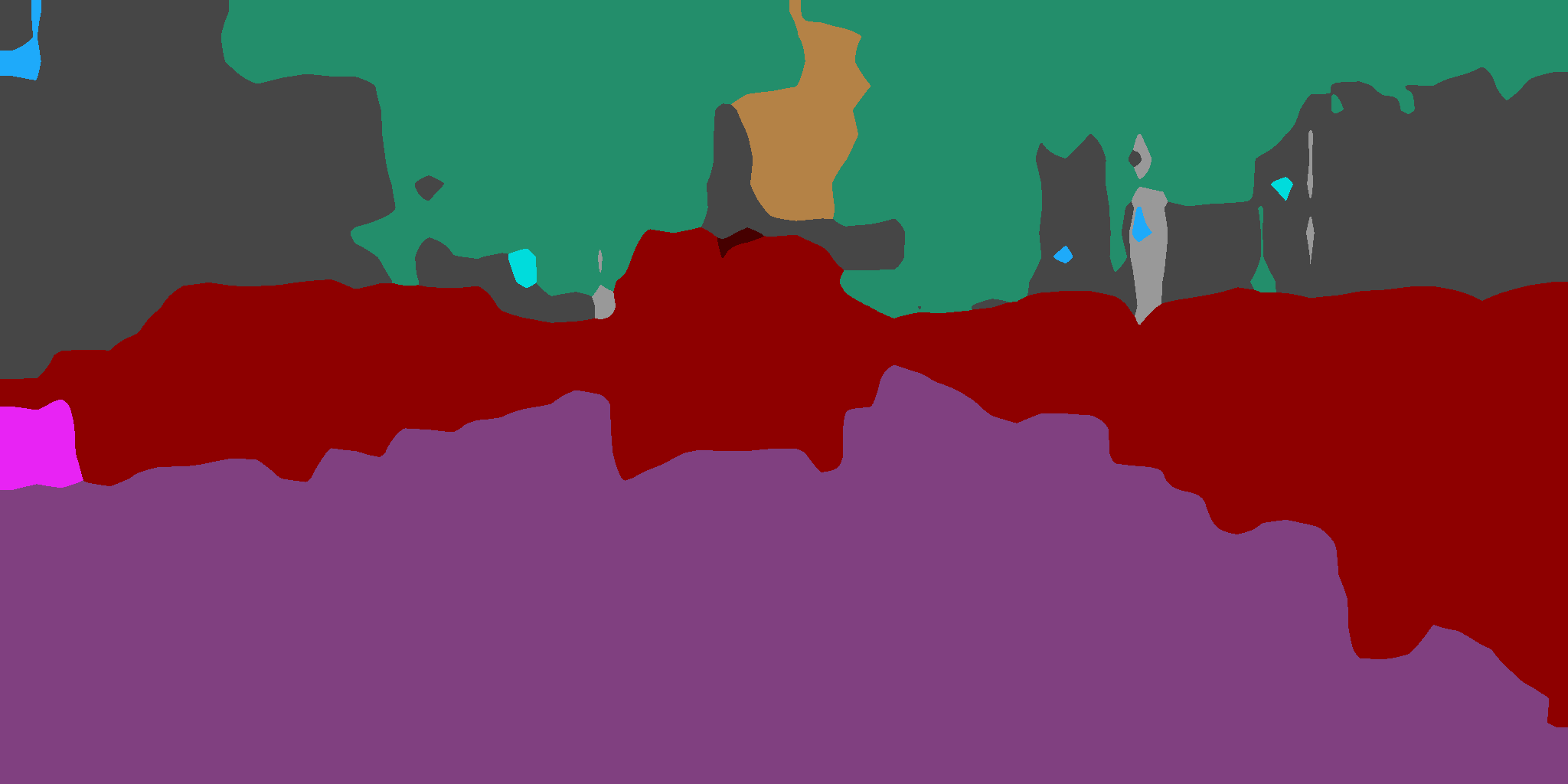

Supplement: S1 Data — (ZIP) [file pone.0295263.s001.zip › ╨┬╜¿╬─╝■╝╨ (2)/groundtruth/berlin_000030_000019_gtFine_labelTrainIds.png]

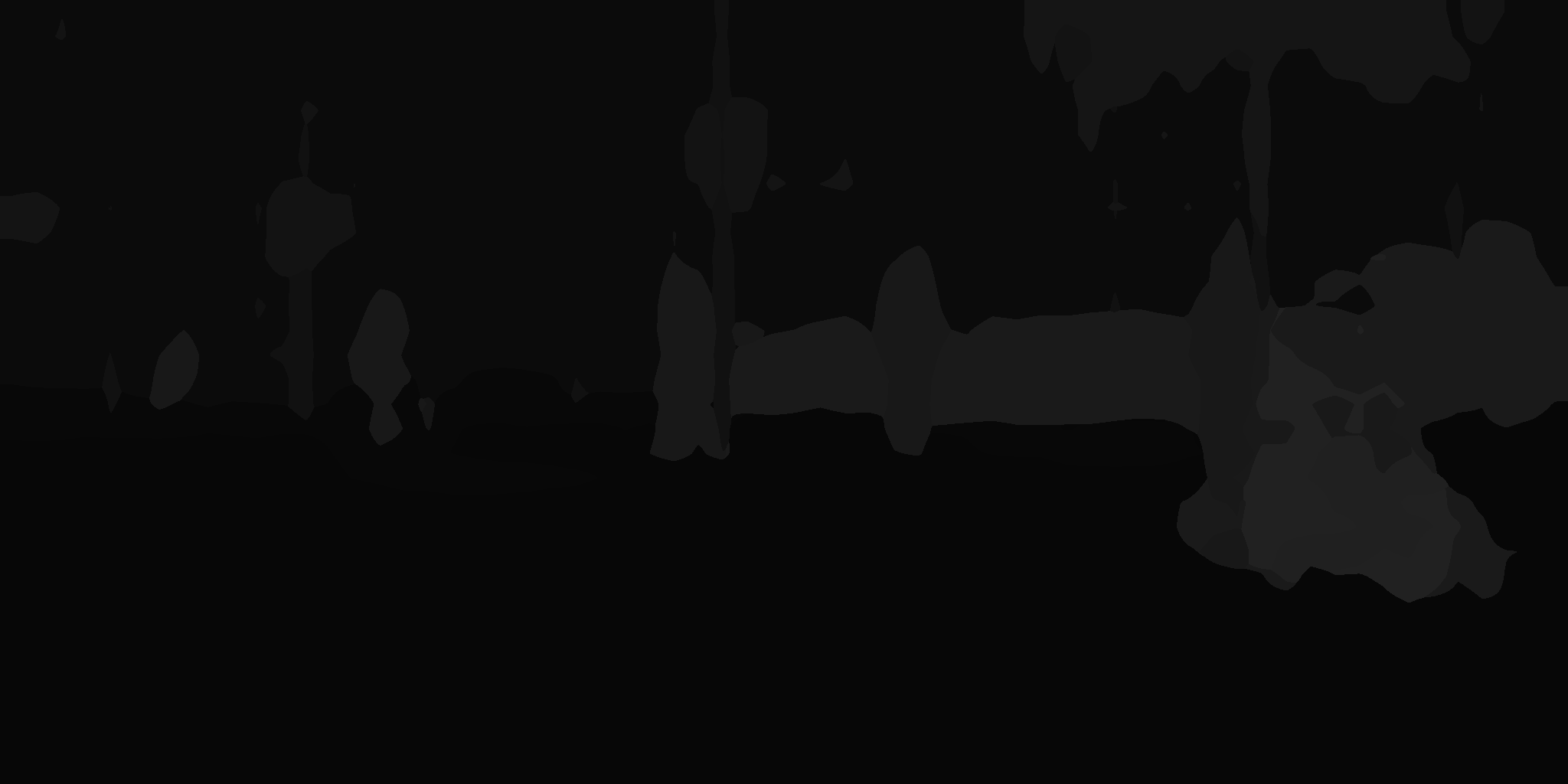

Supplement: S1 Data — (ZIP) [file pone.0295263.s001.zip › ╨┬╜¿╬─╝■╝╨ (2)/groundtruth/berlin_000031_000019_gtFine_labelIds.png]

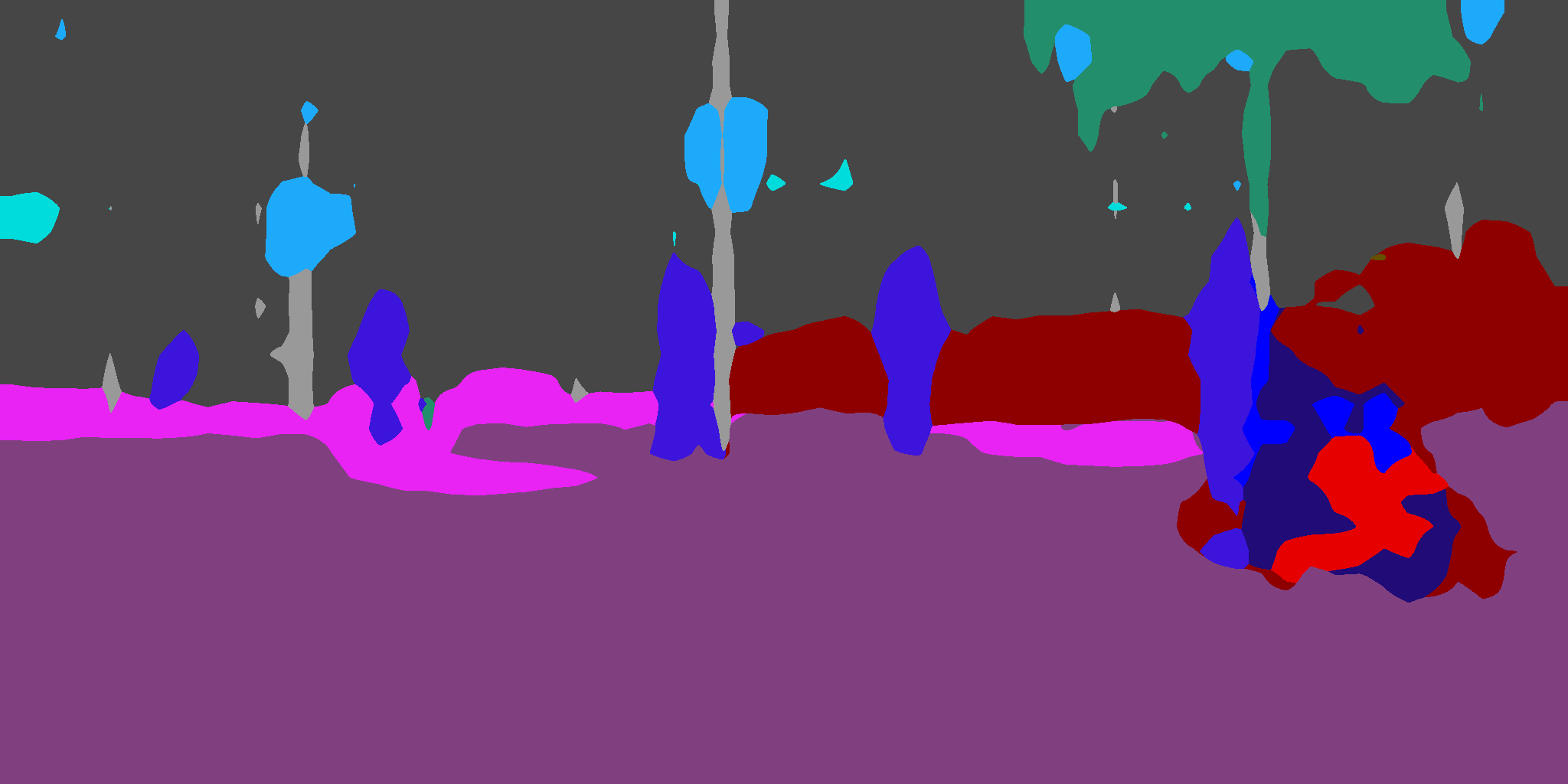

Supplement: S1 Data — (ZIP) [file pone.0295263.s001.zip › ╨┬╜¿╬─╝■╝╨ (2)/groundtruth/berlin_000031_000019_gtFine_labelTrainIds.png]

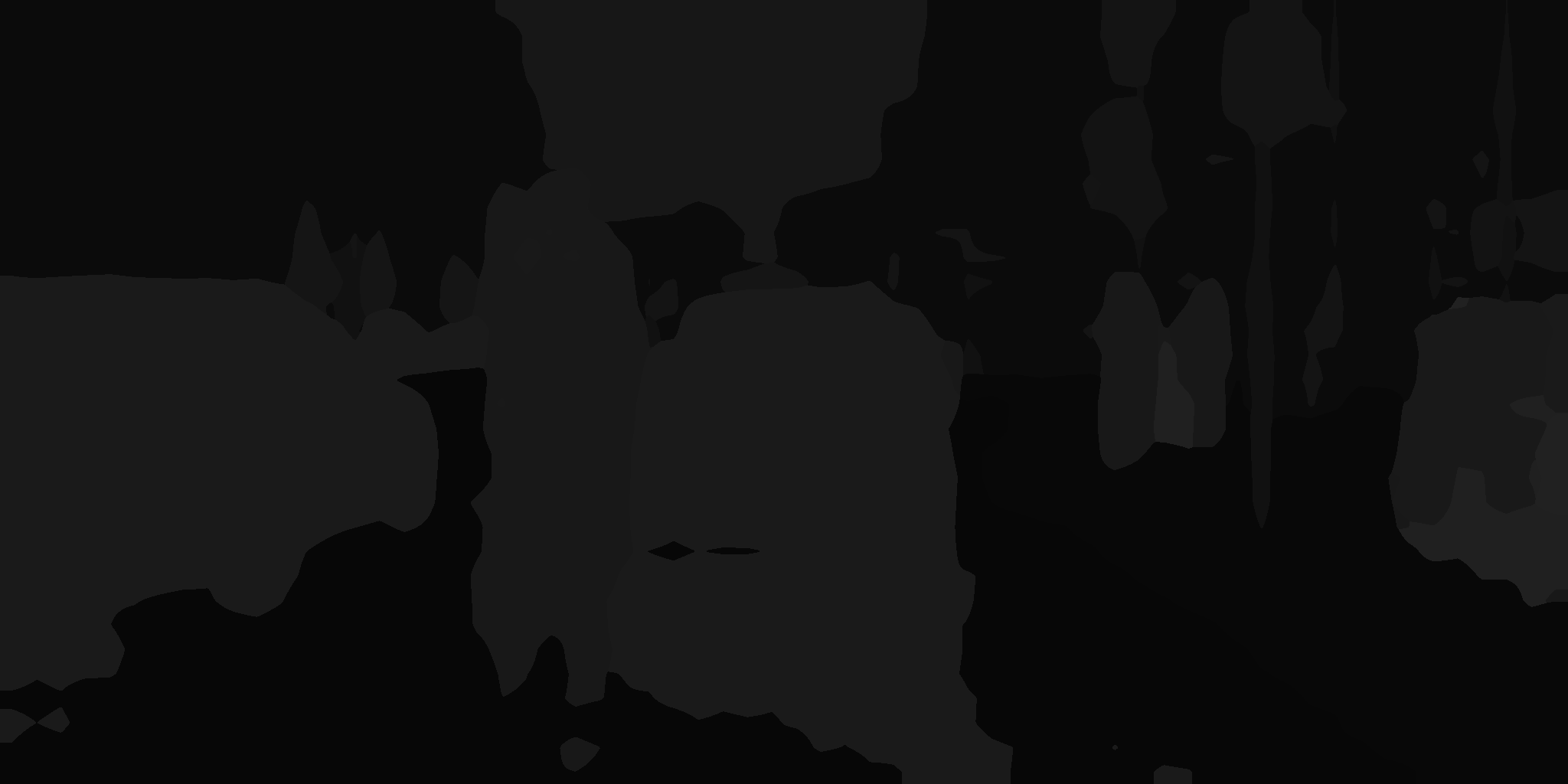

Supplement: S1 Data — (ZIP) [file pone.0295263.s001.zip › ╨┬╜¿╬─╝■╝╨ (2)/groundtruth/berlin_000032_000019_gtFine_labelIds.png]

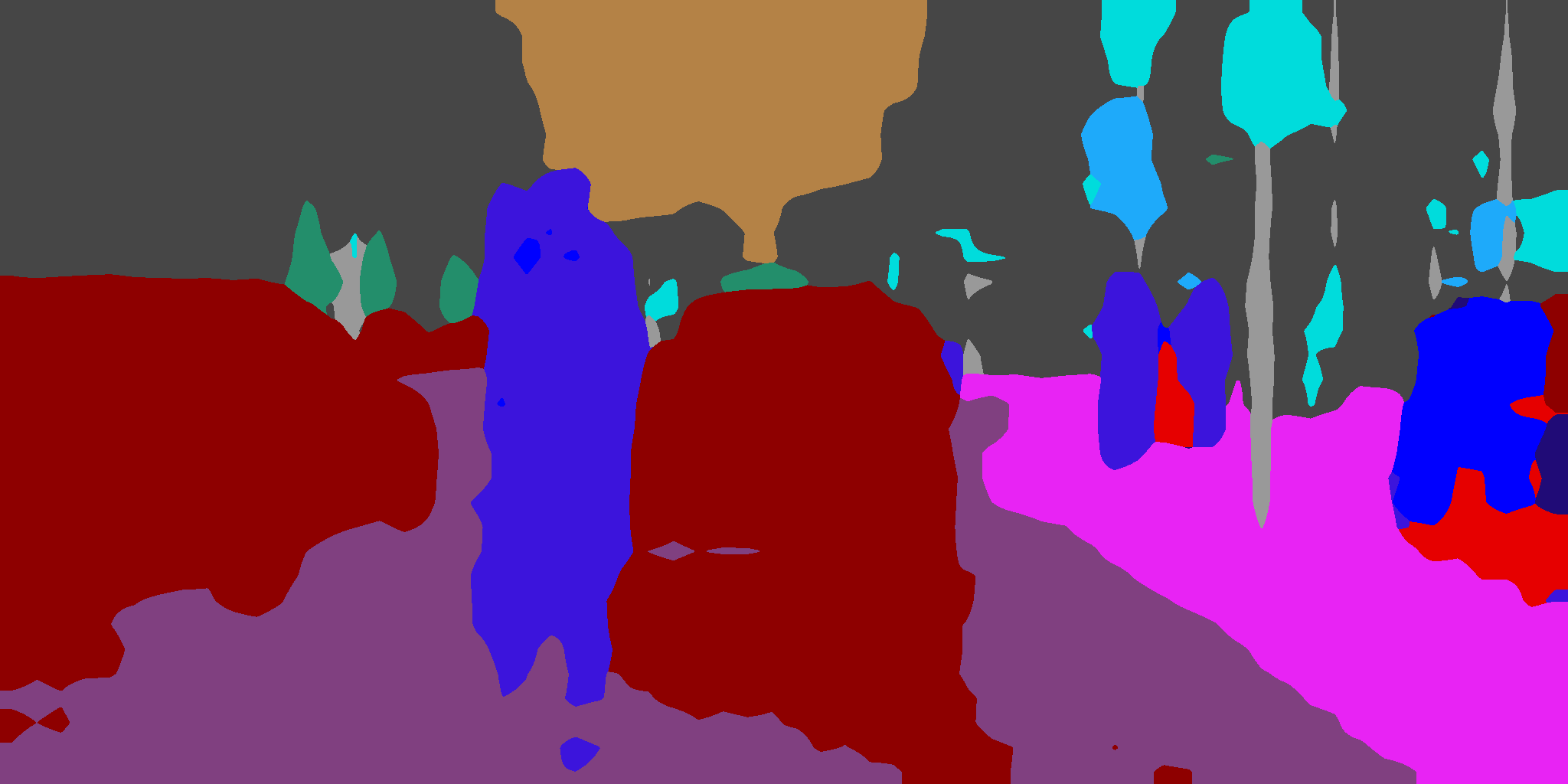

Supplement: S1 Data — (ZIP) [file pone.0295263.s001.zip › ╨┬╜¿╬─╝■╝╨ (2)/groundtruth/berlin_000032_000019_gtFine_labelTrainIds.png]
